# BacA: a possible regulator that contributes to the biofilm formation of *Pseudomonas aeruginosa*

**DOI:** 10.3389/fmicb.2024.1332448

**Published:** 2024-03-05

**Authors:** Lisa Wallart, Mohamed Amine Ben Mlouka, Brahim Saffiedine, Laurent Coquet, Hung Le, Julie Hardouin, Thierry Jouenne, Gilles Phan, Marie-Christine Kiefer-Meyer, Eric Girard, Isabelle Broutin, Pascal Cosette

**Affiliations:** ^1^Univ Rouen Normandie, INSA Rouen Normandie, CNRS, Normandie Univ, PBS UMR 6270, Rouen, France; ^2^Univ Rouen Normandy, INSERM US 51, CNRS UAR 2026, HeRacLeS PISSARO, Rouen, France; ^3^Paris Cité University, CiTCoM, CNRS, Paris, France; ^4^Univ Rouen Normandie, Normandie Univ, GlycoMEV UR 4358, SFR Normandie Végétal FED 4277, Innovation Chimie Carnot, RMT BESTIM, GDR Chemobiologie, IRIB, Rouen, France; ^5^Grenoble Alpes University, CNRS, CEA, IBS, Grenoble, France

**Keywords:** *Pseudomonas aeruginosa*, biofilm, rhamnolipids, proteome, crystallography, Psp system

## Abstract

Previously, we pointed out in *P. aeruginosa* PAO1 biofilm cells the accumulation of a hypothetical protein named PA3731 and showed that the deletion of the corresponding gene impacted its biofilm formation capacity. PA3731 belongs to a cluster of 4 genes (*pa3732* to *pa3729*) that we named *bac* for “Biofilm Associated Cluster.” The present study focuses on the PA14_16140 protein, i.e., the PA3732 (BacA) homolog in the PA14 strain. The role of BacA in rhamnolipid secretion, biofilm formation and virulence, was confirmed by phenotypic experiments with a *bacA* mutant. Additional investigations allow to advance that the *bac* system involves in fact 6 genes organized in operon, i.e., *bacA* to *bacF.* At a molecular level, quantitative proteomic studies revealed an accumulation of the BAC cognate partners by the *bacA* sessile mutant, suggesting a negative control of BacA toward the *bac* operon. Finally, a first crystallographic structure of BacA was obtained revealing a structure homologous to chaperones or/and regulatory proteins.

## 1 Introduction

*Pseudomonas aeruginosa* is an ubiquitous Gram-negative bacterium that infects a broad spectrum of hosts, including plants, animals, and humans (He et al., 2004). This microorganism has become the most problematic human opportunistic pathogen in immunocompromised patients especially in burn victims (Azzopardi et al., 2014) and in cystic fibrosis patients (Murphy, 2009; Folkesson et al., 2012). *P. aeruginosa* also causes severe nosocomial infections such as superinfections of surgical wounds, catheter-associated urinary tract infections, bacteraemia or ventilator-associated pneumonia. The pathogenicity of *P. aeruginosa* is multifactorial and mostly related to its ability to form biofilms. Indeed, this member of the ESKAPE group (*Enterococcus faecium*, *Staphylococcus aureus*, *Klebsiella pneumoniae*, *A. baumannii*, *Pseudomonas aeruginosa*, and *Enterobacter* spp.) possesses a remarkable ability to evade the immune system and is also refractory to antibiotic therapies (Høiby et al., 2011). Consequently, *P. aeruginosa* biofilm-related infections are associated with high morbidity and mortality.

Biofilms are bacterial communities embedded in a hydrated extracellular matrix (ECM) produced by the bacteria themselves. In the environment, bacteria are most frequently found in biofilms, attached to biotic or abiotic surfaces, because biofilm lifestyle presents a clear physiological advantage compared to planktonic growth. ECM acts as a protective barrier against harsh environments, host immune system clearance, starvation and antibiotic therapy (Wallart et al., 2023). Furthermore, biofilms are favorable to horizontal gene transfer, as bacteria are clustered together. As a result of gene exchange, bacteria increase their genetic diversity and enrich themselves with new determinants, such as genes involved in antibiotic resistance (O’Connell et al., 2006). *P. aeruginosa* is therefore considered by the World Health Organization (WHO) as a Priority 1 pathogen that urgently requires the development of new antibiotics.

Using a proteomic approach, we previously identified a group of proteins that accumulated in sessile *P. aeruginosa* PAO1 cells (Vilain et al., 2004), among which the protein of unknown function, referenced as PA3731. Additional study, showed that ∆*pa3731* mutant possesses a biofilm-defective phenotype, correlated with a decrease in rhamnolipid (RHL) secretion, in swarming motility and in virulence. We also reported that the *pa3731* gene was surrounded by 3 genes coding for proteins of unknown function. This cluster (*pa3732 to pa3729*) was named *bac* for “*biofilm associated cluster*” (Macé et al., 2008). In accordance with a high level of genomic conservation between PAO1 and PA14 strains (Mikkelsen et al., 2011), the *bac* cluster is also present in PA14 (sequence identity above 99%). In PA14, the *bac* system involves genes from *PA14_16140* to *PA14_16180*.

The PA3731 protein, renamed BacB, is annoted as a phage shock protein A (PspA). In accordance, BacB shares similarities with PspA (Macé et al., 2008). Indeed, as for PspA, BacB is exclusively helical and its abundance increased during an osmotic shock (Macé et al., 2008). PspA is one of seven proteins making up the Psp system in *Escherichia coli.* The *psp* locus is made up of (i) two divergently transcribed cistrons *pspABCDE* and *pspF* and (ii) the gene *pspG* (Brissette et al., 1990; Lloyd et al., 2004).

Bacterial cell adaptation to environmental changes is crucial for their survival. The Psp system is induced by numerous stress related to membrane integrity state as the mislocalization of secretins (Model et al., 1997), Sec and Tat secretion defects (Jones et al., 2003; DeLisa et al., 2004; Ize et al., 2004) or extra-cytoplasmic stresses as extreme heat, osmotic, oxidative or ethanol shocks. Because all of these stresses induced a dissipation of the proton motive force (PMF), PspA was supposed to sustain the PMF across the membrane (Kleerebezem et al., 1996). More recently, evidences showed membrane fusion and fission of artificial liposome in contact with PspA suggesting a possible role in the maintenance of a damaged membrane (Junglas et al., 2021; Liu et al., 2021).

Many Psp systems divergent from the *E. coli* Psp system have been discovered in other bacterial species, as the *sco2167-69* operon in *Streptomyces lividans* (Vrancken et al., 2008), the *pspA-ydj* operon, the *liaIHGFSR* in *Bacillus subtilis* (Yang et al., 2021) and the *clgRpspAMN* operon in *Mycobacterium tuberculosis* (Datta et al., 2015). Recently, evolution and genomic contexts of PspA across the bacterial clade were characterized using a large-scale bioinformatic approach (Ravi et al., 2023). This study revealed that PspA is well conserved across the bacterial clade, but not its cognate partners. Thus, variable genomic contexts surround PspA. The absence of homology between the Bac cognate partners and known Psp proteins is so not surprising. Thus, the BAC system could be another Psp system with a genomic environment divergent from that of the historical *E. coli* Psp system. However, as for *psp* regulon, a putative σ^54^-binding site upstream the *pa3732* (*bacA*) gene was identified (Brissette et al., 1991; Lloyd et al., 2004; Macé et al., 2008).

In the present study, we focused on the role of the PA14_16140 protein (i.e., BacA). Using a *bacA* mutant, we confirmed the involvement of the BAC system in several phenotypes related to biofilm formation and virulence. Proteomic and crystallographic approaches, allowed to suggest the function of BacA as a chaperone/regulator of the *bac* system. By RT-PCR, we reviewed the genomic organization of the *bac* system, and showed that the genes encoding it were more numerous (i.e., 6 instead of 4) and organized in operon.

## 2 Materials and methods

### 2.1 Bacterial strains and growth conditions

*P. aeruginosa* PA14 wild-type (WT) and its derivative transposon *16,140*::Tn mutant, named ∆*bacA*, were used. The mutant was obtained from a MAR2xT7 transposon mutant library which contains a gentamycin resistance cassette to ensure a selection pressure (Liberati et al., 2006). All bacterial precultures were performed in LB broth (Difco). For ∆*bacA*, gentamycin was added at a final concentration of 15 μg/mL.

### 2.2 Phenotypic experiments

#### 2.2.1 Planktonic growth kinetics

Free-cell cultures were performed in 250 mL Erlenmeyer flasks containing 50 mL of LB broth. Flasks were inoculated with fresh overnight culture at 10^7^ CFU/mL and incubated at 37°C under shaking at 140 rpm. The cell population was monitored every 30 min by optical density measurements at 600 nm (OD_600_) using a spectrophotometer (Cary 100 Bio, Varian). Data were expressed as mean values (±SEM) from at least three independent biological experiments.

#### 2.2.2 Swarming motility

Swarming motility was performed according to a protocol already described (Ha et al., 2014). Briefly, plates contained 25 mL of M9 broth (Sigma-Aldrich) (supplemented with 0.2% glucose, 0.5% casamino acids, 1 mM MgSO_4_) and 0.5% of technical agar (Difco). A 1.5 μL drop of fresh overnight precultures was placed in the center of the plate, touching the agar slightly. After 24 h at 37°C, the diameter (cm) of the circular zone of growth was measured. Data represent mean values (± SEM) from at least five independent biological experiments. Student’s *t*-test was performed to evaluate the statistical significance of the observed differences.

#### 2.2.3 Invading capacity

A549 human lung adenocarcinoma cells from the American type Culture Collection (ATCC) were grown as monolayer cultures in Dulbecco’s modified Eagle’s medium (DMEM) or in Ham’s F-12 Nutrient Mixture for at least 20 days, to allow differentiation to an alveolar type II (ATII)-like phenotype, as indicated, supplemented with 10% heat-inactivated fetal bovine serum and antibiotics (100 U/mL of penicillin G and 100 μg/mL of streptomycin) (Cooper et al., 2016). Cells were maintained at 37°C in a humidified atmosphere of 5% CO_2_. All cell culture media and supplements were purchased from ThermoFisher Scientific.

Then, cells were trypsinized and transferred to 24-wells plate to get a monolayer of 10^5^ cells per well. After 24 h of incubation under the same conditions, A549 cells were washed twice with 0.01 M phosphate buffer saline (PBS, Sigma-Aldrich), and fresh antibiotic-free medium was added. WT and ∆*16140* strains were added into the culture medium at a ratio of bacteria to host cells of 100:1 [multiplicity of infection (MOI) of 100]. Infected cells were incubated at 37°C under an atmosphere of 5% CO_2_ for 2 h.

To determine the number of adherent bacteria, cells were washed twice with PBS, lysed with 0.5% of Triton X-100, and the resulting suspension was serially diluted and plated onto LB agar (Difco). After an overnight incubation period at 37°C, colony forming units (CFU) were counted. To obtained the percentage of invading bacteria, the ratio between the initial and final CFU/mL was calculated. Data represent mean values (±SEM) from at least three independent biological experiments. Mann Whitney test was performed to evaluate the statistical significance of the observed differences.

#### 2.2.4 Quantification of CV-stained attached cells

Crystal violet (CV) assays was realized as previously described (Macé et al., 2008). Bacteria were grown for 2, 4, 6, and 8 h in LB broth at 37°C in microtiter dishes. Unattached cells were then removed by rinsing the microdishes thoroughly with water, and attached cells were subsequently stained by incubation with 0.5% CV for 20 min. CV was then solubilized by adding 1 mL of ethanol and the OD of the solution measured at 570 nm. Data represent mean values (±SEM) from at least three independent biological experiments. Two-way ANOVA test was performed to evaluate the statistical significance of the observed differences.

#### 2.2.5 Biofilm-ring test^®^

In addition to the CV-test, we evaluated the biofilm forming ability of the bacterial strains by the BioFilm Ring Test® (BioFilm Control SAS, Biopôle Clermont-Ferrand-Limagne). Bacteria were grown until an OD 600 of 1 (±0.05) and diluted 250 fold. The assay was carried out in modified polystyrene 8-wells microtiter plates as already described (Chavant et al., 2007; Macé et al., 2008). Wells containing LB broth inoculated with a bacterial suspension and magnetic beads were placed onto a magnetic block test. After magnet contact, free beads were attracted in the center of the bottom of wells, forming a black spot, while beads blocked by the biofilm remained in place. Images of each well before and after magnetization were compared with the BioFilm Control software that gives a BioFilm Indice (BFI). A high BFI (≥7) value corresponds to a high mobility of beads under magnet action (i.e., control wells) while a low value (≤2) corresponds to a full immobilization of beads (positive biofilm). Data represent mean values (±SEM) from at least four independent biological experiments. Two-way ANOVA test was performed to evaluate the statistical significance of the observed differences.

### 2.3 Microscopy experiments

#### 2.3.1 Biofilm formation and confocal laser scanning microscopy observations

Biofilm formation was achieved in a 4-well-chambered cell culture slide (System Nunc^™^ Lab-Tek^™^, ThermoFisher Scientific, United States). Briefly, 1 mL of bacterial suspension in PBS were transferred to each well at 10^6^ CFU/mL. The slide was incubated at 37°C without shaking in the darkness. After 2 h, PBS was substituted by LB medium, and slide was incubated at 37°C without shaking in the darkness.

At specific incubation times, the medium was removed, and the biofilms were washed twice with PBS. Biofilms were finally stained with Syto9 (ThermoFisher Scientific, United States) for 20 min, following the manufacturer protocol, prior to microscopy experiments. The images were acquired using a Leica TCS SP8 CFS confocal microscope, with fixed stature (Leica Microsystems, Nanterre, France), equipped with a laser diode (Coherent, Les Ulis, France) at 488 nm. Fluorescence emission was detected sequentially by a hybrid detector (Leica Microsystems, Nanterre, France) in photon counting mode with a specific band-pass filter from 500 to 540 nm. Image processing was performed with Imaris software (Bitplane, Switzerland). Data represent mean values (±SEM) from at least three independent biological experiments. Mann Whitney test was performed to evaluate the statistical significance of the observed differences.

#### 2.3.2 Measurement of A549 cell density

A549 human lung adenocarcinoma cells (ATCC) were grown as monolayer cultures on 0.8 cm round slides placed in a 24-wells plate, and infected by the *P. aeruginosa* strains, according to the conditions described above (see “2.2.3 Invading capacity”). Before microscopic observations, round slides were washed three times with PBS and stained with DAPI and ActinRed^™^ 555 ReadyProbes^™^ (ThermoFisher Scientific, United States) for 20 min. The images were acquired using a Leica TCS SP8 CFS confocal microscope with a ×20 objective. DAPI and ActinRed were excited at 405 and 552 nm, respectively. Fluorescence emission was detected sequentially by a hybrid detector in photon counting mode with specific band-pass filters from 500 to 540 nm for DAPI and 570 to 620 nm for ActinRed. Image processing was performed with Imaris software. The cellular density was automatically quantified with the “spot fluorescent detection” option. Data represent mean values (±SEM) from at least 10 acquisitions, from four independent biological experiments. Mann Whitney test was performed to evaluate the statistical significance of the observed differences.

### 2.4 Rhamnolipids quantification

#### 2.4.1 Sample preparation

A 500 mL Erlenmeyer flask containing 100 mL of LB was inoculated with fresh overnight precultures at 10^7^ CFU/mL. Cultures were performed at 37°C under shaking at 140 rpm. At specific incubation times, cells were enumerated by conventional dilution series and plating techniques onto LB agar plate to evaluate the number of CFU/mL, and 1 mL of the culture was collected and centrifuged at 9,000 × g for 15 min. The supernatants were filtered with a 0.22 μm PVDF membrane filter (Millex-GP, Merck Millipore Ltd., Ireland). Filtered supernatants were stored at −20°C until mass spectrometry analyses. Supernatants collection was performed from three independent biological experiments.

#### 2.4.2 LC–MS/MS

The supernatants collected between 2 h and 12 h and at 24 h and 48 h of incubation, were diluted 2- and 10-fold, respectively. LC–MS/MS analytical conditions were adapted from methods previously described (Déziel et al., 2000; Bazire et al., 2005; Sauvage et al., 2022). Analysis of biosurfactants was performed with a 1,290 Infinity II UHPLC system coupled to a 6545XT Advance-Bio Q-TOF mass spectrometer (Agilent Technologies, United States). One μL of diluted supernatant was injected and the separation was carried using a reverse-phase with a Zorbax SB-C18 Rapid Resolution column (4.6 mm × 150 mm, 3.5 μm particle size, 80 Å pore size, Agilent, United States). The mobile phases were water (phase A) and acetonitrile/water (90/10) (phase B) with 4 mM ammonium acetate in each mobile phase. The chromatographic separation was conducted at 50°C with a flow rate of 750 μL/min during 20 min by using the following gradient: 3 min at 40% B - 13 min from 40 to 90% B (linear gradient) - 2 min at 90% B - 12 s from 90 to 40% B and 1 min 48 s at 40% B. LC–MS/MS analyses were performed in negative mode on a mass range of 50–1,500 *m/z*. The parameters set for the analysis were 200°C for the gas temperature in source, 3.5 kV and 175 V for the capillary and fragmentary voltage, respectively. The MS2 fragmentation was performed in collision-induced dissociation (CID) mode with a collision energy setting ranging from 5 to 25 V, depending on the rhamnolipid congener.

### 2.5 Biofilm proteomic analyses

#### 2.5.1 Protein extraction

Biofilm formation was achieved in a 4-well-chambered cell culture slide as described above (see “2.3.1 Biofilm formation and confocal laser scanning microscopy observations”). After 48 h of incubation, LB medium was removed, and biofilms were washed twice with PBS. Biofilms were then detached from the slide using a sonication bath with denaturing buffer (7 M urea, 2 M thiourea, tri-N-butylphosphine (TBP) 2 mM, dithiothreitol (DTT) 20 mM, 0.5% (w/v) 3-[(4-heptyl)phenyl-3- hydroxypropyl]dimethylammoniopropanesulfonate (C7BzO), 2% (w/v) 3-[(3-cholamidopropyl)dimethylammonio]-1-propanesulfonate hydrate (CHAPS)). The bacterial suspension was freeze-thawed for three cycles and then sonicated on ice for 10 min, before the lysate was centrifuged at 12,000 × g for 30 min at 4°C, to separate cellular debris and soluble proteins. Protein concentrations were evaluated using the Bradford assay (Bio-Rad). Samples were stored in aliquots at −20°C until further use. Protein extraction was performed from three independent biological experiments.

#### 2.5.2 Pre-analytical treatment

For each extract, 25 μg of proteins were mixed with SDS loading buffer [62 mM Tris–HCl pH 6.8, 20% glycerol (v/v), 0.04% bromophenol blue (w/v), 0.1 M DTT, SDS 4% (w/v)] and loaded onto a 7% polyacrylamide gel (Acrylamide/Bis-Acrylamide 30% [29:1], Sigma-Aldrich). A short period of electrophoresis was performed (90 min at 10–20 mA/gel) to concentrate proteins. After migration, gels were stained with Coomassie Blue G250. For each sample, the revealed protein band was excised and firstly immersed in a reductive buffer (5 mM DTT, Sigma-Aldrich) and then in an alkylating buffer (20 mM iodoacetamide, Sigma-Aldrich). Gel bands were washed with 50% acetonitrile (ACN) (Fisher, Hampton, NH) / 50% ammonium bicarbonate 10 mM (Sigma-Aldrich) and dried 3 times with 100% ACN. Proteins were then digested with 1 μg of trypsin (Promega, Madison, WI), overnight, at 37°C and several steps of peptide extraction were performed. Finally, peptides were dried in a SpeedVac concentrator (ThermoFisher Scientific, United States) at 30°C and stored at −20°C until mass spectrometry analysis.

#### 2.5.3 Nano LC–MS/MS

Peptides were solubilized in 0.1% formic acid (FA) (v/v) and analyzed using mass spectrometry. All the experiments were performed using a Q-Exactive Plus equipment (Thermo Scientific, Waltham, MA, United States) coupled with an Easy-nLC 1,200 (Thermo Scientific). One μL of sample (0.2 μg) was injected and first eluted toward an enrichment column (C18 PepMap100, Thermo Scientific). The separation was then performed with an analytical column needle (NTCC-360/internal diameter: 100 μm; particle size: 5 μm; length: 153 mm, NikkyoTechnos, Tokyo, Japan). The mobile phases consisted of H_2_O/0.1% formic acid (FA) (buffer A) and CH_3_CN 80%/H_2_0 20% with 0.1% FA (buffer B). The tryptic peptides were eluted at a flow rate of 300 nL/min using a three-step linear gradient: from 2 to 55% of buffer B over 106 min, from 55 to 100% of buffer B over 4 min, and 100% of buffer B for 15 min. The mass spectrometer was operated in the positive ionization mode with the capillary voltage and the source temperature set at 1.8 kV and 275°C, respectively. The samples were analyzed using the higher-energy collisional dissociation (HCD) method. The first scan (MS spectra) was recorded using the Orbitrap analyzer (*R* = 70,000) in the mass range of m/z 400–1,800. AGC target and maximum injection time were set to 3.10^6^ and 60 ms, respectively. Then, the 10 most intense ions were selected for tandem mass spectrometry (MS2) experiments, excluding singly charged species. The dynamic exclusion of the already fragmented precursor ions was carried out for 20 s. Fragmentation occurred in the HCD cell analyzer at a normalized collision energy of 28. The MS2 spectra were also recorded using the Orbitrap analyzer at a lower resolution (*R* = 17,500).

#### 2.5.4 Protein quantification

After MS analysis, raw data were imported into Progenesis LC–MS software (Nonlinear Dynamics, version 4.0.4441.29989, Newcastle, UK). For the comparison, one sample was set as a reference, and the retention times of all the other samples within the experiment were aligned. After alignment and normalization, a statistical analysis was performed for one-way analysis of variance (ANOVA) calculations. For quantitation, peptide features presenting a *p*-value and a *q*-value of less than 0.05 and a power of more than 0.8 were retained. The MS/MS spectra of the selected peptides were exported for peptide identification with Mascot (Matrix Science, version 2.2.04) against the database of *Pseudomonas aeruginosa* UCBPP-PA14 - Assembly GCF_000014625.1 from NBCI. Database searches were performed with the following parameters: 1 missed trypsin cleavage and as variable modifications: carbamidomethylation of cysteine and oxidation of methionine. The mass tolerances for the precursor and fragment ions were set at 5 ppm and 0.02 Da, respectively. False discovery rates (FDR) were calculated using a decoy-fusion approach. The identified peptide-spectrum-matches (PSM) with a -10logP of 20 or higher were kept at an FDR threshold of 1.5%. The Mascot search results were then imported into Progenesis for further processing. For each condition, the total cumulative abundances of the proteins were calculated by summing the abundances of the corresponding peptides. Proteins identified with less than 2 peptides were discarded and only proteins with a fold change equal to or greater than 1.5 in their average normalized abundances between both strains were retained. The raw files and quantification data were deposited in the ProteomeXchange data repository (accession number PXD044736).

### 2.6 DNA manipulation

#### 2.6.1 Mutant verification

The good insertion of the transposon MAR2xT7 in the *PA14_16,140* gene was controlled by PCR. The primers (Supplementary Table S1) were designed to target the transposon insertion site. For the *PA14_16,140* WT gene region, primers amplify from upstream to downstream intergenic region (PCR product: 478 pb). For the mutant, primers were used to amplify the intergenic sequence to the other side of the transposon sequence (PCR product: 1472 pb). PCR was performed from one colony, for 30 cycles of 5 min at 95°C, 30 s at 95°C, 30 s at 55°C and 1 min per kb at 72°C. The resulting PCR products were loaded onto 1% agarose gel stained with SYBR^™^ Safe (Invitrogen, ThermoScientific, United States) and visualized using a Chemidoc^™^ Imaging System (Biorad).

#### 2.6.2 Reverse transcription polymerase chain reaction

Total RNA was isolated from 10^9^ bacterial cells with the PureLink^™^ RNA Mini Kit (Invitrogen, ThermoFisher Scientific, United States) following the manufacturer protocol. To minimize the possibility of DNA contamination, RNA samples were treated with DNAse using the TURBO DNA-free^™^ Kit (Invitrogen, ThermoFisher Scientific, United States) following the manufacturer protocol. The amount of RNA was quantified by Nanodrop™ (ThermoFisher Scientific, United States). Aliquot of 2 μg of RNA were reverse transcribed into cDNA using the High Capacity cDNA Reverse Transcription Kit (Applied Biosystems, ThermoFisher Scientific, United States), according to the manufacturer protocol using random primer as initiation primer in a final reaction volume of 20 μL. Next, retro-transcription reactions were subjected to PCR amplification using specific primers (Supplementary Table S1) targeting the end of the first gene and the beginning of the second gene. The resulting PCR products were loaded onto 1.5% agarose gel stained with SYBR^™^ Safe (Invitrogen, ThermoScientific, United States) and visualized using a Chemidoc^™^ Imaging System (Biorad).

### 2.7 BacA structure determination

#### 2.7.1 BacA production and purification

Briefly, the DNA coding *pa3732* was inserted into pET resulting in a vector called pET-*16140*. The plasmid was transformed into *E. coli* BL21 λ(DE3) pLysS and contains a carbenicillin resistance cassette and an IPTG receptor to induce transcription. Cells harboring the expression vector were positively selected onto Luria-Bertani agar plates containing 100 μg/mL of carbenicillin.

The final product contains a N-terminal 6His tag for the purification by IMAC method. A single clone was picked and inoculated into 2 × 50 mL LB broth (Roth) for overnight growth. A 6 × 1 L LB culture was inoculated at OD_600_ = 0.01 with the overnight culture, and the expression was induced with the addition of 1 mM IPTG when OD_600_ reached 0.6. After 3 h at 37°C, cells were harvested by centrifugation at 5,000 × g for 15 min at 4°C and resuspended in 5 mL of lysis buffer (20 mM Tris–HCl, pH 8.0, 150 mM NaCl). The bacterial suspension was freeze-thawed for three cycles and then sonicated on ice for 10 min. The lysate was centrifuged at 50,000 × g for 20 min at 4°C to separate the cellular debris and the soluble proteins. To check the induction, 5 μg of proteins were mixed with sodium dodecyl sulfate (SDS) loading buffer [62 mM Tris–HCl pH 6.8, 20% glycerol (v/v), 0.04% bromophenol blue (w/v), 0.1 M DTT, SDS 4% (w/v)] then loaded onto a 12% SDS–PAGE gels.

The supernatant was applied on an affinity column HisTrap HP^™^ (GE healthcare) which was pre-equilibrated with the loading buffer (20 mM Tris–HCl pH 8.0, 200 mM NaCl). After washing steps with increasing percentages of elution buffer (20 mM Tris–HCl, pH 8.0, 200 mM NaCl, 400 mM imidazole), the protein was eluted with 100% of the elution buffer. After sample dialysis and thrombin cleavage to remove the signal peptide, the resistance cassette and the 6His tag, the sample was concentrated on a 10 kDa cut-off Amicon^™^ (Merck) and loaded on a Superdex^™^ 75 10/300 (GE healthcare) gel filtration column for further purification. The protein was eluted in one major peak that corresponded to the size of a dimer. The purity of the eluted fractions was verified by SDS-PAGE, and the eluted fractions presenting the highest purity were pooled and concentrated on Amicon™ 10 kDa to a concentration of 20 mg/mL.

A large screening for crystallization conditions was performed with the kits Crystal Screen^™^ (Hampton Research), JCSG I^™^, JCSG II^™^, JCSG III^™^, and JCSG IV^™^ (Molecular Dimensions Limited) by using a nanodrop Mosquito^™^ (TTP Labtech) automate. Several hits were obtained in different conditions, with size improvement of the crystals when mixing the protein with the terbium complex Tb-Xo4 “crystallophore,” a component that modifies the solubility of the proteins and can be used for phasing by single-wavelength anomalous dispersion (SAD) (Engilberge et al., 2017). After manual optimization of several hits, crystals were obtained in 32% PEG 200, 0.2 M NaCl and 0.1 M acetate pH 4.5 with a 1/1 drop to reservoir ratio. Before cryo-cooling they were transferred in the same condition supplemented with 20% (v/v) glycerol.

#### 2.7.2 X-ray data collection and integration

Diffraction data were recorded at the SOLEIL synchrotron (Saclay, France) on PX1 beamline (Coati et al., 2017) with a Eiger-X 16 M (Dectris Ltd.), at a wavelength of 1.65 Å as to maximize the anomalous signal of Tb. The data sets were indexed and merged with the XDS program (Kabsch, 2010) in the P6(5)22 space group. Data sets were scaled and merged with the POINTLESS/AIMLESS programs (Evans, 2006), part of the CCP4 suite (Collaborative Computational Project, Number 4, 1994; Winn et al., 2011) and converted to structure factors with the TRUNCATE program (Evans, 2011). The statistics of the best data collection are reported in Supplementary Table S2.

#### 2.7.3 Structure determination and refinement

Refinement of the BacA-Tb-Xo4 structure was performed using the Phenix program (Liebschner et al., 2019). The anomalous differences for the Tb-Xo4 were sufficient to obtain the phases by SAD with Autosol, and the first model was built by the Autobuild procedure (Terwilliger et al., 2008). Standard constraints were used throughout on distances, angles, planes and isotropic factors. The Coot graphic program (Emsley and Cowtan, 2004) was used for rebuilding of the models and water/ligands localization all along the different refinement cycles. All the statistics regarding the final refined structure are also reported in the Supplementary Table S2. The coordinates and structure factors of BacA-Tb-Xo4 were deposited in the PDB (accession number 8Q8O). All figures were drawn with PyMOL (Delano, 2002).

## 3 Results

### 3.1 Disruption of *bacA* gene leads to a decrease of rhamnolipids secretion

**Figure 1:**
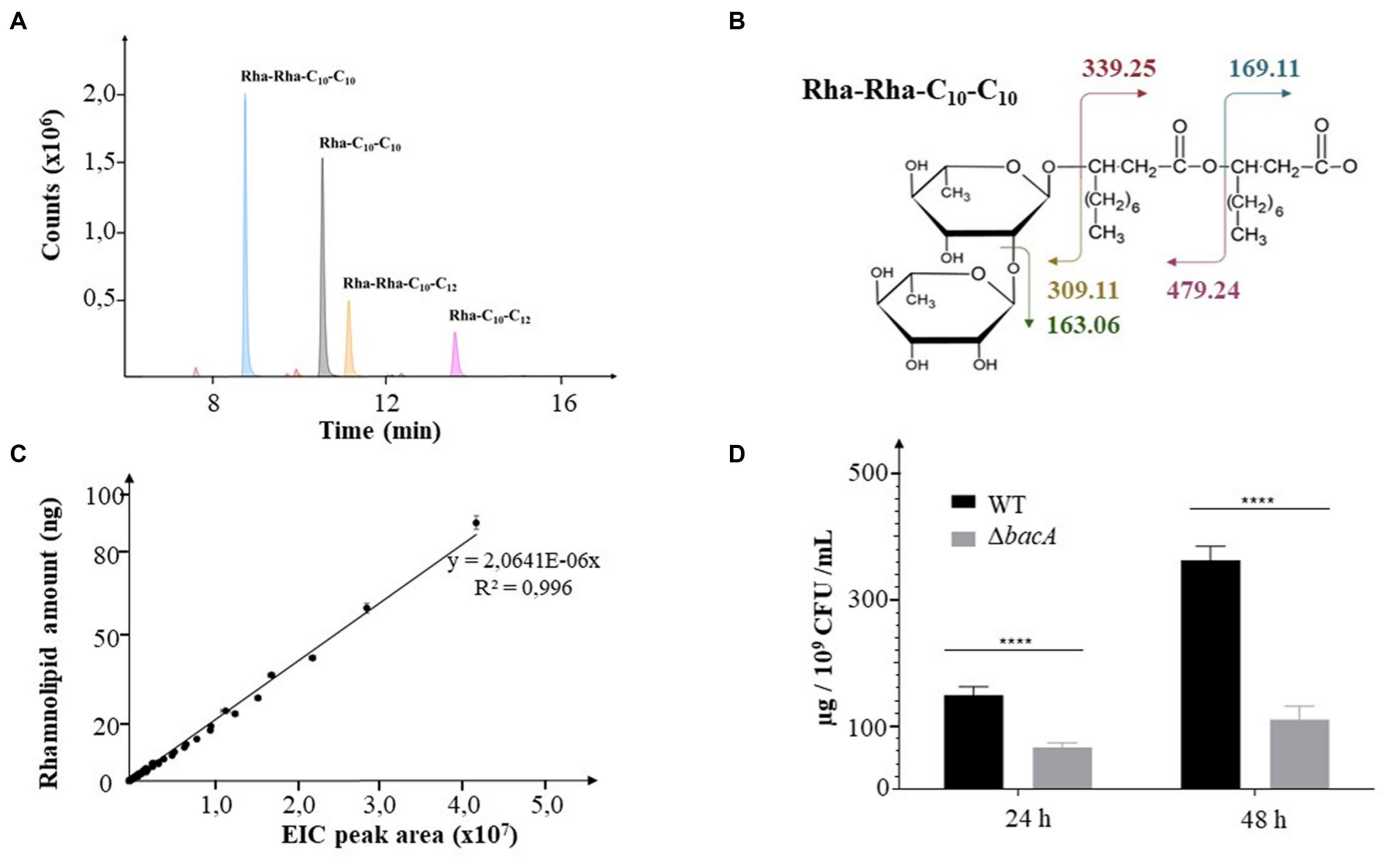
Decrease of rhamnolipid secretion. **(A)** Extracted Ion Chromatograms (EIC) peaks detected for the most abundant rhamnolipids collected from bacterial cultures. **(B)** Molecular representation of the expected fragmentation pattern of the Rha-Rha-C_10_-C_10_ (*m/z*: 649.38). **(C)** Calibration curve obtained from commercial rhamnolipids. The EIC areas were measured from MS data for all detected rhamnolipids injected at concentrations covering nearly 2 orders of magnitude (2.5, 5, 10, 15, 20, 25, 30, 40, 50, 75, 100 ng). **(D)** Rhamnolipids quantification in supernatants of 24 h or 48 h culture of the WT and the Δ*bacA* strains. Data are expressed as mean values (±SEM) from three independent biological experiments (****p < 0.0001).

Rhamnolipids secreted by the WT and the mutant strains were identified and monitored by LC–MS/MS. RHLs were quantified by integrating the extracted-ion chromatogram (EIC) peaks of respective [M-H^−^] *m/z* for each congener from MS spectra (see example Figure 1A). Their identifications were confirmed by the MS2 data resulting from the fragmentation of the MS ions with *m/z* values expected for each glycolipid (see example Figure 1B). The relative abundances of RHLs were expressed by calculating the ratio of their respective EIC peak areas to the total EIC peak area for all detected rhamnolipids. For relative quantification, the respective values of EIC peak areas were reported on a linear calibration curve established with commercial rhamnolipids (R95MD, Sigma-Aldrich) (Figure 1C). Finally, to normalize the data with respect to the cellular density in the culture, the quantity of rhamnolipid was related to the number of CFU/mL.

The transposon insertion into the *PA14_16,140* gene did not change the growth ability of the mutant (Supplementary Figures 1A,B, respectively). Whatever the strain, RHLs started to be detected during the log phase (6 h) and their quantity increased with time, reaching a maximum at the end of the stationary phase (48 h). During the stationary phase, the mutation negatively affected the RHL secretion (24 h and 48 h, *p* < 0.0001) (Figure 1D, Supplementary Figure 2A). Thus, at 24 h and 48 h of culture, the mutant secreted 50 and 63% less rhamnolipids, respectively.

We then monitored the amount of the two mostly abundant rhamnolipids, i.e., the Rha-C_10_-C_10_ (noted R1) and the Rha-Rha-C_10_-C_10_ (noted R2). At 24 h of incubation, we, respectively, observed a decrease of 60% (*p* < 0.05) and 40% (*p* < 0.05) of their production in the mutant compared to the WT strain. After 48 h, this behavior was emphasized with a decrease of 70% (*p* = 0.01) and 56% (*p* < 0.05), respectively (Supplementary Figure 2B).

### 3.2 *ΔbacA* is impaired in swarming motility

**Figure 2:**
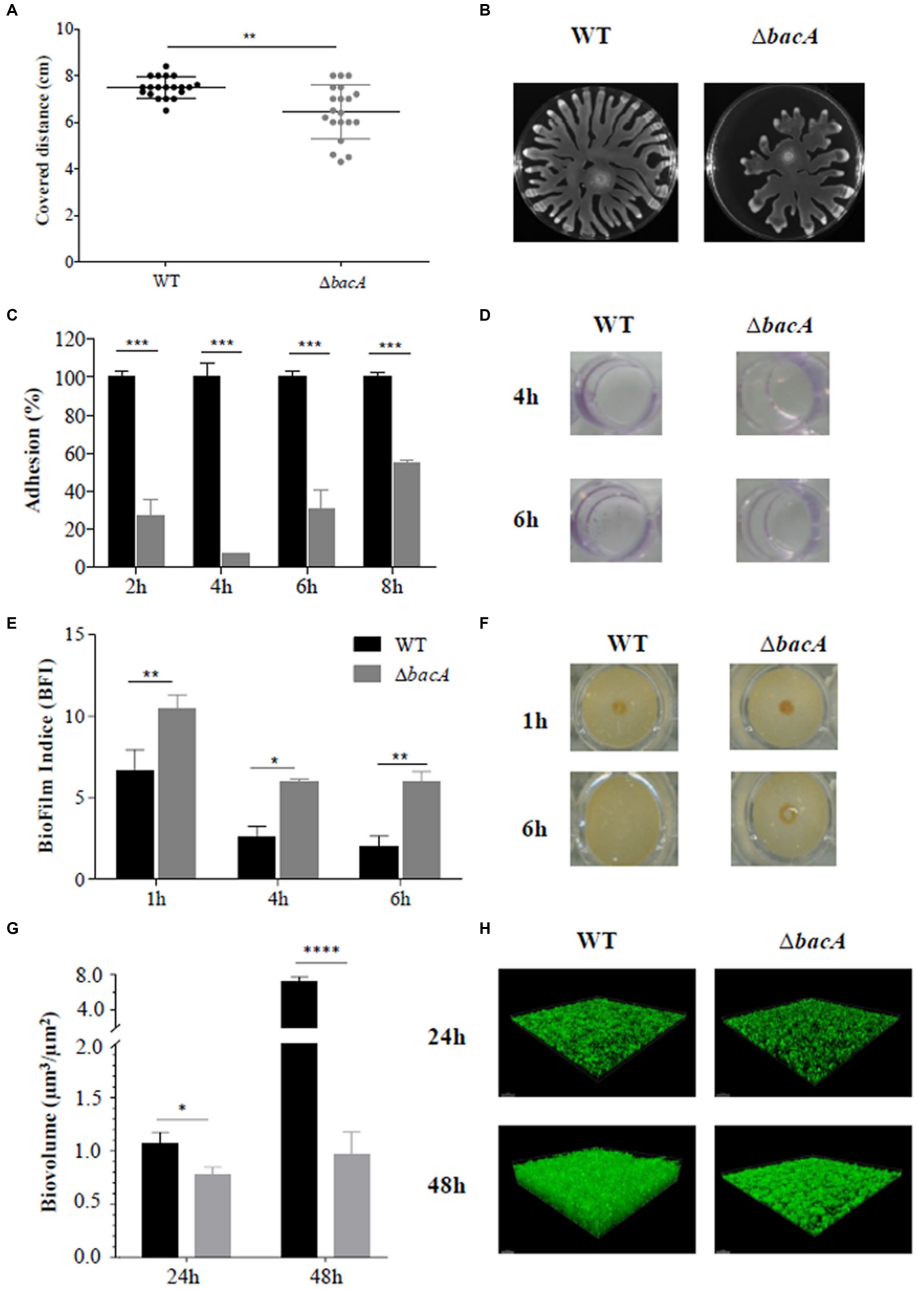
Differential phenotypic traits. **(A)** Swarming motility assay in agar plates after 16 h of incubation at 37°C. **(B)** Resulting covered distances for the WT and ∆*bacA* strains (**p < 0.01). **(C)** Percentage of adhesion based on the crystal violet test of the WT and Δ*bacA* strains. The 100% value represents the OD value obtained with the WT. Data represent mean values (±SEM) from at least three independent biological experiments (***p < 0.001). **(D)** Representative images of the crystal violet coloration obtained for the WT and Δ*bacA* strains after 4 h and 6 h of incubation. **(E)** Quantification of the biofilm formation by the WT and ∆*bacA* strains using the Biofilm Ring Test^®^. Data represent mean values (±SEM) from at least four independent biological experiments (ns: non-significant; *p < 0.05; **p < 0.01). **(F)** Representative images of the migration of the magnetic beads in contact with the WT and Δ*bacA* strains after 1 h and 6 h of incubation. **(G)** Quantitative comparison of biofilm biovolume (μm^3^/μm^2^) based on the integration of microscopy images. Data represent mean values (±SEM) from at least three independent biological experiments (ns: non-significant; *p < 0.05; ****p < 0.0001). **(H)** Representative confocal microscopy images of the WT and ∆*bacA* biofilms labeled with Syto9.

The swarming motility is an important group behavior, essential to promote biofilm formation in *P. aeruginosa*. Moreover, RHLs, as surface-active molecules, act as wetting agents during the swarming motility (Caiazza et al., 2005). Consequently, the swarming motility of Δ*bacA* was compared to that of the WT strain. As shown in Figures 2A,B, swarming motility was significantly reduced in the mutant compared with the WT (*p* < 0.01). Furthermore, the classical profile of *P. aeruginosa* expansion on the Petri dish appeared unstructured for the mutant. The shape of the dendritic ends was highly disordered, an observation that could be correlated with a lack of wetting agents such as rhamnolipids (Kearns, 2010).

### 3.3 Mutation of *bacA* affects biofilm formation

Considering the role of RHLs and swarming motility during the biofilm formation, the ability of the mutant to form biofilms was investigated. Crystal violet staining (Figures 2C,D) revealed a significant (*p* < 0.001) decrease of the attached cells as compared with the WT at all the incubation times tested. These first results were completed by the Biofilm Ring test^®^ (Figures 2E,F). Images obtained with the parent strain and *ΔbacA* confirmed the alteration in the biofilm-forming ability of the mutant (Figure 2F). After 1 h of incubation, biomass accumulation can already be observed in the WT strain (BFI of 6.63 ± 1.30) whereas a BFI value of 10.45 ± 0.86 was obtained with *ΔbacA* (*p* < 0.01). This alteration to form a biofilm by the mutant was still observed at 6 h (*p* < 0.01) (Figure 2E).

This biofilm defective phenotype of *ΔbacA* was confirmed by measurement of the biofilm biovolume after CLSM observations. At 24 h and 48 h, the biofilm biovolume of the mutant was significantly decreased by 28% (*p* < 0.05) and 86% (*p* < 0.0001), respectively, in comparison with the WT (Figures 2G,H).

Bacterial adherence to eukaryotic cells is crucial to induce a chronic infection since it is the first step of the biofilm formation. The non-adhesive phenotype of the mutant was also observed in contact with the A549 cells which exhibited an invading ability of 55% (p < 0.01) as compared with the WT (Supplementary Figure S3A). Moreover, as shown on Supplementary Figures S3B,C, a significant loss of cellular layer density was observed after contact with the WT compared to the mutant (*p* < 0.01).

### 3.4 The Δ*bacA* proteome revealed an increased abundance of the BAC cognate partners

**Figure 3:**
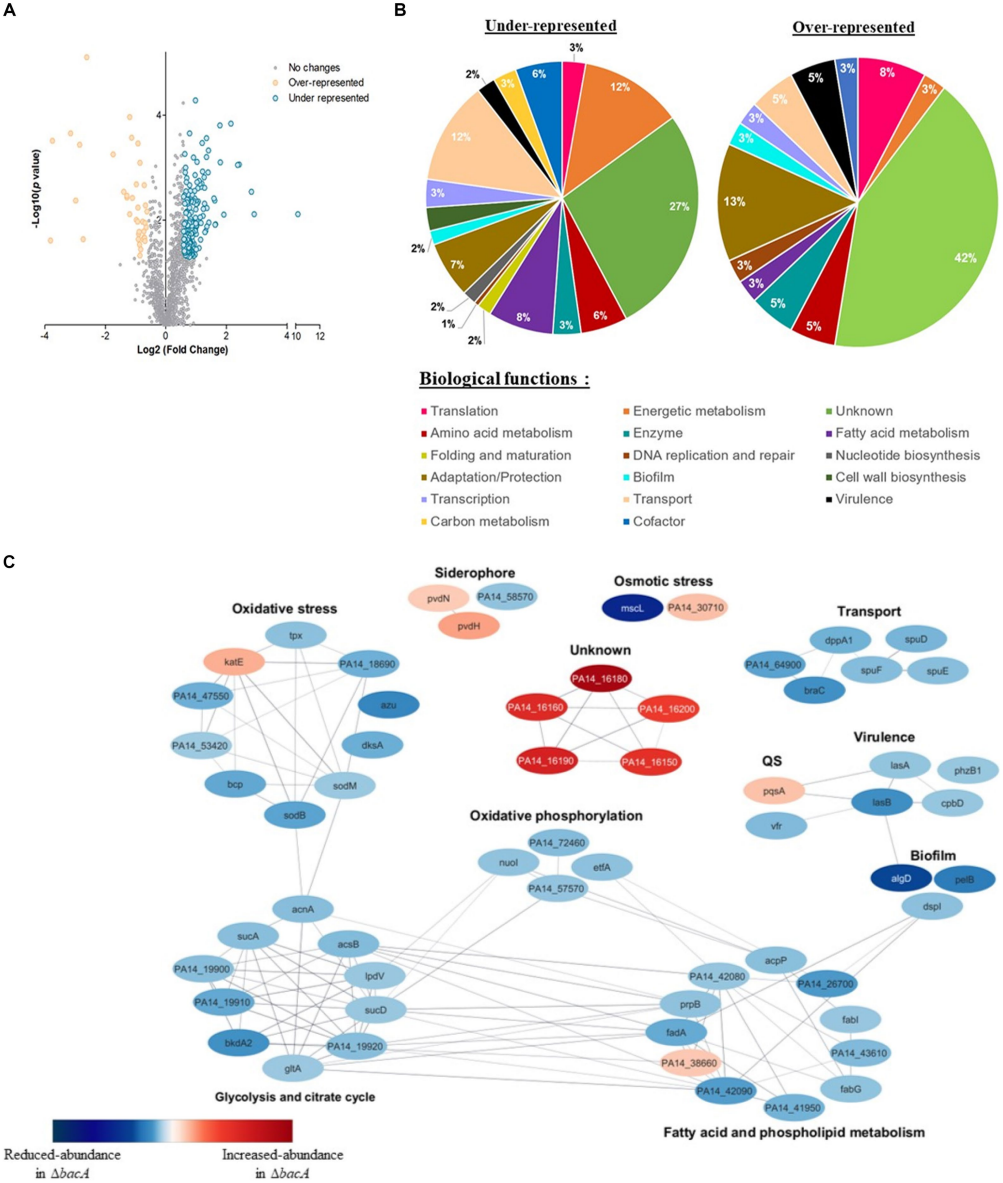
Differential quantitative proteomic analyses of WT strain and ∆*bacA* mutant grown as biofilms. **(A)** Volcano plot representation of differential proteomics analysis. Circles representing proteins are placed according to their statistical *p*-value and their relative abundance ratio (log 2-fold change). Gray circles correspond to proteins that do not present significant differential abundance. **(B)** Classification of the differentially represented proteins (under-represented on the left and over-represented in the right) according to their biological processes using the Kyoto Encyclopedia of Genes and Genomes (KEGG) pathway database. **(C)** Protein–protein interaction network for proteins displaying the most differential abundances between the biofilm formed by the WT or ∆*bacA* strains. Nodes are colored according to protein abundance: red color corresponds to accumulated proteins in the mutant whereas blue color is used for proteins of decreased abundance. Edges indicate protein–protein interactions.

To help deciphering the functional role of BacA, we performed a differential proteomic analysis between the WT and the *bacA* mutant grown as biofilm. More than 1700 proteins were identified, among which, 205 showed a significant variation of abundance (fold >1.5 and *p* < 0.05): 38 proteins were increased and 167 proteins were diminished in the mutant (Figure 3A, Supplementary Table S3).

Based on PseudoCAP functional classes and KEGG pathways, proteins presenting decreased abundance were involved in different processes, including bacterial fitness, transport, fatty acid metabolism and energetic metabolism (Figure 3B). Concerning accumulated proteins, a strong enrichment of proteins involved in adaptation and protection was observed (Figure 3B).

To identify functional connections between the proteins differentially produced, we used this subset of proteins to construct a protein–protein interaction network. The latter was first constructed using the String database, by selecting connections over a threshold of 0.6 of confidence. The resulting network was visualized within Cytoscape (version 3.10.0) (Figure 3C). We observed a high number of interactions between proteins whose amount decreased. They were involved in the superpathways of glycolysis, pyruvate metabolism, tricarboxylic acid cycle (TCA) and oxidative phosphorylation. Within this profile, we also noted a reduced abundance of proteins associated with transport, virulence, matrix synthesis and prominently oxidative stress response (Figure 3C, Supplementary Table S3).

The proteome of the mutant pointed out an increase of a few tens of proteins. Interestingly, among the proteins which exhibited by far the highest folds, 5 caught our attention: (i) BAC proteins, i.e., PA14_16150 (BacB), 7.2-fold; PA14_16160 (BacC), 7.9-fold; PA14_16180 (BacD), 13.5-fold and (ii) two additional proteins: PA14_16190, 8.9-fold and PA14_16,200, 6.1-fold, which are encoded by genes which are just downstream the *bac* genes.

Based on the common behavior of these 6 proteins in the Δ*bacA* mutant, we hypothesized that the bac system would be in reality composed of these 6 genes. In this case, BacA could be a key protein for the expression of the BAC system.

### 3.5 Organization of the BAC system

**Figure 4:**
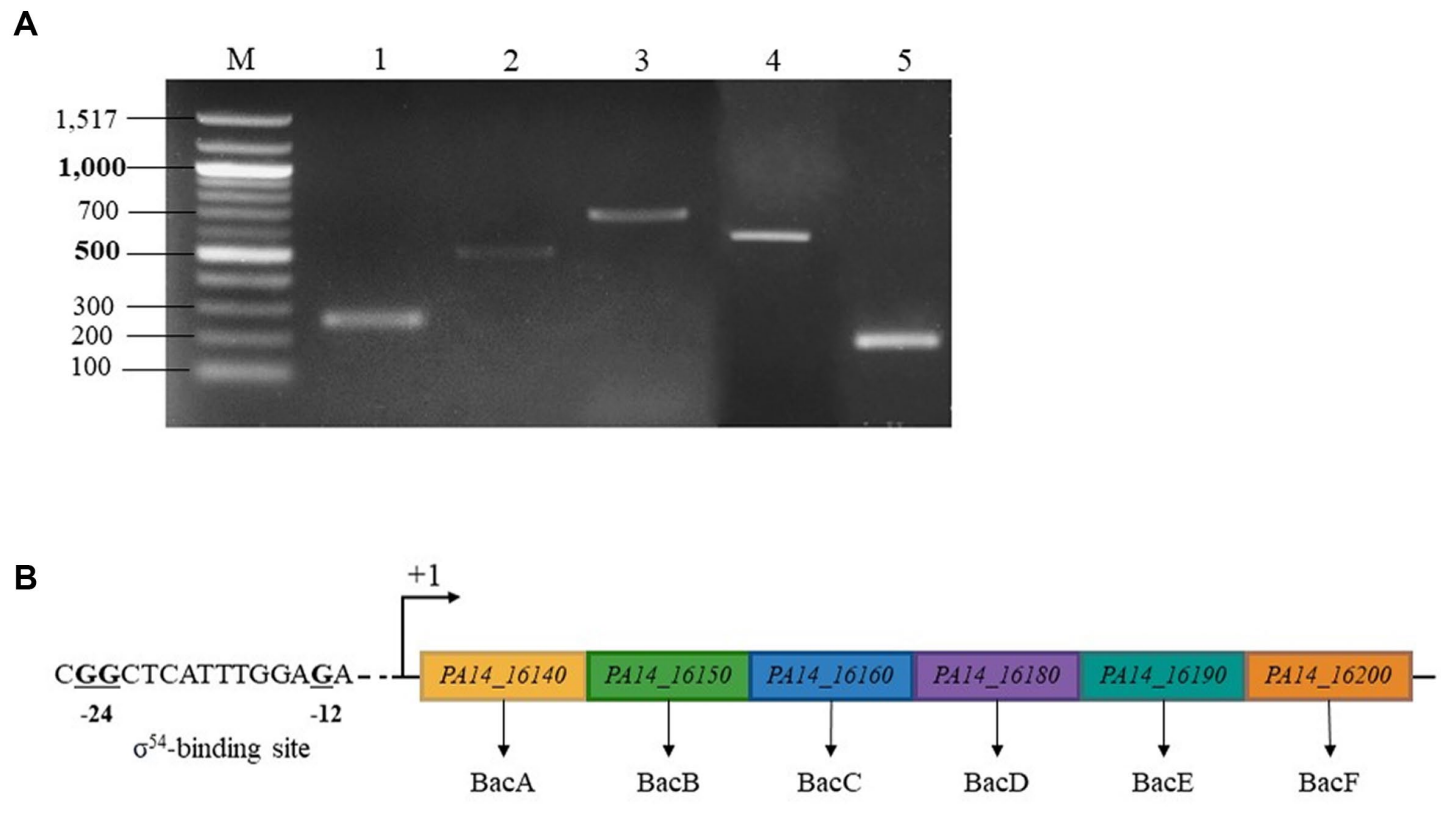
BAC system is coding by an operon made up by 6 genes and regulated by a σ^54^ (RpoN) sigma factor. **(A)** Electrophoretic gel from RT-PCR experiment. Lane 1: transcript *bacA*-*bacB*, lane 2: transcript *bacB*-*bacC*, lane 3: transcript *bacC*-*bacD*, lane 4: transcript *bacD*-*PA14_16190*, lane 5: transcript *PA14_16190*–*16,200*, lane M: 100 bp ladder molecular size marker (bp). **(B)** σ^54^ sigma factor -binding sites of *P. aeruginosa PA14_16,140* (*bacA*). The highly conserved −24 and −12 dinucleotides are shown in bold.

To examine *bac* genes organization, we analyzed the transcription of genes from *PA14_16140 (bacA)* to *PA14_16200* in the WT. According to the Figure 4A, a specific amplification product was detected at each intergenic region separating *bacA, bacB, bacC, bacD, PA14_16190* and *PA14_*16200. In conclusion, the BAC system is coding by 6 genes organized in operon, including two newly discovered members *PA14_16190* and *PA14_16200*, renamed *bacE* and *bacF*, respectively. By sequence analysis, we also proposed at the position −12 and −24 of *bacA* promoter region, a σ^54^-binding site (Figure 4B).

### 3.6 BacA is homologous to type-III secretion system chaperones and to regulatory proteins

**Figure 5:**
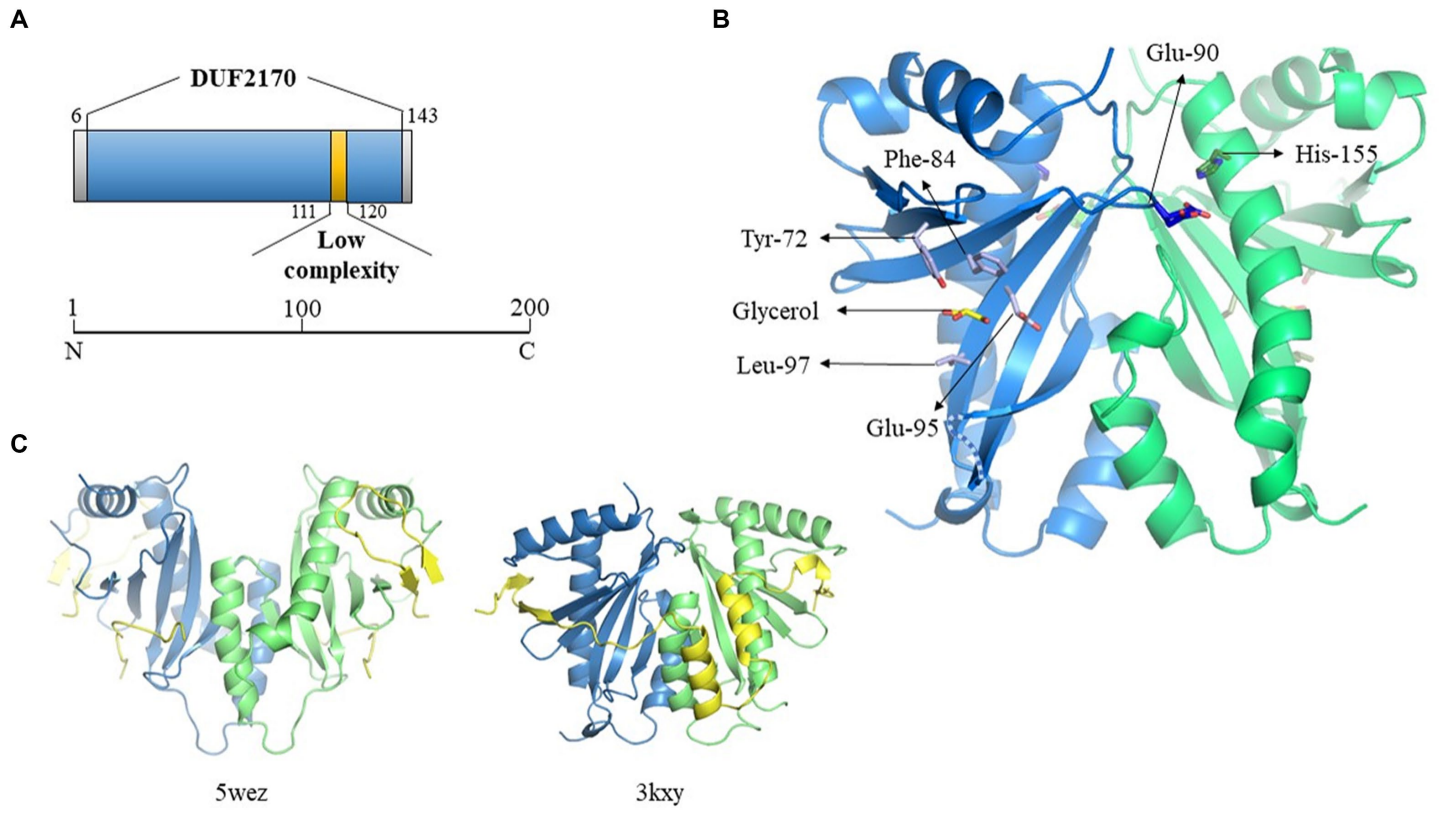
Crystallographic structure of BacA. **(A)** Schematic representation of the DUF2170 domain identified using Interpro database. **(B)** Structure of one of the two dimers of BacA, reconstructed after application of the crystallographic symmetries. The 3D structure is oriented in order to highlight the regions expected to interact with the polypeptide it is supposed to chaperon as solved in the structure of two other complexes involving a chaperon CesT/Tir (5wez) and ExsC/ExsE (3kxy) (see C where the interacting molecules are colored in yellow). The two monomers are colored in blue and green, respectively. One molecule of glycerol has been identified in the structure, localized in the pocket formed by four residues (Tyr-72, Phe-84, Glu-95, Leu-97) in the hollow of the concave beta sheet. The two residues solved in two alternative positions (Glu-90, His-155, see main text) are also indicated. The missing loop 130–136 is schematized as a dotted line. **(C)** Crystal structure of the Tir-CesT effector-chaperone complex (5wez, left part) and of the ExsC-ExsE complex (3kxy, right part). The PDB numbers and structures presented were obtained from the PDB website (https://www.rcsb.org/).

Further investigations to determine the function of BacA were initially addressed via bioinformatic analysis. The protein domains are presented in Figure 5A. The domain search performed with Interpro database^1^ revealed that BacA is identified as a DUF 2170-containing domain protein (Pfam PF09938) belonging to the YjfI family. The protein YjfI of *E. coli*, of unknown function, contains a DUF2170 domain and also a CesT/Tir-like domain. In *E. coli*, the protein chaperone of the T3SS CesT transports the translocated intimin receptor (Tir) through the T3SS (Elliott et al., 1999).

We then performed a search for proteins structurally homologous to BacA using the I-TASSER server (Yang and Zhang, 2015). The top 10 proteins are listed in the Supplementary Table S4 and their three-dimensional structure given in Supplementary Figure 4A. Here again, BacA appeared structurally similar to numerous T3SS chaperones/regulatory proteins. Despite a low sequence homology, these proteins share a common fold that is characterized by the same α-β-β-β-α-β-β-α topology. Among them, some are T3SS chaperones like SicP (1jyo) from *Salmonella enterica* which possesses the best TM-score (0.792), while others are regulators of gene expression, as YbjN (6vu7) from *E. coli* (TM-score of 0.732). The third protein in the list, named ExsC (3kxy), is both a T3SS chaperone and a transcription regulator (TM-score of 0.723) in *P. aeruginosa*.

However, a sequence homology search with the BacA amino acid sequence, directly targeting the protein data bank (PDB^2^), gave no result. This led us to explore its structure experimentally by crystallography. It was solved by using a caged terbium called Xo4 for experimental phasing, in space group P6_5_22 with two monomers in the asymmetric unit, in accordance with the size exclusion chromatography result. Nevertheless, the functional dimer conventionally described for the T3SS chaperone family does not correspond to this « asymmetric unit dimer » but is reconstituted by symmetry related molecules, issuing with two dimers each one being formed by crystallographic second order axis. The Tb-Xo4 molecule is localized at the interface of the two monomers present in the asymmetric unit, participating in the stabilization of the crystal packing, trapped by the N-terminus and Trp-39 of monomer A, by Glu-112 of monomer B interacting with the atom of terbium, and by Arg-136 of a symmetry-related monomer B (Supplementary Figure 4B).

The 3D structures of the two monomers are very similar, with a two-layers alpha + beta mixed domain, and adopt the α-β-β-β-α-β-β-α topology characteristic of the T3SS chaperone family, with an average root-mean-square deviation (RMSD) of 0.6 Å on Cα for the 115 closest residues over the 139 amino-acids that have been crystallized. All the variability is localized at the N- and C-terminus and in the three loops linking the beta strands on the surface of the protein at the opposite side of the 3 alpha helices. Only 8 residues for each monomer, 3 at the N-terminus, 2 at the C-terminus and 3 in the loop 130–136 have not been constructed due to the absence of electron density (see dotted lines Figure 5B). Two residues present alternative positions, Glu-90 in monomer A (A-Glu-90) and His-155 in monomer B (B-His-155), these residues being localized at the biological dimer interface, in close proximity (Figure 5B). In addition to the protein amino-acids, 29 water molecules have been added, which correspond to the expected number for this resolution, six of them being localized at the dimer’s interfaces. One molecule of glycerol has been trapped in monomer A in the concave surface of the twisted six-stranded anti-parallel β-sheet in a pocket formed by the side chains of 4 residues (Tyr-72, Phe-84, Glu-95, Leu97) (Figure 5B). The density present at the equivalent position in the second monomer is not clear enough to position a second glycerol molecule. The location where is found this glycerol in molecule A corresponds to the region of interaction between the chaperone and the effector in the structure solved of the complex formed between the multi-cargo chaperone CesT and Tir from *E. coli* (Little and Coombes, 2018), Tir being the first translocated effector in the infected host cell via the T3SS. Even if the different members of the T3SS chaperone family all adopt a similar 3D organization with 3 helices laying on a 5 to 6 strands β-sheet, it is very hard to superpose their respective structures, due to a very high flexibility of the structure render by the curvature of the β-sheet and the presence of the long loops between the different strands. This is especially true considering the respective orientation of the two molecules forming the functional dimer, even if the structure of BacA presents a high similitude with the one of ExsC, the protein from *P. aeruginosa* that presents both a chaperone and a transcription regulator function (Figure 5C).

## 4 Discussion

In the microbial world, biofilm formation is a widespread process during which bacteria move from a planktonic to an adherent state. Sessile bacteria then become particularly resistant to antimicrobials which causes major problems in public health (Wallart et al., 2023).

*P. aeruginosa* uses multiple appendages to explore and colonize its environment. This motility is a key feature during the bacterial infection. While flagellum-mediated swimming is adapted for movement in a liquid medium, coordinated multicellular migration, called swarming, is better suited for surface exploration. Surface properties such as hydrophobicity and charge have a significant influence on the bacterial adhesion. To compensate a lack of substratum attractiveness, *P. aeruginosa* can secrete biosurfactants among which are rhamnolipids (Banat et al., 2000; Ron and Rosenberg, 2001; Lang, 2002; Raza et al., 2010; Szymanska and Sadowski, 2010; Wu et al., 2019). The secretion of rhamnolipids is crucial to lower the surface tension, facilitating the migration of bacteria on the semi-solid surface as defined groups called tendrils (Kohler et al., 2000). The tendril movement is controlled by two secreted molecules with opposite action: the di-rhamnolipids attract cells while the rhamnolipids precursor 3-(3-hydroxyalkanoyloxy)alkanoic acids (HAAs) repels them (Tremblay et al., 2007; Bru et al., 2023; Kasallis et al., 2023). Here, the most abundant di-rhamnolipid Rha-Rha-C_10_-C_10_ was much less secreted by Δ*bacA* and this observation correlates with the reduced swarming migration observed. In addition, the irregularly shaped and slightly separated tendrils suggest that the mutant secretes less HAAs. Since HAAs are the precursor of rhamnolipids, this hypothesis could also explain the decrease in the secretion of mono- and di-rhamnolipids in the mutant. Indeed, the two rhamnosyltransferases RhlB and RhlC convert, respectively, HAAs into mono-rhamnolipids and mono-rhamnolipids into di-rhamnolipids by the addition of a dTDP-L-rhamnose (Ochsner et al., 1994; Rahim et al., 2001).

Beside their implication in swarming, rhamnolipids are known to be important determinants of biofilm formation, according to their localization and concentration within biofilms. Whereas lower concentrations promote cell aggregation and formation of microcolonies during starvation (Herman et al., 1997; Pamp and Tolker-Nielsen, 2007), high concentrations and swarming allow colonies to detach from mature biofilms to more favorable environments (Patriquin et al., 2008). In mature biofilms, to ensure transport of water and nutrients through channels, rhamnolipids prevent their colonization by inhibiting cell–cell interactions (Davey et al., 2003). Although the biofilm-defective phenotype of Δ*bacA* can be clearly linked to its deficiency in rhamnolipid secretion and so swarming motility, it remains difficult to understand the underlying mechanisms.

Therefore, we analyzed the proteomes of the WT and the Δ*bacA* grown in the biofilm mode. Interestingly, we observed a reduced abundance of the transcriptional regulator Vfr in the mutant (Figure 3C, colored in blue in the QS category), Vfr being able to activate LasR expression (Albus et al., 1997). The quorum sensing (QS) system Las, closely linked to the second QS system, Rhl, regulates several genes in a cell density dependent manner such as virulence factor production, biofilm maturation, rhamnolipid production and motility phenotypes. The QS system Rhl activates the expression of LecA and LecB, two lectins involved in adhesion to eukaryotic cells. In addition, the post-transcriptional regulator DksA, which was also less abundant in the mutant (Figure 3C, colored in blue in the oxidative stress category), is required for full translation of the *lasB* and *rhlAB* genes (Jude et al., 2003).

*P. aeruginosa* biofilms are constantly faced with reactive oxygen species (ROS) including the superoxide anion (O_2_^−^), hydrogen peroxide (H_2_O_2_), hypochlorous acid (HOCl), and the oxidizing hydroxyl radical (OḤ). They are endogenously generated by the reduction of oxygen in aerobic metabolism of bacterial cells (Seaver and Imlay, 2004) or released by the immune system cells during infection as a first line of defense (Sies et al., 2022). Among its broad arsenal, *P. aeruginosa* possesses several enzymes to detoxify oxidative agents. The Δ*bacA* exhibits a clear oxidative stress response failure as revealed by the decrease in a large proportion of detoxifying enzyme such as the two superoxide dismutases SodB and SodM (Figure 3C, colored in blue in the oxidative stress category) that catalyze the conversion of O_2_^−^ into H_2_O_2_ and three peroxidases that reduce H_2_O_2_ into H_2_O. To detoxify oxidative agents, all these enzymes utilize an immediate electron donor to the reduction reaction. Interestingly, we identified here, in the mutant, a lower abundance of peroxidase electron donor azurin Azu (Ronnberg et al., 1981; Wu et al., 2005) and thiol peroxidase electron donor thioredoxin TrxA (Jeong et al., 2000) (Figure 3C, colored in blue in the oxidative stress category). In *P. aeruginosa*, genes involved in the oxidative stress responses are tightly regulated by several transcriptional regulators. In the Δ*bacA*, the abundance level of the ferric uptake regulator Fur which positively regulates the expression of *sodB* gene was reduced. All these observations suggest that the Δ*bacA* may be more susceptible to oxidative agents leading to potential damage to proteins and lipids and emergence of mutations. Only the putative catalase KatE (also named KatC) appeared accumulated in the mutant, but its functional role remains unclear. Indeed, in PA14 strain, KatE was not shown to be involved in the H_2_O_2_ detoxification (Lee et al., 2005) while it appeared to protect the PAO6049 strain in a temperature dependant manner (Mossialos et al., 2006).

The present study shows that the BAC system is formed by the 6 genes *bacABCDEF* operon (from *PA14_16140* to *PA14_16200*), where *bacE* and *bacF* are here newly identified. Additionally, the proteomic analysis provided evidence that accumulation of BAC cognate partners was correlated with the absence of BacA (Figure 3C, colored in red in the Unknown category). Indeed, the shared fold between BacA and T3SS chaperones, known to possess post-transcriptional regulation activity in addition to the one of chaperoning, suggests that BacA could be part of a regulatory pathway conducting through specific protein–protein interactions necessary for the transcription of the *bac* operon. This hypothesis is supported by the BacA dimer interactions detected in the crystal packing which are reminiscent of the way that T3SS chaperones interact with their respective targets. Moreover, the String protein–protein network suggests interactions between all the BAC proteins, that were already observed during additional works pointing out a strong interaction between BacA and BacB (unpublished results; Ben Mlouka, 2014). A thorough experimental analysis of the regulatory interaction of BacA with its cognate partners will be essential to confirm our hypothesis.

In addition, structural homology search with i-TASSER showed conserved domain architecture between BacA and members of the YbjN superfamily (*E. amylovora* AmyR, *D. radiodurans* DR1245, *E. coli* YbjN and *S. elongates* T110839). The latter are involved in the regulatory pathways of the stress-response (Hayashi et al., 2003; Bore et al., 2007; Keeney et al., 2008; Wang et al., 2011; Norais et al., 2013; Byrne et al., 2014; Piqué et al., 2015) which enhances our hypothesis.

BAC system might be an original Psp system, a well-known stress-response system. Since the discovery of the historical *E. coli* Psp system, numerous Psp systems were found in photosynthetic eukaryotes, archaea (Westphal et al., 2001; Bultema et al., 2010; Vothknecht et al., 2012; Heidrich et al., 2017) and bacteria including the most studied *pspABCDE* of *E. coli* (Proteobacteria), *liaIHGFSR* of *B. subtilis* (Firmicutes) and *clgRpspAMN* of *M. tuberculosis* (Actinobacteria). These studies highlighted that *E. coli* Psp system is not universal and the occurrence of variable genomic contexts around a *pspA* gene, the only one conserved. However, it remains unclear how the different Psp proteins have evolved and how the variable genomic contexts have emerged. To address these questions, current studies focused on the Psp systems phyletic spreads across the bacterial (Ravi et al., 2018, 2023; Popp et al., 2022).

First, the PspA protein and all its homologs, contains a conserved PspA_IM30 domain that has a predominantly coiled-coil structure (Ranea et al., 2006; Jovanovic et al., 2014; Heidrich et al., 2016; McDonald et al., 2017; Junglas et al., 2020). Interestingly, the domain PspA_IM30 is also contained by BacB (accession A0A0H2ZES6 on UniProt database^3^) and its structure exclusively helicoidal promotes the formation of coiled-coil structure (Macé et al., 2008). Secondly, based on the BacA bioinformatic analysis, BacA belongs to the DUF2170-containing YjfI family. In *E. coli*, the gene *yjfI* is part of the *yjfIJ* operon where YjfI has no attributed function and YjfJ is a PspA homolog (Pederick et al., 2023). Little is known about the DUF2170-containing proteins. In the literature, these proteins are exclusively cited in phylogenetic studies (Ravi et al., 2018, 2023; Popp et al., 2022). Thus, by comparing the *bac* operon with the genomic contexts described by Ravi et al. (2023), two genomic contexts contained a DUF2170-protein and are exclusively present in Proteobacteria. In the first one, a DUF2170-containing protein and a PspA homolog are associated with a membrane-associated spermidine synthase. However, no protein in or around the *bac* operon exhibits a sequence homology with a spermidine synthase. Moreover, spermidine synthesis is well documented in *P. aeruginosa* and genes involved in this pathway are identified (Lu et al., 2002; Nakada and Itoh, 2003). The second one is described as an operon of 6 genes, including a DUF2170-containing protein in first position and a PspA homolog just downstream. Unfortunately, no experimental data is yet available for this operon. To our knowledge, the existence of a Psp system in *P. aeruginosa,* has never been reported until yet. It’s clear that further genomic and functional comparative analyses between these operons are required to elucidate the precise functions of all the BAC proteins, in particular in the biofilm lifestyle.

## 5 Conclusion

We show here that the BAC system is coded by 6 genes (*bacA* to *bacF*) organized in operon. As for *E. coli psp* regulon, the *bacABCDEF* operon transcription appears dependent on the σ^54^ sigma factor. However, the *P. aeruginosa* Bac genomic context is highly different from that of Psp in *E. coli*. The BAC system is obviously involved in different mechanisms linked to virulence and biofilm formation in *P. aeruginosa*. We hope that these data will pave the route to numerous works aiming at an in-depth characterization of the contribution of the different actors of this system.

## Data availability statement

The datasets presented in this study can be found in online repositories. The names of the repository/repositories and accession number(s) can be found here: http://www.proteomexchange.org/, PXD044736; http://www.wwpdb.org/, 8Q8O.

## Author contributions

LW: Conceptualization, Formal analysis, Investigation, Methodology, Software, Writing – original draft, Writing – review & editing. MB: Formal analysis, Investigation, Methodology, Writing – review & editing. BS: Investigation, Methodology, Writing – review & editing. LC: Investigation, Methodology, Writing – review & editing. HL: Investigation, Methodology, Writing – review & editing. JH: Investigation, Methodology, Writing – review & editing. TJ: Conceptualization, Supervision, Writing – original draft, Writing – review & editing. GP: Writing – review & editing. M-CK-M: Investigation, Methodology, Writing – review & editing. EG: Investigation, Methodology, Writing – review & editing. IB: Investigation, Methodology, Software, Supervision, Writing – review & editing. PC: Conceptualization, Methodology, Resources, Supervision, Validation, Writing – original draft, Writing – review & editing.

## Footnotes

^1^ https://www.ebi.ac.uk/interpro/

^2^ https://www.rcsb.org/

^3^ https://www.uniprot.org/
