# Supplementary material for: BacA: a possible regulator that contributes to the biofilm formation of Pseudomonas aeruginosa
Source: Front Microbiol. 2024 Mar 5;15:1332448. doi: 10.3389/fmicb.2024.1332448 (PMC10948618; doi:10.3389/fmicb.2024.1332448)
Supplement: Supplementary file 1 [file Table_1.pdf]

Table S1. Primers used in this study.

Primer name	Sequence (5’>3’)	Amplicon size (bp)
**PCR**		
PA14_WT_F	GCTCATTTGGAGAAGGAGGAAGAC	478
PA14_WT_R	TTTCAGGTAGCCGGCGTAGACTT	
PA14_Δ*16140*_F	GCTCATTTGGAGAAGGAGGAAGAC	1472
PA14_Δ*16140*_R	TTTCAGGTAGCCGGCGTAGACTT	
**RT-PCR**		
PA14_*16140/50*_F	TTCAACGAAGAGGTCCTGCT	233
PA14_*16140/50*_R	AGCTTGCTCCATACCGTCAT	
PA14_*16150/60*_F	TACGGCAACTCGAAGAGCAA	497
PA14_*16150/60*_R	ACGCCGTTGAGTTTCAGCTT	
PA14_*16160/80*_F	CCGGGCTGATCCTGAAAGTT	635
PA14_*16160/80*_R	GATCACCTCGATGATGCGGT	
PA14_*16180/90*_F	CGAGGGCGACTTCTTCAACT	557
PA14_*16180/90*_R	GCGATGCAGTTGTTTTCGGT	
PA14_*16190/20*_F	TGATCAAGGTGATGGACCGC	501
PA14_*16190/20*_R	GTCCAATCCGTCGAAGTCCA	
