# Supplementary material for: BacA: a possible regulator that contributes to the biofilm formation of Pseudomonas aeruginosa
Source: Front Microbiol. 2024 Mar 5;15:1332448. doi: 10.3389/fmicb.2024.1332448 (PMC10948618; doi:10.3389/fmicb.2024.1332448)
Supplement: Supplementary file 2 [file Table_2.pdf]

Table S2: Data recording and refinements statistics for the dataset recorded on the PX1 beamline at the synchrotron SOLEIL. (Data from the highest resolution bin are in parentheses).

**Data collection and integration statistics**	**BacA-**TB-XO4
Wavelength (Å)	1.649
space group	P6(5)22
Cell parameters (Å) a = b; c	68.86; 281.07
Matthews coefficient (Å3/Da)	2.93
Percentage of solvent (%)	58.0
Resolution limits (Å)	46.85 - 2.7 Å (2.83-2.70)
No of measured reflections	434678 (56933)
No of unique reflections	11686 (1478)
Rmerge (%)[a]	16.9 (109.9)
Rpim (%)	3.7 (24.3)
CC1/2 (%)	99.9 (95.4)
Completeness (%)	99.70 (99.3)
Redundancy	21.40 (21.10)
I/σ(I)	22.9 (5.0)
Anomalous measurability limit (Å)	2.82
Figure of merit	0.829
Phase error (°)	32.27
B Wilson (Å^2)	40.66
**Refinement statistics by resolution**	46.85 - 2.7 Å
Rwork/Rfree (% / %)	21.03/26.88
No of used reflections	11472
No of refined atoms	2199
No of amino-acids	276
No of water molecules	27
No of alternates positions	2
No of additives	2
rms bonds deviation (Å)	0.011
rms angle deviation (°)	0.575
Average thermal B factors (Å^2)	46.96
Ligands thermal B factors (Å^2)	75.54
Ramachandran Favored (%)	95.52
Ramachandran Outliers (%)	0.75

(a) Rmerge is the standard agreement factor = ∑hkl∑j (|Ihkl,j−<Ihkl>|) / ∑hkl∑j (Ihkl,j), and Rpim is the precision-indicating merging R-factor = ∑Hp(1/(n-1)∑j (|IH,j−<IH>|) / ∑H∑j (IH,j). The Rmerge was always rather high but trusting on the Rpim was always the guideline for data quality.
