# Supplementary material for: BacA: a possible regulator that contributes to the biofilm formation of Pseudomonas aeruginosa
Source: Front Microbiol. 2024 Mar 5;15:1332448. doi: 10.3389/fmicb.2024.1332448 (PMC10948618; doi:10.3389/fmicb.2024.1332448)
Supplement: Supplementary file 3 [file Table_3.pdf]

<u>Table S3</u>: Proteins identified in biofilm formed by the WT and *bacA* mutant

Accession	Unique peptides	Confidence score	Anova (p)	q Value	Max fold change	Power	Highest mean condition	Lowest mean condition	Gene	Description
YP_788156.1|gi|116053721|	13	612	6,12E-01	4,80E-01	1,04	0,07	WT	bacA mutant	PA14_00010	chromosomal replication initiation protein
YP_788157.1|gi|116053722|	16	1188	8,04E-03	5,98E-02	1,34	0,95	WT	bacA mutant	PA14_00020	DNA polymerase III subunit beta
YP_788158.1|gi|116053723|	3	107	5,18E-01	4,43E-01	1,05	0,09	bacA mutant	WT	PA14_00030	recombination protein F
YP_788159.1|gi|116053724|	14	720	8,09E-01	5,44E-01	1,04	0,05	WT	bacA mutant	PA14_00050	DNA gyrase subunit B
YP_788163.1|gi|116053728|	13	817	2,49E-01	3,00E-01	1,16	0,18	WT	bacA mutant	PA14_00090	glycyl-tRNA synthetase subunit beta
YP_788164.1|gi|116053729|	8	361	7,33E-01	5,23E-01	1,09	0,06	WT	bacA mutant	PA14_00100	glycyl-tRNA synthetase subunit alpha
YP_788166.1|gi|116053731|	5	164	9,55E-01	5,72E-01	1,01	0,05	WT	bacA mutant	PA14_00120	lipid A biosynthesis lauroyl acyltransferase
YP_788171.1|gi|116053736|	5	220	8,60E-01	5,53E-01	1,02	0,05	WT	bacA mutant	PA14_00170	potassium transporter peripheral membrane protein
YP_788172.1|gi|116053737|	4	266	4,23E-01	4,00E-01	1,17	0,11	bacA mutant	WT	PA14_00180	tRNA and rRNA cytosine-C5-methylases
YP_788173.1|gi|116053738|	2	94	5,65E-01	4,60E-01	1,10	0,08	WT	bacA mutant	PA14_00190	methionyl-tRNA formyltransferase
YP_788175.1|gi|116053740|	10	529	2,55E-01	3,05E-01	1,12	0,18	WT	bacA mutant	PA14_00210	lysin domain-containing protein
YP_788178.1|gi|116053743|	14	950	1,92E-01	2,54E-01	1,27	0,23	WT	bacA mutant	PA14_00250	quinone oxidoreductase
YP_788179.1|gi|116053744|	2	73	4,35E-01	4,07E-01	1,07	0,10	bacA mutant	WT	PA14_00280	coproporphyrinogen III oxidase
YP_788191.1|gi|116053756|	3	127	8,20E-02	1,58E-01	1,56	0,42	WT	bacA mutant	PA14_00440	tryptophan synthase subunit alpha
YP_788192.1|gi|116053757|	7	242	1,25E-02	6,84E-02	1,27	0,89	WT	bacA mutant	PA14_00450	tryptophan synthase subunit beta
YP_788194.1|gi|116053759|	4	339	7,68E-01	5,34E-01	1,02	0,06	WT	bacA mutant	PA14_00470	hypothetical protein
YP_788196.1|gi|116053761|	7	317	1,08E-02	6,67E-02	1,92	0,91	bacA mutant	WT	PA14_00490	hemolysin activation/secretion protein
YP_788208.1|gi|116053773|	10	636	9,52E-01	5,72E-01	1,03	0,05	WT	bacA mutant	PA14_00640	potential phenazine-modifying enzyme
YP_788211.1|gi|116053776|	2	92	3,22E-03	4,73E-02	1,66	0,99	WT	bacA mutant	PA14_00670	hypothetical protein
YP_788215.1|gi|116053780|	5	285	6,84E-02	1,44E-01	1,88	0,47	WT	bacA mutant	PA14_00710	osmotically inducible protein OsmC
YP_788222.1|gi|116053787|	2	94	3,86E-02	1,12E-01	1,53	0,63	WT	bacA mutant	PA14_00780	hypothetical protein
YP_788223.1|gi|116053788|	9	458	6,32E-01	4,86E-01	1,08	0,07	WT	bacA mutant	PA14_00790	oligopeptidase A
YP_788226.1|gi|116053791|	10	1003	7,30E-01	5,22E-01	1,10	0,06	bacA mutant	WT	PA14_00820	hypothetical protein

YP_788235.1|gi|116053798|	3	139	2,50E-01	3,01E-01	1,30	0,18	bacA mutant	WT	PA14_00940	hypothetical protein
YP_788240.1|gi|116053803|	2	168	6,05E-02	1,35E-01	1,24	0,50	WT	bacA mutant	PA14_01010	hypothetical protein
YP_788241.1|gi|116053804|	11	645	4,36E-01	4,07E-01	1,13	0,10	WT	bacA mutant	PA14_01020	hypothetical protein
YP_788242.1|gi|116053805|	3	168	1,21E-01	1,98E-01	1,37	0,33	WT	bacA mutant	PA14_01030	hypothetical protein
YP_788243.1|gi|116053806|	2	98	1,09E-04	2,39E-02	2,30	1,00	bacA mutant	WT	PA14_01040	secretion protein
YP_788260.1|gi|116053823|	5	372	3,45E-02	1,06E-01	1,20	0,66	WT	bacA mutant	PA14_01240	carbonic anhydrase
YP_788273.1|gi|116053836|	2	52	8,24E-01	5,48E-01	1,04	0,05	WT	bacA mutant	PA14_01400	hypothetical protein
YP_788278.1|gi|116053841|	4	134	7,63E-01	5,32E-01	1,03	0,06	WT	bacA mutant	PA14_01470	transcriptional regulator
YP_788280.1|gi|116053843|	8	764	7,92E-02	1,56E-01	1,81	0,43	WT	bacA mutant	PA14_01490	hemolysin
YP_788288.1|gi|116053851|	10	548	4,40E-03	4,75E-02	1,35	0,99	bacA mutant	WT	PA14_01600	aldehyde dehydrogenase
YP_788290.1|gi|116053853|	8	410	1,60E-01	2,27E-01	1,26	0,27	bacA mutant	WT	PA14_01620	beta alanine--pyruvate transaminase
YP_788296.1|gi|116053859|	12	767	1,13E-02	6,67E-02	1,50	0,91	WT	bacA mutant	PA14_01710	alkyl hydroperoxide reductase
YP_788297.1|gi|116053860|	11	506	1,99E-02	8,67E-02	1,42	0,80	bacA mutant	WT	PA14_01720	alkyl hydroperoxide reductase
YP_788298.1|gi|116053861|	18	920	1,64E-02	7,76E-02	1,66	0,84	bacA mutant	WT	PA14_01730	hypothetical protein
YP_788300.1|gi|116053863|	10	641	1,06E-02	6,67E-02	1,41	0,91	WT	bacA mutant	PA14_01760	nonspecific ribonucleoside hydrolase
YP_788302.1|gi|116053865|	4	159	1,77E-02	8,05E-02	1,40	0,82	WT	bacA mutant	PA14_01780	nucleoside 2-deoxyribosyltransferase
YP_788306.1|gi|116053869|	2	93	5,92E-01	4,70E-01	1,08	0,07	bacA mutant	WT	PA14_01830	adenosine deaminase
YP_788313.1|gi|116053876|	2	47	2,24E-01	2,81E-01	1,21	0,20	WT	bacA mutant	PA14_01930	transcriptional regulator PcaR
YP_788314.1|gi|116053877|	7	516	7,12E-01	5,18E-01	1,04	0,06	WT	bacA mutant	PA14_01940	RND efflux membrane fusion protein
YP_788315.1|gi|116053878|	11	628	8,53E-01	5,53E-01	1,01	0,05	WT	bacA mutant	PA14_01960	RND efflux membrane fusion protein
YP_788316.1|gi|116053879|	4	164	7,94E-01	5,40E-01	1,21	0,06	WT	bacA mutant	PA14_01970	RND efflux transporter
YP_788317.1|gi|116053880|	2	92	8,91E-01	5,58E-01	1,01	0,05	bacA mutant	WT	PA14_01980	LysR family transcriptional regulator
YP_788320.1|gi|116053883|	3	247	2,92E-02	9,96E-02	1,84	0,70	bacA mutant	WT	PA14_02020	outer membrane porin
YP_788334.1|gi|116053897|	7	330	2,54E-01	3,04E-01	1,32	0,18	WT	bacA mutant	PA14_02220	chemotaxis transducer
YP_788351.1|gi|116053913|	4	219	4,16E-02	1,16E-01	1,53	0,61	WT	bacA mutant	PA14_02450	NAD(P) transhydrogenase subunit alpha part 1
YP_788381.1|gi|116053943|	3	84	4,86E-01	4,29E-01	1,15	0,09	WT	bacA mutant	PA14_02830	3-carboxy-cis,cis-muconate cycloisomerase

YP_788382.1|gi|116053944|	3	202	4,49E-01	4,13E-01	1,05	0,10	WT	bacA mutant	PA14_02840	beta-ketoadipate enol-lactone hydrolase
YP_788387.1|gi|116053949|	5	308	3,08E-01	3,41E-01	1,14	0,15	WT	bacA mutant	PA14_02910	IclR family transcriptional regulator
YP_788401.1|gi|116053963|	3	246	1,13E-02	6,67E-02	1,70	0,91	WT	bacA mutant	PA14_03090	hypothetical protein
YP_788408.1|gi|116054432|	2	78	1,55E-01	2,25E-01	1,19	0,27	bacA mutant	WT	PA14_03163	hypothetical protein
YP_788412.1|gi|116053972|	3	130	3,07E-01	3,41E-01	1,53	0,15	bacA mutant	WT	PA14_03190	hypothetical protein
YP_788416.1|gi|116053976|	5	398	3,53E-01	3,64E-01	1,12	0,13	WT	bacA mutant	PA14_03240	secreted protein Hcp
YP_788418.1|gi|116054434|	2	51	2,32E-01	2,88E-01	1,20	0,19	WT	bacA mutant	PA14_03265	hypothetical protein
YP_788427.1|gi|116053985|	2	93	6,00E-01	4,73E-01	1,10	0,07	bacA mutant	WT	PA14_03350	hypothetical protein
YP_788428.1|gi|116053986|	3	136	1,33E-01	2,09E-01	1,33	0,31	bacA mutant	WT	PA14_03360	hypothetical protein
YP_788429.1|gi|116053987|	2	95	1,00E-01	1,80E-01	1,37	0,37	bacA mutant	WT	PA14_03370	hypothetical protein
YP_788434.1|gi|116053992|	22	1743	1,80E-02	8,08E-02	1,25	0,82	WT	bacA mutant	PA14_03430	succinate-semialdehyde dehydrogenase I
YP_788435.1|gi|116053993|	22	1554	7,89E-01	5,39E-01	1,02	0,06	bacA mutant	WT	PA14_03450	4-aminobutyrate aminotransferase
YP_788437.1|gi|116053995|	2	77	7,78E-01	5,38E-01	1,05	0,06	WT	bacA mutant	PA14_03480	GntR family transcriptional regulator
YP_788438.1|gi|116053996|	7	518	1,18E-02	6,76E-02	1,52	0,90	WT	bacA mutant	PA14_03490	hypothetical protein
YP_788449.1|gi|116054007|	2	153	4,36E-01	4,07E-01	1,07	0,10	bacA mutant	WT	PA14_03650	sulfate transport protein CysA
YP_788460.1|gi|116054018|	19	1402	9,04E-01	5,59E-01	1,00	0,05	WT	bacA mutant	PA14_03800	anaerobically-induced outer membrane porin OprE precursor
YP_788461.1|gi|116054019|	7	367	1,05E-02	6,67E-02	1,42	0,92	WT	bacA mutant	PA14_03810	agmatine deiminase
YP_788464.1|gi|116054403|	2	83	7,34E-01	5,23E-01	1,02	0,06	bacA mutant	WT	PA14_03855	periplasmic polyamine binding protein
YP_788465.1|gi|116054022|	9	567	4,60E-01	4,17E-01	1,22	0,10	WT	bacA mutant	PA14_03860	glutamine synthetase
YP_788467.1|gi|116054024|	5	273	8,85E-01	5,56E-01	1,01	0,05	WT	bacA mutant	PA14_03880	glutamine synthetase
YP_788468.1|gi|116054025|	17	968	5,13E-02	1,23E-01	1,13	0,55	WT	bacA mutant	PA14_03900	aminotransferase
YP_788469.1|gi|116054026|	11	584	6,44E-04	2,67E-02	1,88	1,00	WT	bacA mutant	PA14_03920	polyamine transport protein
YP_788470.1|gi|116054027|	9	334	2,16E-03	4,01E-02	1,71	1,00	WT	bacA mutant	PA14_03930	polyamine transport protein
YP_788471.1|gi|116054028|	6	418	2,74E-02	9,79E-02	1,75	0,72	WT	bacA mutant	PA14_03940	polyamine transport protein PotG
YP_788472.1|gi|116054029|	3	107	8,26E-01	5,48E-01	1,46	0,05	WT	bacA mutant	PA14_03950	polyamine transport protein PotH
YP_788476.1|gi|116054033|	8	820	4,46E-01	4,12E-01	1,04	0,10	WT	bacA mutant	PA14_04010	hypothetical protein

YP_788479.1|gi|116054036|	3	148	5,82E-01	4,66E-01	1,12	0,08	WT	bacA mutant	PA14_04040	hypothetical protein
YP_788484.1|gi|116054041|	2	104	8,56E-01	5,53E-01	1,10	0,05	bacA mutant	WT	PA14_04090	ABC transporter substrate-binding protein
YP_788485.1|gi|116054042|	5	276	1,19E-02	6,82E-02	3,12	0,90	WT	bacA mutant	PA14_04100	hypothetical protein
YP_788486.1|gi|116054043|	9	483	2,06E-01	2,67E-01	1,12	0,22	WT	bacA mutant	PA14_04110	D-3-phosphoglycerate dehydrogenase
YP_788487.1|gi|116054044|	8	301	2,55E-01	3,05E-01	1,18	0,18	WT	bacA mutant	PA14_04140	hypothetical protein
YP_788488.1|gi|116054045|	3	186	5,41E-02	1,28E-01	1,40	0,54	WT	bacA mutant	PA14_04150	hypothetical protein
YP_788499.1|gi|116054056|	5	389	1,24E-01	1,99E-01	1,76	0,32	WT	bacA mutant	PA14_04300	hypothetical protein
YP_788500.1|gi|116054057|	3	160	7,30E-01	5,22E-01	1,06	0,06	WT	bacA mutant	PA14_04310	ribose-5-phosphate isomerase A
YP_788501.1|gi|116054058|	8	325	3,85E-01	3,80E-01	1,07	0,12	WT	bacA mutant	PA14_04320	threonine dehydratase
YP_788506.1|gi|116054063|	2	47	2,91E-02	9,95E-02	1,36	0,71	bacA mutant	WT	PA14_04380	hypothetical protein
YP_788508.1|gi|116054065|	4	222	2,26E-01	2,83E-01	1,33	0,20	WT	bacA mutant	PA14_04410	phosphoenolpyruvate-protein phosphotransferase PtsP
YP_788513.1|gi|116054070|	3	134	7,57E-01	5,29E-01	1,06	0,06	WT	bacA mutant	PA14_04480	thymidylate synthase
YP_788515.1|gi|116054072|	2	98	3,03E-02	1,01E-01	1,40	0,69	bacA mutant	WT	PA14_04510	hypothetical protein
YP_788516.1|gi|116054073|	2	62	8,02E-01	5,42E-01	1,01	0,06	WT	bacA mutant	PA14_04520	hypothetical protein
YP_788524.1|gi|116054081|	9	530	7,44E-01	5,27E-01	1,03	0,06	bacA mutant	WT	PA14_04630	dihydroxy-acid dehydratase
YP_788526.1|gi|116054083|	5	220	4,01E-01	3,87E-01	1,07	0,11	WT	bacA mutant	PA14_04650	protease PfpI
YP_788528.1|gi|116054085|	2	67	3,67E-01	3,71E-01	1,15	0,12	WT	bacA mutant	PA14_04670	formamidopyrimidine-DNA glycosylase
YP_788533.1|gi|116054090|	2	95	3,33E-01	3,54E-01	1,27	0,14	WT	bacA mutant	PA14_04730	gamma-glutamyltranspeptidase
YP_788535.1|gi|116054092|	5	283	7,28E-03	5,85E-02	1,58	0,96	WT	bacA mutant	PA14_04760	phosphopantetheine adenylyltransferase
YP_788538.1|gi|116054095|	14	761	8,71E-01	5,55E-01	1,03	0,05	WT	bacA mutant	PA14_04810	aldehyde dehydrogenase
YP_788543.1|gi|116054100|	2	45	1,24E-01	1,99E-01	1,16	0,32	WT	bacA mutant	PA14_04860	methyltransferase
YP_788544.1|gi|116054101|	8	343	1,10E-02	6,67E-02	1,45	0,91	WT	bacA mutant	PA14_04870	hypothetical protein
YP_788545.1|gi|116054102|	12	769	2,18E-03	4,01E-02	1,21	1,00	WT	bacA mutant	PA14_04890	zinc protease
YP_788546.1|gi|116054103|	8	451	1,29E-01	2,05E-01	1,13	0,31	WT	bacA mutant	PA14_04900	signal recognition particle receptor FtsY
YP_788547.1|gi|116054104|	6	291	2,55E-01	3,04E-01	1,23	0,18	WT	bacA mutant	PA14_04910	cell division ATP-binding protein FtsE
YP_788548.1|gi|116054105|	2	50	6,35E-01	4,88E-01	1,52	0,07	WT	bacA mutant	PA14_04920	cell division protein FtsX

YP_788554.1|gi|116054111|	3	227	1,59E-02	7,69E-02	1,61	0,85	WT	bacA mutant	PA14_04980	thiazole synthase
YP_788555.1|gi|116054112|	2	53	1,70E-01	2,35E-01	1,15	0,25	bacA mutant	WT	PA14_05000	tRNA (guanine-N(7)-)-methyltransferase
YP_788560.1|gi|116054117|	4	198	5,87E-01	4,68E-01	1,18	0,07	bacA mutant	WT	PA14_05050	deoxyribonucleotide triphosphate pyrophosphatase
YP_788561.1|gi|116054118|	4	183	4,37E-02	1,17E-01	1,63	0,59	WT	bacA mutant	PA14_05060	hypothetical protein
YP_788567.1|gi|116054124|	5	370	5,93E-02	1,34E-01	1,32	0,51	WT	bacA mutant	PA14_05150	pyrroline-5-carboxylate reductase
YP_788570.1|gi|116054127|	6	299	2,20E-01	2,78E-01	1,15	0,20	WT	bacA mutant	PA14_05190	twitching motility protein PilU
YP_788573.1|gi|116054130|	23	1854	7,32E-03	5,85E-02	1,31	0,96	bacA mutant	WT	PA14_05220	cystathionine beta-synthase
YP_788574.1|gi|116054131|	12	840	7,67E-03	5,89E-02	1,30	0,95	WT	bacA mutant	PA14_05230	cystathionine gamma-lyase
YP_788575.1|gi|116054132|	7	381	6,70E-01	5,03E-01	1,09	0,07	WT	bacA mutant	PA14_05250	dihydroorotase
YP_788576.1|gi|116054133|	5	364	9,66E-01	5,75E-01	1,01	0,05	WT	bacA mutant	PA14_05260	aspartate carbamoyltransferase
YP_788577.1|gi|116054134|	2	128	5,08E-02	1,23E-01	1,49	0,55	WT	bacA mutant	PA14_05270	bifunctional pyrimidine regulatory protein PyrR/uracil phosphoribosyltransferase
YP_788581.1|gi|116054138|	10	578	5,05E-03	4,82E-02	1,32	0,98	WT	bacA mutant	PA14_05310	glutathione synthetase
YP_788582.1|gi|116054139|	3	93	2,32E-01	2,87E-01	1,29	0,19	WT	bacA mutant	PA14_05320	twitching motility protein PilG
YP_788585.1|gi|116054142|	8	726	4,91E-01	4,31E-01	1,42	0,09	WT	bacA mutant	PA14_05360	twitching motility protein PilJ
YP_788587.1|gi|116054144|	6	179	9,24E-01	5,65E-01	1,08	0,05	WT	bacA mutant	PA14_05390	ChpA
YP_788589.1|gi|116054146|	3	138	7,86E-01	5,39E-01	1,04	0,06	WT	bacA mutant	PA14_05410	chemotaxis protein
YP_788592.1|gi|116054149|	2	46	3,57E-01	3,66E-01	1,13	0,13	bacA mutant	WT	PA14_05440	hypothetical protein
YP_788594.1|gi|116054151|; YP_794010.1|gi|116053683|	3	136	7,88E-01	5,39E-01	1,02	0,06	bacA mutant	WT	PA14_05460	adenosylmethionine-8-amino-7-oxononanoate aminotransferase
YP_788595.1|gi|116054152|	6	222	9,43E-01	5,71E-01	1,01	0,05	bacA mutant	WT	PA14_05480	hypothetical protein
YP_788597.1|gi|116054154|	12	982	6,28E-02	1,37E-01	1,67	0,49	WT	bacA mutant	PA14_05510	hypothetical protein
YP_788599.1|gi|116054156|	16	1327	3,34E-01	3,54E-01	1,14	0,14	WT	bacA mutant	PA14_05530	RND multidrug efflux membrane fusion protein MexA
YP_788600.1|gi|116054157|; YP_790743.1|gi|116050438|	26	1553	9,42E-01	5,71E-01	1,16	0,05	WT	bacA mutant	PA14_05540	RND multidrug efflux transporter MexB
YP_788601.1|gi|116054158|	26	2457	8,77E-01	5,55E-01	1,07	0,05	bacA mutant	WT	PA14_05550	major intrinsic multiple antibiotic resistance efflux outer membrane protein OprM precursor
YP_788602.1|gi|116054159|	13	693	5,67E-01	4,60E-01	1,18	0,08	bacA mutant	WT	PA14_05560	ATP-dependent RNA helicase
YP_788603.1|gi|116054160|	3	209	5,89E-02	1,34E-01	1,48	0,51	bacA mutant	WT	PA14_05580	hypothetical protein

YP_788604.1|gi|116054161|	8	532	2,51E-01	3,02E-01	1,14	0,18	WT	bacA mutant	PA14_05590	5,10-methylenetetrahydrofolate reductase
YP_788606.1|gi|116054163|	24	1593	1,59E-01	2,27E-01	1,29	0,27	WT	bacA mutant	PA14_05620	S-adenosyl-L-homocysteine hydrolase
YP_788611.1|gi|116054168|	7	341	8,79E-01	5,55E-01	1,04	0,05	bacA mutant	WT	PA14_05690	cytosine deaminase
YP_788619.1|gi|116054175|	11	613	1,31E-02	6,92E-02	1,15	0,88	bacA mutant	WT	PA14_05820	hypothetical protein
YP_788620.1|gi|116054176|	20	1445	4,75E-02	1,20E-01	1,19	0,57	bacA mutant	WT	PA14_05840	glutaryl-CoA dehydrogenase
YP_788622.1|gi|116054178|	5	334	3,46E-01	3,60E-01	1,29	0,13	WT	bacA mutant	PA14_05860	hypothetical protein
YP_788628.1|gi|116054184|	4	100	1,38E-01	2,13E-01	1,26	0,30	WT	bacA mutant	PA14_05950	ATP-dependent RNA helicase DbpA
YP_788629.1|gi|116054185|	2	100	8,66E-01	5,54E-01	1,39	0,05	bacA mutant	WT	PA14_05960	cold-shock protein
YP_788632.1|gi|116054188|	33	1779	4,10E-02	1,16E-01	1,66	0,61	WT	bacA mutant	PA14_06000	ClpA/B protease ATP binding subunit
YP_788633.1|gi|116054189|	6	433	7,35E-02	1,49E-01	2,16	0,45	WT	bacA mutant	PA14_06010	hypothetical protein
YP_788635.1|gi|116054190|	4	157	1,17E-01	1,95E-01	1,53	0,33	WT	bacA mutant	PA14_06040	hypothetical protein
YP_788641.1|gi|116054197|	2	83	2,31E-01	2,87E-01	1,64	0,19	bacA mutant	WT	PA14_06130	hypothetical protein
YP_788642.1|gi|116054198|	13	710	5,64E-02	1,31E-01	1,07	0,52	WT	bacA mutant	PA14_06150	hypothetical protein
YP_788646.1|gi|116054202|	4	118	2,28E-02	9,26E-02	1,67	0,77	WT	bacA mutant	PA14_06190	glutathione S-transferase
YP_788652.1|gi|116054208|	2	83	7,49E-02	1,49E-01	1,13	0,45	WT	bacA mutant	PA14_06260	LysR family transcriptional regulator
YP_788653.1|gi|116054209|	3	273	2,00E-01	2,61E-01	1,31	0,22	WT	bacA mutant	PA14_06270	hydrolase
YP_788655.1|gi|116054211|	21	1344	1,39E-01	2,14E-01	1,34	0,29	WT	bacA mutant	PA14_06290	malate synthase G
YP_788659.1|gi|116054215|	5	266	2,04E-01	2,65E-01	1,14	0,22	WT	bacA mutant	PA14_06330	serine/threonine protein kinase
YP_788672.1|gi|116054228|	2	90	2,83E-01	3,26E-01	1,15	0,16	bacA mutant	WT	PA14_06530	biotin biosynthesis protein bioH
YP_788676.1|gi|116054232|; YP_788677.1|gi|116054233|; YP_791961.1|gi|116049236|	24	1585	7,02E-02	1,44E-01	1,32	0,46	WT	bacA mutant	PA14_06600	acyl-CoA dehydrogenase
YP_788678.1|gi|116054234|	10	611	3,53E-01	3,64E-01	1,33	0,13	WT	bacA mutant	PA14_06640	acyl-CoA dehydrogenase
YP_788689.1|gi|116054245|	8	488	3,05E-02	1,01E-01	1,17	0,69	bacA mutant	WT	PA14_06750	nitrite reductase
YP_788697.1|gi|116054253|	3	114	6,82E-01	5,07E-01	1,05	0,06	WT	bacA mutant	PA14_06870	transcriptional regulator Dnr
YP_788707.1|gi|116054263|	4	230	8,03E-01	5,42E-01	1,05	0,06	WT	bacA mutant	PA14_06980	hypothetical protein
YP_788708.1|gi|116054264|	13	1077	5,94E-01	4,71E-01	1,21	0,07	WT	bacA mutant	PA14_06990	hypothetical protein

YP_788712.1|gi|116054268|	3	133	2,28E-01	2,85E-01	1,15	0,20	WT	bacA mutant	PA14_07030	cytochrome c'
YP_788717.1|gi|116054273|	12	699	2,66E-02	9,67E-02	1,18	0,73	WT	bacA mutant	PA14_07090	S-adenosylmethionine synthetase
YP_788719.1|gi|116054275|	20	939	5,43E-01	4,52E-01	1,15	0,08	WT	bacA mutant	PA14_07130	transketolase
YP_788722.1|gi|116054278|	2	66	1,72E-02	7,92E-02	1,38	0,83	WT	bacA mutant	PA14_07170	D-erythrose 4-phosphate dehydrogenase
YP_788723.1|gi|116054279|	4	363	1,48E-01	2,19E-01	1,18	0,28	WT	bacA mutant	PA14_07190	phosphoglycerate kinase
YP_788726.1|gi|116054282|	16	916	6,92E-02	1,44E-01	1,20	0,47	WT	bacA mutant	PA14_07230	fructose-1,6-bisphosphate aldolase
YP_788729.1|gi|116054284|	8	442	8,70E-01	5,55E-01	1,01	0,05	bacA mutant	WT	PA14_07260	hypothetical protein
YP_788749.1|gi|116054304|	19	1064	4,76E-02	1,20E-01	1,42	0,57	bacA mutant	WT	PA14_07520	RNA polymerase sigma factor RpoD
YP_788752.1|gi|116054307|	6	331	9,92E-01	5,83E-01	1,01	0,05	WT	bacA mutant	PA14_07560	30S ribosomal protein S21
YP_788754.1|gi|116054309|	2	42	9,88E-01	5,82E-01	1,75	0,05	WT	bacA mutant	PA14_07580	glycerol-3-phosphate acyltransferase PlsY
YP_788755.1|gi|116054310|	2	58	6,85E-01	5,07E-01	1,12	0,06	WT	bacA mutant	PA14_07590	dihydroneopterin aldolase
YP_788759.1|gi|116054314|	11	478	3,77E-01	3,74E-01	1,32	0,12	WT	bacA mutant	PA14_07650	SpoVR family protein
YP_788760.1|gi|116054315|	12	708	3,11E-01	3,43E-01	1,34	0,15	WT	bacA mutant	PA14_07660	hypothetical protein
YP_788761.1|gi|116054316|	29	2033	5,55E-02	1,30E-01	1,22	0,53	WT	bacA mutant	PA14_07680	hypothetical protein
YP_788765.1|gi|116054320|	3	143	7,03E-02	1,44E-01	1,45	0,46	bacA mutant	WT	PA14_07730	dimethyladenosine transferase
YP_788766.1|gi|116054321|	6	274	8,43E-01	5,52E-01	1,01	0,05	WT	bacA mutant	PA14_07740	4-hydroxythreonine-4-phosphate dehydrogenase
YP_788767.1|gi|116054322|	16	1076	4,32E-02	1,16E-01	1,17	0,60	WT	bacA mutant	PA14_07760	peptidyl-prolyl cis-trans isomerase SurA
YP_788768.1|gi|116054323|	40	2416	5,04E-01	4,35E-01	1,10	0,09	bacA mutant	WT	PA14_07770	organic solvent tolerance protein OstA
YP_788769.1|gi|116054324|	5	231	5,49E-01	4,54E-01	1,05	0,08	bacA mutant	WT	PA14_07780	hypothetical protein
YP_788776.1|gi|116054331|	4	93	7,11E-01	5,18E-01	1,11	0,06	bacA mutant	WT	PA14_07860	ABC transporter ATP-binding protein
YP_788777.1|gi|116054332|; YP_788373.1|gi|116053935|	7	340	3,40E-02	1,06E-01	1,37	0,66	WT	bacA mutant	PA14_07870	ABC transporter substrate-binding protein
YP_788780.1|gi|116054335|	4	171	8,30E-03	6,00E-02	1,47	0,94	WT	bacA mutant	PA14_07910	ribulose-phosphate 3-epimerase
YP_788782.1|gi|116054337|	7	265	3,22E-01	3,49E-01	1,06	0,14	WT	bacA mutant	PA14_07940	anthranilate synthase component I
YP_788795.1|gi|116054350|	2	111	4,20E-02	1,16E-01	1,53	0,61	WT	bacA mutant	PA14_08070	phage tail sheath protein
YP_788822.1|gi|116054377|	4	140	2,97E-02	1,00E-01	1,80	0,70	WT	bacA mutant	PA14_08370	cAMP-regulatory protein

YP_788825.1|gi|116054380|	4	173	3,58E-01	3,66E-01	1,07	0,13	WT	bacA mutant	PA14_08400	hypothetical protein
YP_788827.1|gi|116054382|	2	90	1,59E-01	2,27E-01	1,28	0,27	bacA mutant	WT	PA14_08430	ATPase
YP_788828.1|gi|116054383|	5	236	1,57E-01	2,26E-01	1,21	0,27	bacA mutant	WT	PA14_08440	short chain alcohol dehydrogenase
YP_788829.1|gi|116054384|	5	126	7,37E-01	5,25E-01	1,29	0,06	WT	bacA mutant	PA14_08450	hypothetical protein
YP_788830.1|gi|116054385|	6	351	7,69E-02	1,52E-01	1,19	0,44	WT	bacA mutant	PA14_08460	hypothetical protein
YP_788832.1|gi|116054387|	11	691	9,92E-01	5,83E-01	1,01	0,05	WT	bacA mutant	PA14_08480	N-acetyl-gamma-glutamyl-phosphate reductase
YP_788835.1|gi|116054390|	3	98	7,67E-02	1,52E-01	1,47	0,44	WT	bacA mutant	PA14_08510	iron-sulfur cluster insertion protein ErpA
YP_788836.1|gi|116054391|	2	88	2,36E-01	2,91E-01	1,20	0,19	bacA mutant	WT	PA14_08520	anhydro-N-acetylmuramic acid kinase
YP_788838.1|gi|116054393|	8	400	3,14E-02	1,03E-01	1,28	0,69	WT	bacA mutant	PA14_08560	tyrosyl-tRNA synthetase
YP_788840.1|gi|116052313|	3	172	7,13E-01	5,18E-01	1,04	0,06	bacA mutant	WT	PA14_08630	pantothenate kinase
YP_788842.1|gi|116052311|	42	4578	1,65E-03	3,84E-02	1,29	1,00	WT	bacA mutant	PA14_08680	elongation factor Tu
YP_788844.1|gi|116052310|	9	675	1,50E-03	3,69E-02	1,41	1,00	WT	bacA mutant	PA14_08710	transcription antitermination protein NusG
YP_788845.1|gi|116052309|	6	529	5,15E-01	4,42E-01	1,13	0,09	bacA mutant	WT	PA14_08720	50S ribosomal protein L11
YP_788846.1|gi|116052308|	18	1465	4,70E-01	4,22E-01	1,17	0,10	bacA mutant	WT	PA14_08730	50S ribosomal protein L1
YP_788847.1|gi|116052307|	4	384	1,50E-01	2,21E-01	1,21	0,28	WT	bacA mutant	PA14_08740	50S ribosomal protein L10
YP_788848.1|gi|116052306|	4	259	1,30E-02	6,92E-02	2,03	0,88	WT	bacA mutant	PA14_08750	50S ribosomal protein L7/L12
YP_788849.1|gi|116052305|	61	3641	1,98E-01	2,60E-01	1,33	0,22	WT	bacA mutant	PA14_08760	DNA-directed RNA polymerase subunit beta
YP_788850.1|gi|116052304|	60	3575	2,46E-01	2,98E-01	1,26	0,18	WT	bacA mutant	PA14_08780	DNA-directed RNA polymerase subunit beta'
YP_788851.1|gi|116052303|	6	292	3,71E-01	3,73E-01	1,25	0,12	WT	bacA mutant	PA14_08790	30S ribosomal protein S12
YP_788852.1|gi|116052302|	9	495	9,95E-01	5,84E-01	1,06	0,05	bacA mutant	WT	PA14_08810	30S ribosomal protein S7
YP_788853.1|gi|116052301|	51	4008	4,95E-02	1,21E-01	1,43	0,56	bacA mutant	WT	PA14_08820	elongation factor G
YP_788855.1|gi|116052299|	4	289	9,55E-01	5,72E-01	1,00	0,05	WT	bacA mutant	PA14_08840	30S ribosomal protein S10
YP_788856.1|gi|116052298|	6	709	6,13E-01	4,80E-01	1,12	0,07	bacA mutant	WT	PA14_08850	50S ribosomal protein L3
YP_788857.1|gi|116052297|	12	1028	6,26E-03	5,40E-02	1,44	0,97	bacA mutant	WT	PA14_08860	50S ribosomal protein L4
YP_788858.1|gi|116052296|	4	129	5,16E-01	4,42E-01	1,12	0,09	bacA mutant	WT	PA14_08870	50S ribosomal protein L23
YP_788859.1|gi|116052295|	13	868	8,28E-01	5,49E-01	1,24	0,05	bacA mutant	WT	PA14_08880	50S ribosomal protein L2

YP_788860.1|gi|116052294|	3	152	8,24E-01	5,48E-01	1,05	0,05	bacA mutant	WT	PA14_08890	30S ribosomal protein S19
YP_788861.1|gi|116052293|	6	479	5,09E-01	4,38E-01	1,20	0,09	bacA mutant	WT	PA14_08900	50S ribosomal protein L22
YP_788862.1|gi|116052292|	12	793	9,06E-01	5,59E-01	1,09	0,05	bacA mutant	WT	PA14_08910	30S ribosomal protein S3
YP_788863.1|gi|116052291|	7	476	8,81E-01	5,56E-01	1,13	0,05	bacA mutant	WT	PA14_08920	50S ribosomal protein L16
YP_788864.1|gi|116052290|	2	128	2,60E-01	3,08E-01	1,33	0,17	bacA mutant	WT	PA14_08930	50S ribosomal protein L29
YP_788865.1|gi|116052289|	3	168	6,02E-02	1,35E-01	1,32	0,51	bacA mutant	WT	PA14_08940	30S ribosomal protein S17
YP_788866.1|gi|116052288|	13	1029	4,72E-01	4,23E-01	1,24	0,10	bacA mutant	WT	PA14_08950	50S ribosomal protein L14
YP_788867.1|gi|116052287|	7	433	4,03E-01	3,88E-01	1,18	0,11	WT	bacA mutant	PA14_08960	50S ribosomal protein L24
YP_788868.1|gi|116052286|	17	1213	6,55E-01	4,96E-01	1,17	0,07	bacA mutant	WT	PA14_08970	50S ribosomal protein L5
YP_788869.1|gi|116052285|	2	42	5,37E-01	4,50E-01	1,13	0,08	bacA mutant	WT	PA14_08980	30S ribosomal protein S14
YP_788870.1|gi|116052284|	4	179	1,21E-01	1,98E-01	1,52	0,33	WT	bacA mutant	PA14_08990	30S ribosomal protein S8
YP_788871.1|gi|116052283|	11	801	8,20E-01	5,47E-01	1,13	0,05	bacA mutant	WT	PA14_09000	50S ribosomal protein L6
YP_788872.1|gi|116052282|	7	661	3,02E-01	3,39E-01	1,27	0,15	bacA mutant	WT	PA14_09010	50S ribosomal protein L18
YP_788873.1|gi|116052281|	12	1183	6,56E-01	4,96E-01	1,07	0,07	WT	bacA mutant	PA14_09020	30S ribosomal protein S5
YP_788875.1|gi|116052279|	6	454	8,48E-01	5,52E-01	1,11	0,05	bacA mutant	WT	PA14_09040	50S ribosomal protein L15
YP_788876.1|gi|116052278|	6	260	7,88E-01	5,39E-01	1,03	0,06	WT	bacA mutant	PA14_09050	preprotein translocase subunit SecY
YP_788878.1|gi|116052276|	8	404	9,60E-01	5,73E-01	1,03	0,05	bacA mutant	WT	PA14_09080	30S ribosomal protein S13
YP_788879.1|gi|116052275|	5	325	2,89E-01	3,30E-01	1,36	0,16	bacA mutant	WT	PA14_09090	30S ribosomal protein S11
YP_788880.1|gi|116052274|	14	980	5,91E-01	4,69E-01	1,22	0,07	bacA mutant	WT	PA14_09100	30S ribosomal protein S4
YP_788881.1|gi|116054394|	15	1038	3,21E-01	3,49E-01	1,06	0,14	WT	bacA mutant	PA14_09115	DNA-directed RNA polymerase subunit alpha
YP_788882.1|gi|116052273|	7	351	9,01E-01	5,59E-01	1,02	0,05	bacA mutant	WT	PA14_09130	50S ribosomal protein L17
YP_788883.1|gi|116052272|	33	2700	3,29E-01	3,52E-01	1,08	0,14	bacA mutant	WT	PA14_09150	catalase
YP_788884.1|gi|116052271|	12	611	3,03E-01	3,39E-01	1,28	0,15	WT	bacA mutant	PA14_09160	bacterioferritin
YP_788885.1|gi|116052270|	5	268	4,09E-01	3,91E-01	1,64	0,11	WT	bacA mutant	PA14_09180	excinuclease ABC subunit A
YP_788886.1|gi|116054452|	2	81	8,71E-01	5,55E-01	1,18	0,05	WT	bacA mutant	PA14_09195	major facilitator transporter
YP_788887.1|gi|116052269|	4	300	1,54E-02	7,52E-02	1,96	0,85	WT	bacA mutant	PA14_09200	single-stranded DNA-binding protein

YP_788888.1|gi|116052268|	5	193	1,48E-01	2,19E-01	1,47	0,28	bacA mutant	WT	PA14_09210	salicylate biosynthesis isochorismate synthase
YP_788889.1|gi|116052267|	2	94	1,22E-01	1,98E-01	1,49	0,33	bacA mutant	WT	PA14_09220	isochorismate-pyruvate lyase
YP_788891.1|gi|116052265|	8	400	4,69E-02	1,20E-01	1,29	0,57	bacA mutant	WT	PA14_09240	pyochelin biosynthesis protein PchD
YP_788893.1|gi|116052263|	19	963	7,87E-01	5,39E-01	1,23	0,06	WT	bacA mutant	PA14_09270	dihydroaeruginoic acid synthetase
YP_788894.1|gi|116052262|	15	737	9,81E-01	5,79E-01	1,05	0,05	WT	bacA mutant	PA14_09280	pyochelin synthetase
YP_788895.1|gi|116052261|	6	374	1,34E-02	6,96E-02	1,32	0,88	bacA mutant	WT	PA14_09290	pyochelin biosynthetic protein PchG
YP_788896.1|gi|116052260|	3	226	8,93E-01	5,58E-01	1,24	0,05	WT	bacA mutant	PA14_09300	ABC transporter ATP-binding protein
YP_788897.1|gi|116052259|	6	368	7,97E-01	5,41E-01	1,35	0,06	WT	bacA mutant	PA14_09320	ABC transporter ATP-binding protein
YP_788898.1|gi|116052258|	20	1265	6,33E-02	1,38E-01	1,40	0,49	WT	bacA mutant	PA14_09340	Fe(III)-pyochelin outer membrane receptor
YP_788901.1|gi|116052255|	2	144	9,88E-01	5,82E-01	1,50	0,05	WT	bacA mutant	PA14_09380	transporter
YP_788902.1|gi|116052254|	15	790	4,30E-02	1,16E-01	1,31	0,60	WT	bacA mutant	PA14_09400	hypothetical protein
YP_788903.1|gi|116052253|	5	437	9,39E-01	5,71E-01	1,02	0,05	bacA mutant	WT	PA14_09410	pyrodoxamine 5'-phosphate oxidase
YP_788904.1|gi|116052252|	10	850	1,08E-02	6,67E-02	1,28	0,91	bacA mutant	WT	PA14_09420	phenazine biosynthesis protein
YP_788905.1|gi|116052251|; YP_791336.1|gi|116049855|	12	704	6,79E-02	1,43E-01	1,43	0,47	bacA mutant	WT	PA14_09440	phenazine biosynthesis protein PhzE
YP_788906.1|gi|116052250|	8	364	2,07E-01	2,67E-01	1,21	0,21	bacA mutant	WT	PA14_09450	phenazine biosynthesis protein PhzD
YP_788907.1|gi|116052249|	8	319	1,75E-01	2,40E-01	1,39	0,25	bacA mutant	WT	PA14_09460	phenazine biosynthesis protein PhzC
YP_788908.1|gi|116052248|	7	398	3,67E-02	1,09E-01	1,56	0,64	WT	bacA mutant	PA14_09470	phenazine biosynthesis protein
YP_788910.1|gi|116052246|	7	427	6,83E-01	5,07E-01	1,08	0,06	WT	bacA mutant	PA14_09490	phenazine-specific methyltransferase
YP_788911.1|gi|116052245|	20	1830	3,02E-01	3,39E-01	1,17	0,15	WT	bacA mutant	PA14_09500	outer membrane protein
YP_788912.1|gi|116052244|	14	533	8,06E-01	5,43E-01	1,24	0,05	WT	bacA mutant	PA14_09520	RND efflux transporter
YP_788913.1|gi|116052243|	10	680	3,55E-01	3,65E-01	1,18	0,13	WT	bacA mutant	PA14_09530	RND efflux membrane fusion protein
YP_788914.1|gi|116052242|	5	410	6,90E-01	5,10E-01	1,65	0,06	WT	bacA mutant	PA14_09540	hypothetical protein
YP_788915.1|gi|116052241|	6	387	4,82E-03	4,75E-02	1,54	0,98	WT	bacA mutant	PA14_09550	hypothetical protein
YP_788917.1|gi|116052239|	2	70	1,62E-02	7,70E-02	1,50	0,84	WT	bacA mutant	PA14_09580	hypothetical protein
YP_788918.1|gi|116052238|	2	133	4,74E-02	1,20E-01	1,15	0,57	bacA mutant	WT	PA14_09600	D-alanine--D-alanine ligase

YP_788920.1|gi|116052236|	12	691	8,42E-01	5,52E-01	1,09	0,05	WT	bacA mutant	PA14_09630	acyl-CoA dehydrogenase
YP_788924.1|gi|116052232|	5	201	2,88E-01	3,29E-01	1,14	0,16	bacA mutant	WT	PA14_09700	monooxygenase
YP_788934.1|gi|116052222|	13	703	7,79E-01	5,38E-01	1,04	0,06	bacA mutant	WT	PA14_09820	acetolactate synthase
YP_788938.1|gi|116052218|	3	165	7,20E-01	5,19E-01	1,44	0,06	bacA mutant	WT	PA14_09890	peptidyl-prolyl cis-trans isomerase C2
YP_788951.1|gi|116052205|	9	533	4,95E-01	4,33E-01	1,04	0,09	WT	bacA mutant	PA14_10040	amidase
YP_788968.1|gi|116052188|	3	77	6,31E-01	4,86E-01	1,09	0,07	WT	bacA mutant	PA14_10240	branched-chain alpha-keto acid dehydrogenase subunit E2
YP_788969.1|gi|116052187|	3	264	1,54E-01	2,24E-01	1,28	0,27	bacA mutant	WT	PA14_10250	acetoin catabolism protein AcoB
YP_788976.1|gi|116052180|	9	673	6,80E-01	5,06E-01	1,11	0,06	bacA mutant	WT	PA14_10330	outer membrane protein
YP_788980.1|gi|116052176|	4	184	8,08E-02	1,57E-01	1,36	0,43	bacA mutant	WT	PA14_10370	hypothetical protein
YP_788989.1|gi|116052167|	2	107	9,67E-01	5,75E-01	1,20	0,05	WT	bacA mutant	PA14_10500	cbb3-type cytochrome c oxidase subunit I
YP_788990.1|gi|116052166|	7	304	5,15E-01	4,42E-01	1,07	0,09	WT	bacA mutant	PA14_10530	GntR family transcriptional regulator
YP_788991.1|gi|116052165|	5	261	9,05E-01	5,59E-01	1,35	0,05	WT	bacA mutant	PA14_10540	iron-sulfur cluster-binding protein
YP_788992.1|gi|116052164|	11	731	2,98E-01	3,36E-01	1,15	0,15	bacA mutant	WT	PA14_10550	sulfite or nitrite reductas
YP_788993.1|gi|116052163|	3	163	1,33E-01	2,09E-01	1,13	0,30	bacA mutant	WT	PA14_10560	hypothetical protein
YP_789005.1|gi|116052151|	3	87	5,11E-01	4,39E-01	1,59	0,09	WT	bacA mutant	PA14_10700	bacteriophytochrome
YP_789007.1|gi|116052149|	2	127	4,41E-01	4,08E-01	1,27	0,10	bacA mutant	WT	PA14_10730	hypothetical protein
YP_789017.1|gi|116052139|	2	59	2,43E-02	9,39E-02	14,08	0,75	bacA mutant	WT	PA14_10850	dehydrogenase
YP_789029.1|gi|116052127|	4	164	4,33E-01	4,06E-01	1,15	0,10	bacA mutant	WT	PA14_11000	4-hydroxyphenylacetate 3-monooxygenase large chain
YP_789041.1|gi|116052115|	5	273	2,32E-02	9,26E-02	1,29	0,76	WT	bacA mutant	PA14_11130	short chain dehydrogenase
YP_789042.1|gi|116052114|	46	2961	4,04E-02	1,14E-01	1,70	0,62	WT	bacA mutant	PA14_11140	nonribosomal peptide synthetase
YP_789052.1|gi|116052104|	4	244	9,11E-01	5,61E-01	1,01	0,05	bacA mutant	WT	PA14_11260	epimerase
YP_789053.1|gi|116052103|	14	1297	3,16E-01	3,45E-01	1,26	0,15	WT	bacA mutant	PA14_11270	outer membrane protein OprG precursor
YP_789059.1|gi|116052097|	11	1205	6,63E-01	4,99E-01	1,05	0,07	bacA mutant	WT	PA14_11340	thioredoxin
YP_789061.1|gi|116052095|	2	85	4,37E-01	4,07E-01	1,17	0,10	WT	bacA mutant	PA14_11370	lipoprotein
YP_789063.1|gi|116052093|	3	222	3,78E-02	1,11E-01	1,33	0,63	WT	bacA mutant	PA14_11400	riboflavin-specific deaminase/reductase
YP_789065.1|gi|116052091|	8	420	1,31E-02	6,92E-02	1,32	0,88	WT	bacA mutant	PA14_11420	bifunctional 3,4-dihydroxy-2-butanone 4-phosphate synthase/GTP cyclohydrolase II-like protein

YP_789066.1|gi|116052090|	6	552	2,96E-01	3,34E-01	1,11	0,15	WT	bacA mutant	PA14_11430	6,7-dimethyl-8-ribityllumazine synthase
YP_789067.1|gi|116052089|	2	71	3,38E-02	1,06E-01	1,27	0,67	bacA mutant	WT	PA14_11450	transcription antitermination protein NusB
YP_789072.1|gi|116052084|	2	96	3,00E-01	3,37E-01	1,11	0,15	bacA mutant	WT	PA14_11510	GTP cyclohydrolase II
YP_789075.1|gi|116052081|	6	369	4,05E-01	3,89E-01	1,09	0,11	bacA mutant	WT	PA14_11550	1-deoxy-D-xylulose-5-phosphate synthase
YP_789088.1|gi|116052068|	7	392	1,79E-03	3,92E-02	2,13	1,00	WT	bacA mutant	PA14_11690	inorganic pyrophosphatase
YP_789093.1|gi|116052063|	2	126	1,36E-01	2,11E-01	1,40	0,30	WT	bacA mutant	PA14_11750	acetyltransferase
YP_789097.1|gi|116052059|; YP_791255.1|gi|116049936|	21	1462	5,67E-01	4,60E-01	1,04	0,08	bacA mutant	WT	PA14_11810	aldehyde dehydrogenase
YP_789099.1|gi|116054451|	5	218	6,30E-01	4,86E-01	1,08	0,07	WT	bacA mutant	PA14_11845	UDP-N-acetylmuramate:L-alanyl-gamma-D-glutamyl- meso-diaminopimelate ligase
YP_789100.1|gi|116052057|	2	153	9,11E-02	1,69E-01	1,42	0,40	WT	bacA mutant	PA14_11860	aromatic acid decarboxylase
YP_789102.1|gi|116052055|	4	162	2,91E-02	9,95E-02	1,37	0,71	WT	bacA mutant	PA14_11890	hypothetical protein
YP_789104.1|gi|116052053|	6	250	2,10E-02	8,96E-02	1,33	0,79	WT	bacA mutant	PA14_11910	hypothetical protein
YP_789108.1|gi|116052049|	2	100	6,29E-01	4,86E-01	1,51	0,07	WT	bacA mutant	PA14_11960	hypothetical protein
YP_789112.1|gi|116052045|	9	510	7,52E-01	5,28E-01	1,02	0,06	WT	bacA mutant	PA14_12010	gamma-glutamyl phosphate reductase
YP_789118.1|gi|116052039|	4	165	2,60E-01	3,08E-01	1,22	0,17	WT	bacA mutant	PA14_12080	soluble lytic transglycosylase B
YP_789120.1|gi|116052037|	11	697	6,86E-01	5,08E-01	1,02	0,06	WT	bacA mutant	PA14_12100	D-ala-D-ala-carboxypeptidase
YP_789121.1|gi|116052036|	2	87	6,76E-01	5,05E-01	1,28	0,06	bacA mutant	WT	PA14_12110	hypothetical protein
YP_789123.1|gi|116052034|	4	152	6,17E-01	4,82E-01	1,06	0,07	bacA mutant	WT	PA14_12130	lipoyl synthase
YP_789126.1|gi|116052030|	5	225	9,34E-03	6,32E-02	1,48	0,93	WT	bacA mutant	PA14_12160	murein transglycosylase
YP_789129.1|gi|116052028|	2	105	1,66E-02	7,84E-02	1,86	0,84	bacA mutant	WT	PA14_12200	DNA polymerase III subunit delta
YP_789130.1|gi|116052027|	11	706	4,44E-03	4,75E-02	1,65	0,99	WT	bacA mutant	PA14_12210	hypothetical protein
YP_789131.1|gi|116052026|	5	182	4,59E-01	4,17E-01	1,22	0,10	WT	bacA mutant	PA14_12230	leucyl-tRNA synthetase
YP_789134.1|gi|116052023|	3	91	7,06E-01	5,16E-01	1,13	0,06	bacA mutant	WT	PA14_12280	apolipoprotein N-acyltransferase
YP_789135.1|gi|116052022|	4	196	4,32E-01	4,05E-01	1,17	0,10	bacA mutant	WT	PA14_12300	hypothetical protein
YP_789137.1|gi|116052020|	3	159	2,68E-04	2,46E-02	2,20	1,00	bacA mutant	WT	PA14_12330	hypothetical protein
YP_789138.1|gi|116052019|	4	244	9,53E-01	5,72E-01	1,00	0,05	bacA mutant	WT	PA14_12350	(dimethylallyl)adenosine tRNA methylthiotransferase

YP_789141.1|gi|116052016|	15	1059	1,07E-02	6,67E-02	1,30	0,91	WT	bacA mutant	PA14_12390	glutamate-1-semialdehyde aminotransferase
YP_789142.1|gi|116052015|	5	197	5,00E-01	4,34E-01	1,05	0,09	WT	bacA mutant	PA14_12400	thiamine-phosphate pyrophosphorylase
YP_789143.1|gi|116052014|	3	181	6,16E-02	1,36E-01	1,45	0,50	WT	bacA mutant	PA14_12410	phosphomethylpyrimidine kinase
YP_789145.1|gi|116052012|	7	451	2,27E-02	9,26E-02	1,15	0,77	WT	bacA mutant	PA14_12450	acyl-CoA dehydrogenase
YP_789147.1|gi|116052010|	11	546	6,05E-01	4,76E-01	1,05	0,07	bacA mutant	WT	PA14_12490	AMP nucleosidase
YP_789152.1|gi|116052005|	3	215	3,50E-01	3,63E-01	1,14	0,13	WT	bacA mutant	PA14_12570	transcription regulator AsnC
YP_789159.1|gi|116051998|	2	87	9,79E-01	5,79E-01	1,10	0,05	WT	bacA mutant	PA14_12670	hypothetical protein
YP_789167.1|gi|116051990|	2	87	5,37E-01	4,50E-01	1,26	0,08	bacA mutant	WT	PA14_12760	ATP-dependent RNA helicase
YP_789177.1|gi|116051980|	3	214	1,96E-01	2,58E-01	1,38	0,22	WT	bacA mutant	PA14_12900	DNA binding protein
YP_789183.1|gi|116051974|	3	166	7,21E-01	5,19E-01	1,04	0,06	WT	bacA mutant	PA14_12980	hypothetical protein
YP_789187.1|gi|116051970|	2	54	8,78E-01	5,55E-01	1,52	0,05	WT	bacA mutant	PA14_13030	CioA, cyanide insensitive terminal oxidase
YP_789192.1|gi|116051965|	4	240	7,57E-01	5,29E-01	1,08	0,06	bacA mutant	WT	PA14_13090	acyl-CoA thiolase
YP_789193.1|gi|116051964|	11	705	4,83E-02	1,20E-01	1,18	0,57	WT	bacA mutant	PA14_13110	long-chain-fatty-acid--CoA ligase
YP_789194.1|gi|116051963|	17	1856	3,12E-01	3,43E-01	1,45	0,15	WT	bacA mutant	PA14_13130	hypothetical protein
YP_789195.1|gi|116051962|	25	2161	1,58E-03	3,76E-02	1,62	1,00	WT	bacA mutant	PA14_13140	hypothetical protein
YP_789197.1|gi|116051960|	2	138	8,32E-01	5,49E-01	1,08	0,05	WT	bacA mutant	PA14_13170	metal transporting P-type ATPase
YP_789198.1|gi|116051959|	3	209	3,77E-03	4,75E-02	2,16	0,99	bacA mutant	WT	PA14_13190	hypothetical protein
YP_789212.1|gi|116051945|	5	231	4,11E-01	3,93E-01	1,10	0,11	bacA mutant	WT	PA14_13350	hypothetical protein
YP_789217.1|gi|116051940|	7	340	7,49E-02	1,49E-01	1,13	0,45	bacA mutant	WT	PA14_13410	peptide chain release factor 3
YP_789225.1|gi|116051932|	3	128	1,02E-01	1,81E-01	1,41	0,37	WT	bacA mutant	PA14_13510	LysR family transcriptional regulator
YP_789229.1|gi|116051928|	5	226	7,95E-02	1,56E-01	1,24	0,43	WT	bacA mutant	PA14_13580	ABC transporter ATP-binding protein
YP_789238.1|gi|116051919|	2	66	1,42E-01	2,16E-01	1,33	0,29	bacA mutant	WT	PA14_13680	short chain dehydrogenase
YP_789239.1|gi|116051918|	2	111	6,83E-01	5,07E-01	1,14	0,06	bacA mutant	WT	PA14_13690	methyltransferase
YP_789242.1|gi|116051915|	2	57	8,48E-01	5,52E-01	1,01	0,05	WT	bacA mutant	PA14_13730	transcriptional regulator NarL
YP_789246.1|gi|116051911|	2	46	7,12E-01	5,18E-01	1,24	0,06	bacA mutant	WT	PA14_13780	respiratory nitrate reductase alpha subun
YP_789247.1|gi|116051910|	2	115	6,25E-02	1,37E-01	1,45	0,50	bacA mutant	WT	PA14_13800	respiratory nitrate reductase beta subuni

YP_789264.1|gi|116051893|	3	88	4,36E-01	4,07E-01	1,12	0,10	bacA mutant	WT	PA14_14020	hypothetical protein
YP_789265.2|gi|161486758|	10	485	8,63E-01	5,53E-01	1,01	0,05	WT	bacA mutant	PA14_14040	ATP-dependent RNA helicase RhlB
YP_789266.1|gi|116051891|	6	198	4,57E-01	4,16E-01	1,09	0,10	bacA mutant	WT	PA14_14060	AMP-binding protein
YP_789267.1|gi|116051890|	2	151	7,15E-01	5,18E-01	1,08	0,06	bacA mutant	WT	PA14_14080	carboxylesterase
YP_789268.1|gi|116051889|	3	174	5,52E-04	2,67E-02	1,53	1,00	WT	bacA mutant	PA14_14100	amino acid-binding protein
YP_789271.1|gi|116051886|	3	138	2,46E-02	9,43E-02	1,55	0,75	WT	bacA mutant	PA14_14140	hypothetical protein
YP_789273.1|gi|116051884|	2	147	9,20E-02	1,70E-01	1,58	0,39	bacA mutant	WT	PA14_14160	acetyltransferase
YP_789274.1|gi|116051883|	3	90	2,24E-02	9,26E-02	1,46	0,77	WT	bacA mutant	PA14_14170	hypothetical protein
YP_789277.1|gi|116051880|;Y	10	756	1,06E-03	3,14E-02	1,47	1,00	WT	bacA mutant	PA14_14220	nucleoid-associated protein NdpA
YP_789278.1|gi|116051879|	18	1314	8,57E-01	5,53E-01	1,16	0,05	WT	bacA mutant	PA14_14230	hypothetical protein
YP_789280.1|gi|116051877|	3	130	3,18E-01	3,47E-01	1,25	0,14	bacA mutant	WT	PA14_14270	isochorismatase family hydrolase
YP_789291.1|gi|116051866|	11	809	1,00E-02	6,61E-02	1,78	0,92	WT	bacA mutant	PA14_14390	ABC-type transport protein, periplasmic c
YP_789295.1|gi|116051862|	2	103	9,59E-01	5,73E-01	1,11	0,05	WT	bacA mutant	PA14_14440	valyl-tRNA synthetase
YP_789296.1|gi|116051861|	2	74	5,00E-01	4,34E-01	1,16	0,09	WT	bacA mutant	PA14_14450	hypothetical protein
YP_789298.1|gi|116051859|	28	2358	7,39E-01	5,25E-01	1,05	0,06	WT	bacA mutant	PA14_14470	leucyl aminopeptidase
YP_789301.1|gi|116051856|	4	251	7,14E-01	5,18E-01	1,02	0,06	WT	bacA mutant	PA14_14500	hypothetical protein
YP_789308.1|gi|116051849|	2	71	2,47E-01	2,99E-01	1,17	0,18	bacA mutant	WT	PA14_14590	S-adenosylmethionine--tRNA ribosyltransferase-isomerase
YP_789310.1|gi|116051847|	4	192	8,10E-01	5,44E-01	1,13	0,05	WT	bacA mutant	PA14_14610	preprotein translocase subunit YajC
YP_789311.1|gi|116051846|	14	766	9,00E-01	5,59E-01	1,16	0,05	WT	bacA mutant	PA14_14630	preprotein translocase subunit SecD
YP_789312.1|gi|116051845|	4	399	9,75E-01	5,78E-01	1,26	0,05	WT	bacA mutant	PA14_14650	preprotein translocase subunit SecF
YP_789313.1|gi|116051844|	4	280	2,33E-03	4,16E-02	2,20	1,00	WT	bacA mutant	PA14_14660	hypothetical protein
YP_789314.1|gi|116051843|	5	285	1,64E-01	2,31E-01	1,08	0,26	WT	bacA mutant	PA14_14680	extragenic suppressor protein SuhB
YP_789315.1|gi|116051842|	5	222	4,52E-01	4,15E-01	1,08	0,10	bacA mutant	WT	PA14_14690	methyltransferase
YP_789316.1|gi|116051841|	5	274	8,45E-01	5,52E-01	1,02	0,05	WT	bacA mutant	PA14_14700	serine O-acetyltransferase
YP_789318.1|gi|116051839|	14	990	1,29E-01	2,05E-01	1,17	0,31	WT	bacA mutant	PA14_14730	cysteine desulfurase
YP_789319.1|gi|116051838|	2	67	1,70E-02	7,88E-02	2,45	0,83	WT	bacA mutant	PA14_14740	scaffold protein

YP_789322.1|gi|116051835|	9	523	1,24E-02	6,84E-02	1,26	0,89	bacA mutant	WT	PA14_14780	chaperone protein HscA
YP_789325.1|gi|116051832|	11	907	4,00E-02	1,14E-01	1,26	0,62	WT	bacA mutant	PA14_14820	nucleoside diphosphate kinase
YP_789326.1|gi|116051831|	4	256	8,48E-01	5,52E-01	1,04	0,05	WT	bacA mutant	PA14_14830	hypothetical protein
YP_789327.1|gi|116051830|	5	288	4,60E-02	1,19E-01	1,34	0,58	WT	bacA mutant	PA14_14850	type 4 fimbrial biogenesis protein PilF
YP_789328.1|gi|116051829|	6	578	9,08E-01	5,60E-01	1,01	0,05	WT	bacA mutant	PA14_14860	hypothetical protein
YP_789329.1|gi|116051828|	14	825	8,71E-04	2,90E-02	1,21	1,00	WT	bacA mutant	PA14_14880	4-hydroxy-3-methylbut-2-en-1-yl diphosphate synthase
YP_789330.1|gi|116051827|	11	675	2,36E-01	2,91E-01	1,18	0,19	bacA mutant	WT	PA14_14890	histidyl-tRNA synthetase
YP_789331.1|gi|116051826|	4	192	3,32E-01	3,54E-01	1,16	0,14	WT	bacA mutant	PA14_14900	hypothetical protein
YP_789332.1|gi|116051825|	6	320	2,66E-02	9,67E-02	1,76	0,73	WT	bacA mutant	PA14_14910	hypothetical protein
YP_789333.1|gi|116051824|	4	293	4,84E-02	1,20E-01	1,40	0,57	WT	bacA mutant	PA14_14930	GTP-binding protein EngA
YP_789334.1|gi|116051823|	4	209	3,78E-02	1,11E-01	1,20	0,63	WT	bacA mutant	PA14_14940	aminotransferase
YP_789335.1|gi|116051822|	3	82	2,82E-03	4,37E-02	1,58	1,00	WT	bacA mutant	PA14_14960	hypothetical protein
YP_789336.1|gi|116054441|	2	100	6,25E-01	4,86E-01	1,32	0,07	bacA mutant	WT	PA14_14975	hypothetical protein
YP_789337.1|gi|116051821|	5	459	3,93E-01	3,82E-01	1,10	0,12	WT	bacA mutant	PA14_14990	oxidoreductase
YP_789340.1|gi|116051818|	12	478	1,52E-03	3,69E-02	1,36	1,00	bacA mutant	WT	PA14_15030	2-isopropylmalate synthase
YP_789342.1|gi|116051816|	44	3349	3,27E-01	3,52E-01	1,20	0,14	WT	bacA mutant	PA14_15070	outer membrane copper receptor OprC
YP_789345.1|gi|116051813|	3	147	7,36E-01	5,25E-01	1,15	0,06	bacA mutant	WT	PA14_15100	hypothetical protein
YP_789347.1|gi|116051811|	4	141	3,41E-02	1,06E-01	1,67	0,66	WT	bacA mutant	PA14_15120	hypothetical protein
YP_789363.1|gi|116051795|	27	2340	6,37E-02	1,38E-01	1,27	0,49	WT	bacA mutant	PA14_15310	inosine 5'-monophosphate dehydrogenase
YP_789364.1|gi|116051794|	26	1557	5,35E-01	4,50E-01	1,11	0,08	bacA mutant	WT	PA14_15340	GMP synthase
YP_789365.1|gi|116051793|	3	138	2,84E-02	9,88E-02	1,84	0,71	WT	bacA mutant	PA14_15350	integrase
YP_789387.1|gi|116051774|	11	546	8,88E-04	2,90E-02	5,16	1,00	WT	bacA mutant	PA14_15590	hypothetical protein
YP_789398.1|gi|116051763|	4	142	8,39E-01	5,51E-01	1,02	0,05	WT	bacA mutant	PA14_15720	transglycosylase
YP_789399.1|gi|116051762|	3	184	6,11E-02	1,36E-01	1,82	0,50	WT	bacA mutant	PA14_15740	phosphoribosylformylglycinamidine synthase
YP_789409.1|gi|116051752|	2	51	8,48E-01	5,52E-01	1,22	0,05	WT	bacA mutant	PA14_15860	hypothetical protein
YP_789410.1|gi|116051751|	4	159	2,42E-01	2,95E-01	1,51	0,19	WT	bacA mutant	PA14_15870	hypothetical protein

YP_789412.1|gi|116051749|	12	654	5,60E-01	4,58E-01	1,05	0,08	bacA mutant	WT	PA14_15890	phosphoribosylglycinamide formyltransferase 2
YP_789417.1|gi|116051744|	10	525	4,34E-01	4,06E-01	1,10	0,10	bacA mutant	WT	PA14_15960	signal recognition particle protein Ffh
YP_789418.1|gi|116051743|	3	201	2,12E-02	9,05E-02	1,65	0,78	bacA mutant	WT	PA14_15970	30S ribosomal protein S16
YP_789421.1|gi|116051740|	9	638	3,08E-01	3,41E-01	1,29	0,15	bacA mutant	WT	PA14_16000	50S ribosomal protein L19
YP_789427.1|gi|116051734|	12	765	3,67E-02	1,09E-01	1,12	0,64	bacA mutant	WT	PA14_16070	homoserine dehydrogenase
YP_789428.1|gi|116051733|	11	854	3,73E-01	3,73E-01	1,11	0,12	bacA mutant	WT	PA14_16090	threonine synthase
YP_789431.1|gi|116051730|	2	83	2,86E-01	3,28E-01	1,21	0,16	bacA mutant	WT	PA14_16130	hypothetical protein
YP_789432.1|gi|116051729|	2	160	5,01E-01	4,34E-01	1,11	0,09	WT	bacA mutant	PA14_16140	hypothetical protein
YP_789433.1|gi|116051728|	15	1272	3,64E-04	2,46E-02	7,24	1,00	bacA mutant	WT	PA14_16150	hypothetical protein
YP_789434.1|gi|116051727|	3	194	4,24E-03	4,75E-02	7,94	0,99	bacA mutant	WT	PA14_16160	hypothetical protein
YP_789435.1|gi|116051726|	39	2588	3,05E-04	2,46E-02	13,50	1,00	bacA mutant	WT	PA14_16180	hypothetical protein
YP_789436.1|gi|116051725|	13	568	2,20E-04	2,46E-02	8,92	1,00	bacA mutant	WT	PA14_16190	hypothetical protein
YP_789437.1|gi|116051724|	7	382	7,90E-06	6,98E-03	6,13	1,00	bacA mutant	WT	PA14_16200	hypothetical protein
YP_789440.1|gi|116051721|	26	2432	4,67E-04	2,65E-02	2,65	1,00	WT	bacA mutant	PA14_16250	elastase LasB
YP_789441.1|gi|116051720|	16	1326	8,45E-01	5,52E-01	1,01	0,05	bacA mutant	WT	PA14_16260	FMN oxidoreductase
YP_789448.1|gi|116051713|	5	246	4,28E-03	4,75E-02	1,66	0,99	WT	bacA mutant	PA14_16330	hypothetical protein
YP_789452.1|gi|116051709|	9	555	2,03E-02	8,79E-02	1,46	0,79	WT	bacA mutant	PA14_16370	hypothetical protein
YP_789456.1|gi|116051705|	3	67	6,46E-01	4,92E-01	1,06	0,07	bacA mutant	WT	PA14_16430	chemotaxis transducer
YP_789462.1|gi|116051699|	2	61	6,81E-01	5,06E-01	1,11	0,06	WT	bacA mutant	PA14_16500	two-component response regulator
YP_789463.1|gi|116051698|	6	457	1,87E-01	2,51E-01	1,23	0,23	bacA mutant	WT	PA14_16510	peptide chain release factor 2
YP_789464.1|gi|116051697|	6	352	1,83E-03	3,93E-02	1,34	1,00	bacA mutant	WT	PA14_16530	lysyl-tRNA synthetase
YP_789465.1|gi|116051696|	4	219	4,87E-01	4,30E-01	1,14	0,09	WT	bacA mutant	PA14_16550	TetR family transcriptional regulator
YP_789466.1|gi|116051695|	5	212	2,38E-02	9,30E-02	1,43	0,76	WT	bacA mutant	PA14_16560	lipoprotein
YP_789467.1|gi|116051694|	6	344	4,96E-01	4,33E-01	1,25	0,09	WT	bacA mutant	PA14_16580	hypothetical protein
YP_789468.1|gi|116051693|	6	319	1,78E-01	2,43E-01	1,34	0,24	WT	bacA mutant	PA14_16590	hypothetical protein
YP_789470.1|gi|116051691|	2	68	5,16E-01	4,42E-01	1,16	0,09	bacA mutant	WT	PA14_16610	hypothetical protein

YP_789472.1|gi|116051689|	10	669	1,37E-02	7,10E-02	1,40	0,87	bacA mutant	WT	PA14_16630	outer membrane protein, OmpA
YP_789473.1|gi|116051688|	6	457	8,74E-01	5,55E-01	1,00	0,05	bacA mutant	WT	PA14_16640	lipoprotein
YP_789474.1|gi|116051687|	6	227	9,03E-01	5,59E-01	1,20	0,05	WT	bacA mutant	PA14_16660	metal-transporting P-type ATPase
YP_789476.1|gi|116051685|	2	44	5,26E-02	1,26E-01	1,89	0,54	WT	bacA mutant	PA14_16680	hypothetical protein
YP_789477.1|gi|116051684|	12	413	7,91E-01	5,39E-01	1,11	0,06	bacA mutant	WT	PA14_16690	phosphoenolpyruvate carboxylase
YP_789478.1|gi|116051683|	8	369	6,85E-02	1,44E-01	1,48	0,47	WT	bacA mutant	PA14_16700	adenylate kinase
YP_789482.1|gi|116051679|	3	165	1,83E-01	2,47E-01	1,27	0,24	WT	bacA mutant	PA14_16740	hypothetical protein
YP_789490.1|gi|116051671|	5	373	8,29E-01	5,49E-01	1,03	0,05	bacA mutant	WT	PA14_16840	lipoprotein
YP_789492.1|gi|116051669|	2	67	1,80E-02	8,08E-02	1,81	0,82	WT	bacA mutant	PA14_16870	ABC transporter ATP-binding protein
YP_789497.1|gi|116051664|	3	154	3,61E-02	1,08E-01	1,21	0,65	bacA mutant	WT	PA14_16930	pyridoxal-phosphate dependent protein
YP_789498.1|gi|116051663|	5	273	3,95E-01	3,83E-01	1,05	0,12	WT	bacA mutant	PA14_16950	tetrahydrodipicolinate succinylase
YP_789505.1|gi|116051656|	3	109	1,70E-02	7,88E-02	1,17	0,83	bacA mutant	WT	PA14_17030	succinyldiaminopimelate transaminase
YP_789507.1|gi|116051654|	5	249	2,77E-02	9,88E-02	1,38	0,72	WT	bacA mutant	PA14_17050	methionine aminopeptidase
YP_789508.1|gi|116051653|	16	1401	9,99E-01	5,85E-01	1,01	0,05	bacA mutant	WT	PA14_17060	30S ribosomal protein S2
YP_789509.1|gi|116051652|	20	1283	3,45E-02	1,06E-01	1,45	0,66	WT	bacA mutant	PA14_17070	elongation factor Ts
YP_789510.1|gi|116051651|	9	344	2,78E-03	4,37E-02	1,92	1,00	WT	bacA mutant	PA14_17080	uridylate kinase
YP_789511.1|gi|116051650|	11	710	3,91E-02	1,13E-01	1,77	0,63	WT	bacA mutant	PA14_17100	ribosome recycling factor
YP_789512.1|gi|116051649|	3	115	7,58E-01	5,30E-01	1,14	0,06	WT	bacA mutant	PA14_17110	UDP pyrophosphate synthetase
YP_789514.1|gi|116051647|	4	180	3,75E-02	1,11E-01	1,48	0,64	bacA mutant	WT	PA14_17130	1-deoxy-D-xylulose 5-phosphate reductoisomerase
YP_789515.1|gi|116051646|	2	66	4,16E-01	3,96E-01	1,33	0,11	bacA mutant	WT	PA14_17140	membrane-associated zinc metalloprotease
YP_789516.1|gi|116051645|	17	1117	2,97E-01	3,35E-01	1,12	0,15	bacA mutant	WT	PA14_17150	outer membrane antigen
YP_789517.1|gi|116051644|	6	374	5,23E-05	1,92E-02	1,95	1,00	WT	bacA mutant	PA14_17170	hypothetical protein
YP_789518.1|gi|116051643|	4	200	9,03E-01	5,59E-01	1,01	0,05	WT	bacA mutant	PA14_17180	UDP-3-O-[3-hydroxymyristoyl] glucosamine N-acyltransferase
YP_789519.1|gi|116051642|	7	412	6,85E-01	5,07E-01	1,05	0,06	WT	bacA mutant	PA14_17190	(3R)-hydroxymyristoyl-ACP dehydratase
YP_789520.1|gi|116051641|	2	84	3,58E-01	3,66E-01	1,12	0,13	WT	bacA mutant	PA14_17210	UDP-N-acetylglucosamine acyltransferase
YP_789523.1|gi|116051638|	5	267	5,98E-01	4,72E-01	1,13	0,07	bacA mutant	WT	PA14_17250	amino acid permease

YP_789525.1|gi|116051636|	13	1008	2,37E-02	9,30E-02	1,22	0,76	WT	bacA mutant	PA14_17270	acetyl-CoA carboxylase carboxyltransferase subunit alpha
YP_789527.1|gi|116051634|	31	2140	2,02E-01	2,63E-01	1,10	0,22	bacA mutant	WT	PA14_17290	CTP synthetase
YP_789528.1|gi|116051633|	5	208	2,79E-03	4,37E-02	1,42	1,00	WT	bacA mutant	PA14_17310	2-dehydro-3-deoxyphosphooctonate aldolase
YP_789529.1|gi|116051632|	16	1525	8,03E-01	5,42E-01	1,01	0,06	WT	bacA mutant	PA14_17320	phosphopyruvate hydratase
YP_789531.1|gi|116051630|	2	69	5,00E-01	4,34E-01	1,19	0,09	bacA mutant	WT	PA14_17340	2-C-methyl-D-erythritol 4-phosphate cytidylyltransferase
YP_789535.1|gi|116051626|	6	251	7,17E-03	5,85E-02	1,78	0,96	WT	bacA mutant	PA14_17400	alcohol dehydrogenase
YP_789536.1|gi|116051625|	2	54	3,54E-01	3,64E-01	1,59	0,13	WT	bacA mutant	PA14_17410	esterase
YP_789537.1|gi|116051624|	3	231	1,01E-01	1,81E-01	1,31	0,37	WT	bacA mutant	PA14_17420	2-C-methyl-D-erythritol 2,4-cyclodiphosphate synthase
YP_789538.1|gi|116051623|	6	218	2,88E-01	3,30E-01	1,10	0,16	bacA mutant	WT	PA14_17440	tRNA pseudouridine synthase D
YP_789539.1|gi|116051622|	3	135	8,11E-01	5,45E-01	1,03	0,05	bacA mutant	WT	PA14_17450	stationary phase survival protein SurE
YP_789541.1|gi|116051620|	2	53	7,87E-01	5,39E-01	1,41	0,06	WT	bacA mutant	PA14_17470	hypothetical protein
YP_789542.1|gi|116051619|	7	294	3,27E-02	1,05E-01	1,25	0,67	WT	bacA mutant	PA14_17480	RNA polymerase sigma factor RpoS
YP_789547.1|gi|116051614|	14	768	7,63E-01	5,32E-01	1,05	0,06	WT	bacA mutant	PA14_17530	recombinase A
YP_789549.1|gi|116051612|	6	288	9,18E-01	5,63E-01	1,00	0,05	WT	bacA mutant	PA14_17550	hypothetical protein
YP_789550.1|gi|116051611|	14	698	1,24E-02	6,84E-02	1,30	0,89	bacA mutant	WT	PA14_17570	hypothetical protein
YP_789551.1|gi|116051610|	11	644	3,10E-01	3,43E-01	1,22	0,15	bacA mutant	WT	PA14_17580	hypothetical protein
YP_789553.1|gi|116051608|	2	176	1,62E-01	2,29E-01	1,18	0,26	WT	bacA mutant	PA14_17600	hypothetical protein
YP_789560.1|gi|116051601|	5	334	2,86E-02	9,92E-02	1,14	0,71	WT	bacA mutant	PA14_17670	LuxR family transcriptional regulator
YP_789562.1|gi|116051600|	9	417	1,12E-01	1,90E-01	1,42	0,34	WT	bacA mutant	PA14_17690	hypothetical protein
YP_789577.1|gi|116051585|	2	108	9,72E-01	5,77E-01	1,08	0,05	WT	bacA mutant	PA14_17900	transcriptional regulator MetR
YP_789580.1|gi|116051582|	4	168	1,11E-02	6,67E-02	1,23	0,91	bacA mutant	WT	PA14_17930	glycerol-3-phosphate dehydrogenase
YP_789581.1|gi|116051581|	3	110	5,09E-01	4,38E-01	1,16	0,09	WT	bacA mutant	PA14_17940	glycerol-3-phosphate regulon repressor
YP_789582.1|gi|116051580|	17	857	3,58E-02	1,08E-01	1,21	0,65	WT	bacA mutant	PA14_17960	glycerol kinase
YP_789585.1|gi|116051577|	2	55	4,57E-01	4,16E-01	1,15	0,10	bacA mutant	WT	PA14_18010	glycerol kinase
YP_789595.1|gi|116051567|	9	877	1,54E-01	2,24E-01	1,23	0,27	WT	bacA mutant	PA14_18120	methylmalonate-semialdehyde dehydrogenase
YP_789596.1|gi|116051566|	2	117	9,88E-03	6,57E-02	2,00	0,92	WT	bacA mutant	PA14_18140	3-hydroxyisobutyrate dehydrogenase

YP_789598.1|gi|116051564|	5	199	2,66E-01	3,13E-01	1,15	0,17	WT	bacA mutant	PA14_18160	oxidoreductase
YP_789602.1|gi|116051560|	5	259	3,93E-01	3,82E-01	1,08	0,12	bacA mutant	WT	PA14_18230	DNA-binding transcriptional regulator FruR
YP_789625.1|gi|116051539|	2	50	7,66E-03	5,89E-02	1156,62	0,95	WT	bacA mutant	PA14_18580	GDP-mannose 6-dehydrogenase AlgD
YP_789626.1|gi|116051538|	2	99	4,41E-01	4,08E-01	1,11	0,10	bacA mutant	WT	PA14_18590	hypothetical protein
YP_789627.1|gi|116051537|	3	86	3,20E-01	3,49E-01	1,23	0,14	bacA mutant	WT	PA14_18600	ABC transporter ATP-binding protein
YP_789628.1|gi|116051536|	5	164	1,62E-01	2,29E-01	1,20	0,26	WT	bacA mutant	PA14_18610	ornithine carbamoyltransferase
YP_789630.1|gi|116051534|	13	1277	1,72E-01	2,37E-01	1,63	0,25	bacA mutant	WT	PA14_18630	serine protease
YP_789632.1|gi|116051532|	2	90	5,46E-01	4,54E-01	1,24	0,08	WT	bacA mutant	PA14_18650	hypothetical protein
YP_789634.1|gi|116051530|	7	308	9,26E-01	5,65E-01	1,00	0,05	WT	bacA mutant	PA14_18670	bacterioferritin
YP_789636.1|gi|116051528|	16	1051	1,15E-02	6,69E-02	1,99	0,90	WT	bacA mutant	PA14_18690	peroxidase
YP_789638.1|gi|116051526|	5	359	3,95E-02	1,13E-01	1,34	0,62	WT	bacA mutant	PA14_18710	dihydroorotase
YP_789640.1|gi|116051524|	15	979	4,31E-02	1,16E-01	1,10	0,60	WT	bacA mutant	PA14_18740	argininosuccinate synthase
YP_789641.1|gi|116051523|	2	78	6,83E-03	5,76E-02	1,61	0,96	WT	bacA mutant	PA14_18750	lactoylglutathione lyase
YP_789662.1|gi|116051503|	2	52	4,96E-01	4,33E-01	1,14	0,09	WT	bacA mutant	PA14_19010	hypothetical protein
YP_789664.1|gi|116051501|	7	395	8,92E-01	5,58E-01	1,10	0,05	WT	bacA mutant	PA14_19030	hypothetical protein
YP_789665.1|gi|116051500|	16	968	1,15E-01	1,92E-01	1,15	0,34	WT	bacA mutant	PA14_19050	methionyl-tRNA synthetase
YP_789666.1|gi|116054449|	10	539	4,70E-03	4,75E-02	1,36	0,98	WT	bacA mutant	PA14_19065	hypothetical protein
YP_789668.1|gi|116051498|	3	129	7,00E-01	5,14E-01	1,07	0,06	WT	bacA mutant	PA14_19100	rhamnosyltransferase chain A
YP_789670.1|gi|116051496|	8	362	6,27E-01	4,86E-01	1,04	0,07	WT	bacA mutant	PA14_19120	transcriptional regulator RhlR
YP_789672.1|gi|116051494|	2	124	1,93E-02	8,45E-02	1,37	0,81	WT	bacA mutant	PA14_19140	cyclohexadienyl dehydratase
YP_789676.1|gi|116051490|	10	855	2,57E-02	9,61E-02	1,20	0,74	WT	bacA mutant	PA14_19190	malate dehydrogenase
YP_789678.1|gi|116051489|	2	38	1,81E-01	2,46E-01	1,29	0,24	WT	bacA mutant	PA14_19210	hypothetical protein
YP_789679.1|gi|116051488|	2	39	3,63E-01	3,69E-01	1,41	0,13	bacA mutant	WT	PA14_19230	hypothetical protein
YP_789681.1|gi|116051486|	5	252	3,23E-02	1,04E-01	1,41	0,68	bacA mutant	WT	PA14_19290	ATP-dependent RNA helicase
YP_789682.1|gi|116051485|	2	83	9,54E-01	5,72E-01	1,48	0,05	WT	bacA mutant	PA14_19310	hypothetical protein
YP_789686.1|gi|116051481|	9	670	2,00E-03	3,96E-02	2,26	1,00	WT	bacA mutant	PA14_19350	hypothetical protein

YP_789687.1|gi|116051480|	13	713	2,42E-02	9,38E-02	1,46	0,75	WT	bacA mutant	PA14_19360	GNAT family acetyltransferase
YP_789688.1|gi|116051479|	16	921	4,05E-03	4,75E-02	1,69	0,99	WT	bacA mutant	PA14_19370	asparagine synthetase
YP_789689.1|gi|116051478|	2	91	4,01E-01	3,87E-01	1,24	0,11	bacA mutant	WT	PA14_19380	transcriptional regulator
YP_789691.1|gi|116051476|	2	111	3,88E-01	3,81E-01	1,15	0,12	bacA mutant	WT	PA14_19400	5-methylaminomethyl-2-thiouridine methyltransferase
YP_789692.1|gi|116051475|	12	643	1,37E-01	2,12E-01	1,22	0,30	bacA mutant	WT	PA14_19410	hypothetical protein
YP_789708.1|gi|116051459|	2	145	1,54E-01	2,24E-01	1,61	0,27	WT	bacA mutant	PA14_19610	hypothetical protein
YP_789713.1|gi|116051454|	5	253	1,22E-01	1,98E-01	1,40	0,32	WT	bacA mutant	PA14_19660	flavodoxin
YP_789720.1|gi|116051447|	2	102	3,55E-03	4,75E-02	1,50	0,99	WT	bacA mutant	PA14_19730	oxidoreductase
YP_789721.1|gi|116051446|	3	114	4,89E-02	1,20E-01	1,18	0,56	WT	bacA mutant	PA14_19740	enoyl-CoA hydratase
YP_789728.1|gi|116051439|	3	106	1,20E-01	1,97E-01	1,24	0,33	WT	bacA mutant	PA14_19860	hypothetical protein
YP_789729.1|gi|116051438|	10	668	1,51E-02	7,49E-02	1,43	0,86	WT	bacA mutant	PA14_19870	leucine dehydrogenase
YP_789730.1|gi|116051437|	10	621	1,05E-02	6,67E-02	1,72	0,92	WT	bacA mutant	PA14_19900	pyruvate dehydrogenase E1 component subunit alpha
YP_789731.1|gi|116051436|	5	248	4,38E-03	4,75E-02	2,10	0,99	WT	bacA mutant	PA14_19910	pyruvate dehydrogenase E1 component, beta chain
YP_789732.1|gi|116051435|	3	208	2,73E-02	9,79E-02	1,72	0,72	WT	bacA mutant	PA14_19920	branched-chain alpha-keto acid dehydrogenase subunit E2
YP_789747.1|gi|116051420|	3	110	5,46E-01	4,54E-01	1,11	0,08	bacA mutant	WT	PA14_20080	hypothetical protein
YP_789752.1|gi|116051415|	7	287	1,55E-01	2,25E-01	1,31	0,27	WT	bacA mutant	PA14_20140	ferredoxin--NADP+ reductase
YP_789757.1|gi|116051410|	6	212	1,02E-02	6,67E-02	1,48	0,92	bacA mutant	WT	PA14_20200	nitrous-oxide reductase
YP_789764.1|gi|116051403|	4	190	4,18E-01	3,97E-01	1,24	0,11	WT	bacA mutant	PA14_20290	DNA binding-protein
YP_789786.1|gi|116051381|	15	1049	4,34E-03	4,75E-02	1,66	0,99	WT	bacA mutant	PA14_20560	acylamide amidohydrolase
YP_789795.1|gi|116051372|	6	254	3,60E-02	1,08E-01	1,10	0,65	bacA mutant	WT	PA14_20650	D-serine dehydratase
YP_789796.1|gi|116051371|	5	492	5,35E-03	5,00E-02	1,14	0,98	bacA mutant	WT	PA14_20670	glutamine synthetase
YP_789798.1|gi|116051369|	2	148	5,37E-02	1,28E-01	2,52	0,54	WT	bacA mutant	PA14_20690	hypothetical protein
YP_789803.1|gi|116051364|	10	728	5,80E-01	4,66E-01	1,05	0,08	WT	bacA mutant	PA14_20750	chemotaxis protein
YP_789804.1|gi|116051363|	3	131	6,51E-01	4,95E-01	1,12	0,07	WT	bacA mutant	PA14_20760	chemotaxis protein methyltransferase
YP_789805.1|gi|116051362|	3	213	8,45E-01	5,52E-01	1,02	0,05	WT	bacA mutant	PA14_20770	hypothetical protein
YP_789806.1|gi|116051361|	3	145	6,94E-01	5,11E-01	1,12	0,06	WT	bacA mutant	PA14_20780	two-component response regulator

YP_789811.1|gi|116051356|	3	99	7,99E-02	1,56E-01	1,26	0,43	WT	bacA mutant	PA14_20850	MarR family transcriptional regulator
YP_789813.1|gi|116051354|	3	123	2,68E-01	3,14E-01	1,38	0,17	bacA mutant	WT	PA14_20870	hypothetical protein
YP_789815.1|gi|116051352|	5	500	8,88E-01	5,57E-01	1,02	0,05	WT	bacA mutant	PA14_20890	ADP-L-glycero-D-manno-heptose-6-epimerase
YP_789819.1|gi|116051348|	6	366	9,45E-01	5,72E-01	1,02	0,05	WT	bacA mutant	PA14_20950	3-oxoacyl-ACP synthase
YP_789820.1|gi|116051347|	3	131	4,30E-02	1,16E-01	1,69	0,60	WT	bacA mutant	PA14_20960	isomerase
YP_789821.1|gi|116051346|	7	341	7,14E-01	5,18E-01	1,09	0,06	WT	bacA mutant	PA14_20970	cytochrome P450
YP_789822.1|gi|116051345|	5	257	1,22E-01	1,98E-01	1,25	0,32	WT	bacA mutant	PA14_20980	short chain dehydrogenas
YP_789823.1|gi|116051344|	7	370	2,17E-01	2,76E-01	1,32	0,21	WT	bacA mutant	PA14_21000	hypothetical protein
YP_789824.1|gi|116051343|	4	163	6,25E-01	4,86E-01	1,14	0,07	bacA mutant	WT	PA14_21010	FAD-dependent monooxygenase
YP_789825.1|gi|116051342|	2	73	6,60E-01	4,98E-01	1,06	0,07	WT	bacA mutant	PA14_21020	non-ribosomal peptide synthetase
YP_789826.1|gi|116051341|	9	635	4,76E-02	1,20E-01	1,34	0,57	WT	bacA mutant	PA14_21030	ATP-dependent Clp protease proteolytic subunit
YP_789839.1|gi|116054457|	12	700	1,04E-01	1,83E-01	1,37	0,36	WT	bacA mutant	PA14_21175	hypothetical protein
YP_789843.1|gi|116051325|	8	631	3,31E-02	1,06E-01	1,71	0,67	WT	bacA mutant	PA14_21220	hypothetical protein
YP_789851.1|gi|116051317|	2	119	4,76E-02	1,20E-01	1,87	0,57	WT	bacA mutant	PA14_21310	hypothetical protein
YP_789852.1|gi|116051316|	2	92	5,70E-01	4,61E-01	1,11	0,08	bacA mutant	WT	PA14_21320	hypothetical protein
YP_789853.1|gi|116051315|	6	410	1,48E-01	2,19E-01	1,73	0,28	WT	bacA mutant	PA14_21340	long-chain-fatty-acid--CoA ligase
YP_789854.1|gi|116051314|	14	657	3,52E-01	3,64E-01	1,50	0,13	WT	bacA mutant	PA14_21370	long-chain-fatty-acid--CoA ligase
YP_789858.1|gi|116051310|	3	106	6,55E-02	1,40E-01	1,60	0,48	WT	bacA mutant	PA14_21440	HIT family protein
YP_789864.1|gi|116051304|	2	97	4,47E-01	4,12E-01	1,18	0,10	WT	bacA mutant	PA14_21510	hypothetical protein
YP_789867.1|gi|116051301|	10	524	1,40E-01	2,15E-01	1,10	0,29	WT	bacA mutant	PA14_21540	3-oxoacyl-ACP synthase
YP_789884.1|gi|116051284|	3	179	6,88E-02	1,44E-01	1,24	0,47	WT	bacA mutant	PA14_21710	hypothetical protein
YP_789886.1|gi|116051282|	2	119	7,08E-02	1,45E-01	2,84	0,46	bacA mutant	WT	PA14_21730	TonB-dependent receptor
YP_789891.1|gi|116051277|	7	370	2,31E-01	2,87E-01	1,18	0,19	bacA mutant	WT	PA14_21790	recombination associated protein
YP_789892.1|gi|116051276|	13	880	1,25E-01	2,01E-01	1,26	0,32	WT	bacA mutant	PA14_21820	peptidyl-prolyl cis-trans isomerase, FkbP-type
YP_789898.1|gi|116051270|	6	303	1,14E-01	1,92E-01	1,30	0,34	WT	bacA mutant	PA14_21880	periplasmic tail-specific protease
YP_789899.1|gi|116051269|	8	488	4,89E-02	1,20E-01	1,17	0,56	WT	bacA mutant	PA14_21890	oxidoreductase

YP_789905.1|gi|116051263|	5	208	3,33E-02	1,06E-01	1,66	0,67	WT	bacA mutant	PA14_21960	hypothetical protein
YP_789908.1|gi|116051260|	7	401	7,48E-02	1,49E-01	1,22	0,45	bacA mutant	WT	PA14_21990	aminopeptidase 2
YP_789910.1|gi|116051258|	2	59	5,17E-01	4,43E-01	1,20	0,09	WT	bacA mutant	PA14_22010	cell division topological specificity factor MinE
YP_789911.1|gi|116051257|	11	844	4,10E-03	4,75E-02	1,46	0,99	WT	bacA mutant	PA14_22020	cell division inhibitor MinD
YP_789912.1|gi|116051256|	3	153	6,79E-01	5,06E-01	1,19	0,06	WT	bacA mutant	PA14_22040	septum formation inhibitor
YP_789932.1|gi|116051237|	2	76	3,10E-01	3,42E-01	1,30	0,15	bacA mutant	WT	PA14_22270	recombinase
YP_789934.1|gi|116051235|	4	151	8,53E-03	6,03E-02	1,25	0,94	WT	bacA mutant	PA14_22290	hypothetical protein
YP_789935.1|gi|116051234|	3	212	1,36E-01	2,11E-01	1,25	0,30	WT	bacA mutant	PA14_22310	hypothetical protein
YP_789946.1|gi|116051223|	2	99	5,81E-02	1,33E-01	1,52	0,52	WT	bacA mutant	PA14_22450	peptidyl-prolyl cis-trans isomerase A
YP_789947.1|gi|116051222|	2	101	5,38E-02	1,28E-01	2,06	0,54	bacA mutant	WT	PA14_22460	alpha/beta hydrolase
YP_789948.1|gi|116051221|	6	268	3,39E-02	1,06E-01	1,27	0,66	WT	bacA mutant	PA14_22470	LysR family transcriptional regulator
YP_789965.1|gi|116051204|	3	145	1,13E-01	1,91E-01	1,22	0,34	WT	bacA mutant	PA14_22650	ABC transporter
YP_789966.1|gi|116051203|	7	366	2,46E-02	9,43E-02	1,31	0,75	bacA mutant	WT	PA14_22660	hypothetical protein
YP_789971.1|gi|116051198|	5	191	2,46E-01	2,99E-01	1,20	0,18	WT	bacA mutant	PA14_22710	hypothetical protein
YP_789975.1|gi|116051194|	2	97	5,46E-02	1,28E-01	1,53	0,53	WT	bacA mutant	PA14_22760	two-component response regulator
YP_789980.1|gi|116051189|	3	127	3,56E-03	4,75E-02	1,28	0,99	WT	bacA mutant	PA14_22830	SUA5/yciO/yrdC family:Sua5/YciO/YrdC/YwlC family protein
YP_789985.1|gi|116051184|	9	583	2,18E-03	4,01E-02	1,65	1,00	bacA mutant	WT	PA14_22890	glyceraldehyde-3-phosphate dehydrogenase
YP_789986.1|gi|116051183|	17	1151	3,74E-01	3,73E-01	1,13	0,12	bacA mutant	WT	PA14_22910	phosphogluconate dehydratase
YP_789987.1|gi|116051182|	3	243	8,09E-01	5,44E-01	1,01	0,05	bacA mutant	WT	PA14_22930	glucokinase
YP_789990.1|gi|116051179|	10	610	6,18E-02	1,36E-01	1,41	0,50	WT	bacA mutant	PA14_22980	sugar ABC transporter substrate-binding protein
YP_789991.1|gi|116051178|	2	110	2,12E-01	2,72E-01	1,93	0,21	bacA mutant	WT	PA14_22990	ABC sugar transporter permease
YP_789993.1|gi|116051176|	12	780	1,99E-01	2,60E-01	1,14	0,22	bacA mutant	WT	PA14_23010	ABC transporter ATP-binding protein
YP_789994.1|gi|116051175|	19	1279	9,43E-01	5,71E-01	1,07	0,05	bacA mutant	WT	PA14_23030	glucose/carbohydrate outer membrane porin OprB precursor
YP_789997.1|gi|116051172|	4	186	2,47E-01	2,99E-01	1,24	0,18	bacA mutant	WT	PA14_23070	glucose-6-phosphate 1-dehydrogenase
YP_789999.1|gi|116051170|	2	92	6,03E-02	1,35E-01	1,29	0,50	bacA mutant	WT	PA14_23090	keto-hydroxyglutarate-aldolase/keto-deoxy- phosphogluconate aldolase
YP_790001.1|gi|116051168|	9	437	3,25E-01	3,51E-01	1,21	0,14	WT	bacA mutant	PA14_23110	hypothetical protein

YP_790007.1|gi|116051162|	9	620	4,87E-01	4,30E-01	1,05	0,09	WT	bacA mutant	PA14_23200	short chain dehydrogenase
YP_790008.1|gi|116051161|	3	120	4,04E-03	4,75E-02	1,44	0,99	WT	bacA mutant	PA14_23210	phosphoglycolate phosphatase
YP_790009.1|gi|116051160|	7	483	4,71E-03	4,75E-02	1,46	0,98	WT	bacA mutant	PA14_23220	3-demethylubiquinone-9 3-methyltransferase
YP_790010.1|gi|116051159|	3	210	5,67E-01	4,60E-01	1,04	0,08	WT	bacA mutant	PA14_23240	N-ethylammeline chlorohydrolase
YP_790012.1|gi|116051157|	12	665	1,56E-01	2,25E-01	1,18	0,27	WT	bacA mutant	PA14_23260	DNA gyrase subunit A
YP_790013.1|gi|116051156|	10	693	2,87E-01	3,29E-01	1,07	0,16	WT	bacA mutant	PA14_23270	phosphoserine aminotransferase
YP_790014.1|gi|116051155|	8	434	2,82E-02	9,88E-02	1,10	0,71	WT	bacA mutant	PA14_23280	chorismate mutase
YP_790016.1|gi|116051153|	4	341	1,14E-02	6,67E-02	1,39	0,90	WT	bacA mutant	PA14_23310	bifunctional cyclohexadienyl dehydrogenase/ 3-phosphoshikimate 1-carboxyvinyltransferase
YP_790017.1|gi|116051152|	2	133	9,91E-01	5,83E-01	1,00	0,05	bacA mutant	WT	PA14_23320	cytidylate kinase
YP_790018.1|gi|116051151|	17	1098	9,71E-02	1,76E-01	1,16	0,38	bacA mutant	WT	PA14_23330	30S ribosomal protein S1
YP_790022.1|gi|116051147|	23	1635	5,64E-01	4,60E-01	1,03	0,08	WT	bacA mutant	PA14_23370	UDP-N-acetylglucosamine 2-epimerase
YP_790023.1|gi|116051146|	19	1217	1,89E-01	2,53E-01	1,07	0,23	WT	bacA mutant	PA14_23380	UDP-N-acetyl-D-mannosaminuronate dehydrogenase
YP_790027.1|gi|116051142|	16	913	8,80E-01	5,56E-01	1,00	0,05	bacA mutant	WT	PA14_23420	zinc-binding dehydrogenase
YP_790028.1|gi|116051141|	3	148	4,89E-01	4,30E-01	1,19	0,09	bacA mutant	WT	PA14_23430	heparinase
YP_790029.1|gi|116051140|	7	246	3,67E-01	3,71E-01	1,35	0,12	bacA mutant	WT	PA14_23440	group 1 glycosyl transferase
YP_790030.1|gi|116051139|	5	236	5,80E-01	4,66E-01	1,09	0,08	bacA mutant	WT	PA14_23450	NAD dependent epimerase/dehydratase
YP_790032.1|gi|116051137|	5	298	7,73E-01	5,36E-01	1,04	0,06	bacA mutant	WT	PA14_23470	nucleotide sugar epimerase/dehydratase WbpM
YP_790034.1|gi|116051135|	18	1346	9,76E-01	5,78E-01	1,00	0,05	WT	bacA mutant	PA14_23500	aromatic amino acid aminotransferase
YP_790035.1|gi|116051134|	3	89	8,34E-01	5,50E-01	1,02	0,05	WT	bacA mutant	PA14_23510	excinuclease ABC subunit B
YP_790039.1|gi|116051130|	8	374	7,03E-01	5,16E-01	1,03	0,06	bacA mutant	WT	PA14_23560	glutamyl-tRNA synthetase
YP_790042.1|gi|116051127|	3	158	7,91E-01	5,39E-01	1,15	0,06	bacA mutant	WT	PA14_23620	aldolase
YP_790043.1|gi|116051126|	2	98	3,10E-01	3,42E-01	1,23	0,15	bacA mutant	WT	PA14_23630	hypothetical protein
YP_790046.1|gi|116051123|	3	169	4,61E-01	4,17E-01	1,08	0,10	WT	bacA mutant	PA14_23670	hypothetical protein
YP_790047.1|gi|116051122|	9	587	1,95E-01	2,57E-01	1,28	0,23	WT	bacA mutant	PA14_23680	heat-shock protein IbpA
YP_790049.1|gi|116051120|	3	88	5,31E-01	4,48E-01	1,21	0,08	WT	bacA mutant	PA14_23700	LysR family transcriptional regulator
YP_790050.1|gi|116051119|	7	488	1,06E-01	1,84E-01	1,53	0,36	WT	bacA mutant	PA14_23720	translation initiation inhibitor

YP_790055.1|gi|116051114|	10	560	5,46E-02	1,28E-01	1,16	0,53	WT	bacA mutant	PA14_23790	3-isopropylmalate dehydrogenase
YP_790056.1|gi|116051113|	13	866	2,25E-01	2,81E-01	1,10	0,20	bacA mutant	WT	PA14_23800	aspartate-semialdehyde dehydrogenase
YP_790058.1|gi|116051111|	14	780	3,24E-02	1,05E-01	1,41	0,68	WT	bacA mutant	PA14_23830	pilus assembly protein
YP_790059.1|gi|116051110|	2	108	4,52E-03	4,75E-02	1,44	0,99	bacA mutant	WT	PA14_23840	tRNA pseudouridine synthase A
YP_790061.1|gi|116051108|	3	142	1,14E-01	1,92E-01	1,17	0,34	WT	bacA mutant	PA14_23860	acetyl-CoA carboxylase subunit beta
YP_790062.1|gi|116051107|	2	145	7,43E-01	5,27E-01	1,15	0,06	WT	bacA mutant	PA14_23880	folylpolyglutamate synthetase
YP_790063.1|gi|116051106|	3	142	6,67E-01	5,02E-01	1,07	0,07	WT	bacA mutant	PA14_23890	hypothetical protein
YP_790065.1|gi|116051104|	19	1150	5,61E-01	4,59E-01	1,07	0,08	WT	bacA mutant	PA14_23920	amidophosphoribosyltransferase
YP_790066.1|gi|116051103|	9	478	8,33E-02	1,60E-01	1,28	0,42	WT	bacA mutant	PA14_23930	O-succinylhomoserine sulfhydrylase
YP_790068.1|gi|116051101|; YP_791371.1|gi|116049822|	22	1759	6,12E-01	4,80E-01	1,12	0,07	bacA mutant	WT	PA14_23970	general secretion pathway protein D
YP_790070.1|gi|116051099|	5	161	4,87E-01	4,30E-01	1,11	0,09	bacA mutant	WT	PA14_23990	general secretion pathway protein E
YP_790072.1|gi|116051097|	3	154	1,40E-01	2,14E-01	1,22	0,29	WT	bacA mutant	PA14_24020	general secretion pathway protein G
YP_790077.1|gi|116051092|	2	73	6,77E-01	5,05E-01	1,07	0,06	bacA mutant	WT	PA14_24080	general secretion pathway protein L
YP_790081.1|gi|116051088|	2	51	5,72E-01	4,62E-01	1,15	0,08	WT	bacA mutant	PA14_24170	2,4-dienoyl-CoA reductase
YP_790085.1|gi|116051084|	3	107	1,67E-01	2,33E-01	1,19	0,26	WT	bacA mutant	PA14_24220	inorganic polyphosphate/ATP-NAD kinase
YP_790090.1|gi|116051080|	19	1035	1,40E-01	2,14E-01	1,62	0,29	WT	bacA mutant	PA14_24270	aminopeptidase
YP_790091.1|gi|116051079|	7	435	4,89E-01	4,30E-01	1,26	0,09	bacA mutant	WT	PA14_24290	glycine betaine transmethylase
YP_790092.1|gi|116051078|	9	461	7,20E-01	5,19E-01	1,03	0,06	WT	bacA mutant	PA14_24300	hypothetical protein
YP_790093.1|gi|116051077|	4	245	2,24E-04	2,46E-02	1,72	1,00	WT	bacA mutant	PA14_24310	BNR/Asp-box repeat-containing protein
YP_790104.1|gi|116051066|	3	128	1,16E-01	1,93E-01	1,16	0,34	WT	bacA mutant	PA14_24430	hypothetical protein
YP_790106.1|gi|116054423|	27	1746	2,13E-01	2,73E-01	1,55	0,21	WT	bacA mutant	PA14_24445	NAD-dependent glutamate dehydrogenase
YP_790108.1|gi|116051063|	4	153	7,93E-04	2,86E-02	3,46	1,00	WT	bacA mutant	PA14_24490	hypothetical protein
YP_790109.1|gi|116051062|	5	288	2,90E-01	3,31E-01	1,14	0,16	WT	bacA mutant	PA14_24500	lipoprotein
YP_790110.1|gi|116051061|	3	141	6,17E-01	4,82E-01	1,17	0,07	WT	bacA mutant	PA14_24510	hypothetical protein
YP_790115.1|gi|116051056|; YP_790116.1|gi|116051055|	4	161	5,18E-01	4,43E-01	1,16	0,09	bacA mutant	WT	PA14_24580	hypothetical protein

YP_790118.1|gi|116051053|	2	123	5,87E-01	4,68E-01	1,10	0,07	WT	bacA mutant	PA14_24610	hydrolytic enzyme
YP_790119.1|gi|116051051|	2	84	2,46E-02	9,43E-02	1,26	0,75	bacA mutant	WT	PA14_24620	hypothetical protein
YP_790121.1|gi|116051050|	6	317	1,48E-01	2,19E-01	1,16	0,28	WT	bacA mutant	PA14_24640	dihydroorotate dehydrogenase 2
YP_790124.1|gi|116054427|	3	172	8,60E-01	5,53E-01	1,01	0,05	bacA mutant	WT	PA14_24675	hypothetical protein
YP_790129.1|gi|116051044|	2	56	7,87E-01	5,39E-01	1,13	0,06	WT	bacA mutant	PA14_24730	deoxyguanosinetriphosphate triphosphohydrolase-like protein
YP_790130.1|gi|116051043|	3	161	1,89E-01	2,52E-01	1,55	0,23	bacA mutant	WT	PA14_24740	hypothetical protein
YP_790131.1|gi|116051042|	2	59	7,32E-01	5,23E-01	1,15	0,06	WT	bacA mutant	PA14_24760	hypothetical protein
YP_790132.1|gi|116051041|	3	375	9,00E-01	5,59E-01	1,05	0,05	WT	bacA mutant	PA14_24770	hypothetical protein
YP_790134.1|gi|116051039|	14	905	8,27E-01	5,49E-01	1,05	0,05	WT	bacA mutant	PA14_24790	outer membrane porin
YP_790141.1|gi|116051032|	2	268	7,45E-02	1,49E-01	1,25	0,45	WT	bacA mutant	PA14_24880	lipoprotein
YP_790143.1|gi|116051030|	7	659	8,53E-01	5,53E-01	1,01	0,05	WT	bacA mutant	PA14_24900	molybdopterin biosynthetic protein B2
YP_790144.1|gi|116051029|	4	208	7,86E-01	5,39E-01	1,03	0,06	bacA mutant	WT	PA14_24910	molybdenum cofactor biosynthesis protein A2
YP_790146.1|gi|116051027|	5	192	8,97E-01	5,59E-01	1,10	0,05	WT	bacA mutant	PA14_24940	hypothetical protein
YP_790149.1|gi|116051024|	6	299	7,16E-01	5,18E-01	1,03	0,06	WT	bacA mutant	PA14_24970	lipid kinase
YP_790150.1|gi|116051023|	7	278	3,34E-01	3,54E-01	1,12	0,14	WT	bacA mutant	PA14_24980	hypothetical protein
YP_790153.1|gi|116051020|	4	193	2,06E-01	2,67E-01	1,17	0,22	bacA mutant	WT	PA14_25020	ABC transporter ATP-binding protein
YP_790155.1|gi|116051018|	3	165	4,12E-02	1,16E-01	1,61	0,61	WT	bacA mutant	PA14_25040	hypothetical protein
YP_790158.1|gi|116051015|	31	2110	5,24E-01	4,46E-01	1,12	0,08	WT	bacA mutant	PA14_25080	multifunctional fatty acid oxidation complex subunit alpha
YP_790159.1|gi|116051014|	14	788	2,45E-03	4,16E-02	2,07	1,00	WT	bacA mutant	PA14_25090	3-ketoacyl-CoA thiolase
YP_790161.1|gi|116051012|	7	330	8,60E-01	5,53E-01	1,03	0,05	bacA mutant	WT	PA14_25110	DNA topoisomerase I
YP_790162.1|gi|116051011|	2	72	7,81E-01	5,38E-01	1,07	0,06	WT	bacA mutant	PA14_25130	hypothetical protein
YP_790166.1|gi|116051007|	4	176	6,22E-01	4,84E-01	1,08	0,07	bacA mutant	WT	PA14_25180	transcriptional regulator PsrA
YP_790167.1|gi|116054412|	8	346	8,96E-02	1,67E-01	1,15	0,40	WT	bacA mutant	PA14_25195	beta-hexosaminidase
YP_790168.1|gi|116051006|	6	318	8,26E-01	5,48E-01	1,04	0,05	bacA mutant	WT	PA14_25210	5'-methylthioadenosine phosphorylase
YP_790170.1|gi|116051004|	2	56	3,26E-01	3,51E-01	1,61	0,14	WT	bacA mutant	PA14_25230	transcription-repair coupling factor
YP_790171.1|gi|116051003|	23	1912	5,51E-01	4,56E-01	1,08	0,08	WT	bacA mutant	PA14_25250	glyceraldehyde-3-phosphate dehydrogenase

YP_790173.1|gi|116051001|	11	657	6,79E-01	5,06E-01	1,03	0,06	WT	bacA mutant	PA14_25280	Na(+)-translocating NADH-quinone reductase subunit A
YP_790175.1|gi|116051000|	4	234	3,68E-01	3,71E-01	1,43	0,12	WT	bacA mutant	PA14_25320	Na(+)-translocating NADH-quinone reductase subunit C
YP_790178.1|gi|116050997|	5	259	5,31E-01	4,48E-01	1,48	0,08	WT	bacA mutant	PA14_25350	Na(+)-translocating NADH-quinone reductase subunit F
YP_790181.1|gi|116050994|	20	1135	8,00E-01	5,42E-01	1,02	0,06	WT	bacA mutant	PA14_25390	soluble pyridine nucleotide transhydrogenase
YP_790182.1|gi|116050993|	3	99	5,80E-03	5,27E-02	1,71	0,97	WT	bacA mutant	PA14_25400	phosphodiesterase
YP_790185.1|gi|116050991|	3	83	8,37E-01	5,50E-01	1,17	0,05	WT	bacA mutant	PA14_25430	hypothetical protein
YP_790186.1|gi|116050989|	2	89	4,34E-02	1,16E-01	1,39	0,60	WT	bacA mutant	PA14_25440	lipoprotein releasing system, ATP-binding protein
YP_790187.1|gi|116050988|	2	67	8,54E-01	5,53E-01	1,25	0,05	WT	bacA mutant	PA14_25450	hypothetical protein
YP_790190.1|gi|116050985|	4	202	8,56E-01	5,53E-01	1,09	0,05	WT	bacA mutant	PA14_25490	tolQ-type transport protein
YP_790194.1|gi|116050981|	4	170	3,07E-01	3,41E-01	1,09	0,15	WT	bacA mutant	PA14_25530	3-deoxy-manno-octulosonate cytidylyltransferase
YP_790197.1|gi|116050978|	23	1529	2,79E-01	3,23E-01	1,20	0,16	bacA mutant	WT	PA14_25560	ribonuclease E
YP_790198.1|gi|116050977|	8	359	8,58E-01	5,53E-01	1,01	0,05	WT	bacA mutant	PA14_25580	ribosomal large subunit pseudouridine synthase C
YP_790200.1|gi|116050975|	5	507	9,40E-01	5,71E-01	1,06	0,05	WT	bacA mutant	PA14_25600	peptidase
YP_790205.1|gi|116050970|	8	506	1,27E-02	6,84E-02	1,42	0,89	bacA mutant	WT	PA14_25650	malonyl-CoA-ACP transacylase
YP_790206.1|gi|116050969|	4	280	4,31E-02	1,16E-01	1,27	0,60	WT	bacA mutant	PA14_25660	3-ketoacyl-ACP reductase
YP_790207.1|gi|116050968|	3	202	2,17E-02	9,13E-02	1,69	0,78	WT	bacA mutant	PA14_25670	acyl carrier protein
YP_790208.1|gi|116050967|	6	270	3,04E-01	3,39E-01	1,08	0,15	WT	bacA mutant	PA14_25690	3-oxoacyl-ACP synthase
YP_790211.1|gi|116050964|	2	131	7,90E-01	5,39E-01	1,04	0,06	WT	bacA mutant	PA14_25740	thymidylate kinase
YP_790214.1|gi|116050961|	3	107	7,45E-01	5,27E-01	1,04	0,06	bacA mutant	WT	PA14_25780	TatD family deoxyribonuclease
YP_790215.1|gi|116050960|	3	180	3,57E-01	3,66E-01	1,24	0,13	WT	bacA mutant	PA14_25790	hypothetical protein
YP_790216.1|gi|116050959|	5	185	2,74E-02	9,79E-02	1,46	0,72	WT	bacA mutant	PA14_25800	TetR family transcriptional regulator
YP_790218.1|gi|116050957|	4	177	5,22E-01	4,45E-01	1,09	0,09	bacA mutant	WT	PA14_25820	lipoprotein
YP_790220.1|gi|116050955|	20	1127	3,21E-04	2,46E-02	1,47	1,00	WT	bacA mutant	PA14_25840	electron transfer flavoprotein-ubiquinone oxidoreductase
YP_790221.1|gi|116050954|	14	1078	6,47E-02	1,40E-01	1,29	0,49	WT	bacA mutant	PA14_25860	electron transfer flavoprotein subunit beta
YP_790222.1|gi|116050953|	14	1251	1,59E-02	7,69E-02	1,66	0,85	WT	bacA mutant	PA14_25880	electron transfer flavoprotein subunit alpha
YP_790223.1|gi|116050952|	7	358	1,42E-02	7,23E-02	1,24	0,87	WT	bacA mutant	PA14_25900	trans-2-enoyl-CoA reductase

YP_790228.1|gi|116050947|	6	484	2,38E-02	9,30E-02	1,41	0,76	bacA mutant	WT	PA14_25960	cobalamin biosynthesis protein cobW
YP_790231.1|gi|116050944|	2	60	9,42E-01	5,71E-01	1,01	0,05	WT	bacA mutant	PA14_25990	magnesium chelatase
YP_790234.1|gi|116050941|	14	899	1,84E-02	8,13E-02	1,35	0,82	WT	bacA mutant	PA14_26020	aminopeptidase
YP_790246.1|gi|116050930|	15	739	3,33E-02	1,06E-01	1,48	0,67	WT	bacA mutant	PA14_26190	hypothetical protein
YP_790254.1|gi|116050922|; YP_790674.1|gi|116050507|	9	665	5,52E-01	4,56E-01	1,31	0,08	WT	bacA mutant	PA14_26280	chemotaxis transducer
YP_790259.1|gi|116050917|	7	532	3,90E-02	1,13E-01	1,30	0,63	WT	bacA mutant	PA14_26350	hypothetical protein
YP_790272.1|gi|116050905|	4	150	2,03E-03	3,96E-02	2,16	1,00	WT	bacA mutant	PA14_26540	hypothetical protein
YP_790274.1|gi|116050903|	4	142	1,67E-02	7,85E-02	1,63	0,84	WT	bacA mutant	PA14_26560	outer membrane protein
YP_790275.1|gi|116050902|	2	76	8,12E-01	5,45E-01	1,02	0,05	WT	bacA mutant	PA14_26570	transcriptional regulator
YP_790277.1|gi|116050900|	2	74	6,11E-01	4,80E-01	1,09	0,07	WT	bacA mutant	PA14_26590	GntR family transcriptional regulator
YP_790285.1|gi|116050892|	5	116	1,51E-03	3,69E-02	2,40	1,00	WT	bacA mutant	PA14_26700	acyl-CoA dehydrogenase
YP_790288.1|gi|116050889|	2	41	1,21E-01	1,98E-01	1,87	0,33	WT	bacA mutant	PA14_26750	hypothetical protein
YP_790291.1|gi|116050886|	2	91	5,00E-01	4,34E-01	1,17	0,09	bacA mutant	WT	PA14_26780	hypothetical protein
YP_790299.1|gi|116050878|	4	171	5,97E-01	4,72E-01	1,07	0,07	bacA mutant	WT	PA14_26910	hypothetical protein
YP_790302.1|gi|116050875|	2	138	9,93E-01	5,83E-01	1,03	0,05	WT	bacA mutant	PA14_26940	hypothetical protein
YP_790307.1|gi|116050870|	3	154	5,79E-01	4,66E-01	1,13	0,08	bacA mutant	WT	PA14_27000	chemotaxis transducer
YP_790310.1|gi|116050867|	3	192	6,92E-01	5,11E-01	1,49	0,06	WT	bacA mutant	PA14_27070	hypothetical protein
YP_790315.1|gi|116050862|	2	68	1,87E-01	2,51E-01	1,37	0,23	bacA mutant	WT	PA14_27130	transcription elongation factor GreB
YP_790317.1|gi|116050860|	2	105	3,76E-04	2,46E-02	1,49	1,00	WT	bacA mutant	PA14_27150	ABC transporter ATP-binding protein
YP_790320.1|gi|116050857|	2	53	2,36E-02	9,30E-02	1,62	0,76	WT	bacA mutant	PA14_27180	hypothetical protein
YP_790322.1|gi|116050855|	5	355	1,35E-01	2,10E-01	1,35	0,30	WT	bacA mutant	PA14_27210	elongation factor P
YP_790333.1|gi|116050844|	12	781	2,32E-02	9,26E-02	1,89	0,76	bacA mutant	WT	PA14_27370	ATP-dependent RNA helicase
YP_790342.1|gi|116050835|	4	214	3,81E-01	3,77E-01	1,08	0,12	WT	bacA mutant	PA14_27470	zinc carboxypeptidase
YP_790343.1|gi|116050834|	6	583	7,46E-01	5,27E-01	1,30	0,06	WT	bacA mutant	PA14_27480	heat shock protein HtpX
YP_790345.1|gi|116050832|	3	171	3,69E-01	3,72E-01	1,11	0,12	bacA mutant	WT	PA14_27500	aminotransferase

YP_790350.1|gi|116050827|	7	339	1,18E-01	1,95E-01	1,12	0,33	bacA mutant	WT	PA14_27560	hypothetical protein
YP_790352.1|gi|116050825|	2	86	1,70E-02	7,88E-02	1,86	0,83	WT	bacA mutant	PA14_27580	glutathione S-transferase
YP_790358.1|gi|116054398|	2	60	3,50E-01	3,63E-01	1,57	0,13	WT	bacA mutant	PA14_27675	hypothetical protein
YP_790362.1|gi|116050816|	2	144	5,68E-01	4,60E-01	1,12	0,08	bacA mutant	WT	PA14_27710	hypothetical protein
YP_790364.1|gi|116050814|	25	1726	7,70E-01	5,35E-01	1,23	0,06	WT	bacA mutant	PA14_27730	acyl-CoA dehydrogenase
YP_790366.1|gi|116054440|	3	185	8,30E-01	5,49E-01	1,08	0,05	bacA mutant	WT	PA14_27755	glutathione S-transferase
YP_790367.1|gi|116050812|	2	120	1,32E-01	2,09E-01	1,33	0,31	WT	bacA mutant	PA14_27770	ABC transporter ATP-binding protein
YP_790373.1|gi|116050806|	3	188	1,33E-01	2,09E-01	1,16	0,30	WT	bacA mutant	PA14_27850	7-cyano-7-deazaguanine reductase
YP_790379.1|gi|116050800|	9	513	6,35E-01	4,88E-01	1,11	0,07	WT	bacA mutant	PA14_27920	hypothetical protein
YP_790381.1|gi|116050798|	9	667	9,76E-01	5,78E-01	1,04	0,05	WT	bacA mutant	PA14_27940	two-component response regulator
YP_790382.1|gi|116050797|	2	174	6,20E-01	4,83E-01	1,09	0,07	WT	bacA mutant	PA14_27950	hypothetical protein
YP_790383.1|gi|116050796|	19	1510	8,58E-01	5,53E-01	1,01	0,05	bacA mutant	WT	PA14_27960	transaldolase B
YP_790391.1|gi|116050788|	11	756	6,30E-01	4,86E-01	1,12	0,07	WT	bacA mutant	PA14_28050	chemotaxis transducer
YP_790392.1|gi|116050787|	2	98	3,72E-01	3,73E-01	1,21	0,12	WT	bacA mutant	PA14_28060	glutamate carboxypeptidase
YP_790403.1|gi|116050776|	8	376	1,25E-02	6,84E-02	1,47	0,89	WT	bacA mutant	PA14_28180	hypothetical protein
YP_790411.1|gi|116050768|	2	131	7,38E-01	5,25E-01	1,06	0,06	bacA mutant	WT	PA14_28280	PhzF family phenazine biosynthesis protein
YP_790417.1|gi|116050762|	5	290	5,81E-01	4,66E-01	1,11	0,08	WT	bacA mutant	PA14_28340	hypothetical protein
YP_790418.1|gi|116050761|	2	86	8,34E-02	1,60E-01	1,41	0,42	WT	bacA mutant	PA14_28350	hypothetical protein
YP_790423.1|gi|116050756|	16	1475	7,52E-01	5,28E-01	1,15	0,06	bacA mutant	WT	PA14_28400	outer membrane OprD family porin
YP_790427.1|gi|116050752|	2	89	6,12E-01	4,80E-01	1,09	0,07	bacA mutant	WT	PA14_28440	hypothetical protein
YP_790428.1|gi|116050751|	4	232	2,50E-02	9,43E-02	1,82	0,74	WT	bacA mutant	PA14_28450	ecotin
YP_790445.1|gi|116050734|	20	1091	2,64E-01	3,11E-01	1,15	0,17	WT	bacA mutant	PA14_28650	threonyl-tRNA synthetase
YP_790446.1|gi|116050733|	9	709	4,57E-01	4,16E-01	1,21	0,10	bacA mutant	WT	PA14_28660	translation initiation factor IF-3
YP_790449.1|gi|116050730|	12	445	1,38E-01	2,13E-01	1,19	0,30	WT	bacA mutant	PA14_28690	phenylalanyl-tRNA synthetase subunit alpha
YP_790450.1|gi|116050729|	12	844	2,29E-02	9,26E-02	1,70	0,77	bacA mutant	WT	PA14_28710	phenylalanyl-tRNA synthetase subunit beta
YP_790451.1|gi|116050728|	3	153	4,83E-01	4,28E-01	1,15	0,09	WT	bacA mutant	PA14_28720	integration host factor subunit alpha

YP_790452.1|gi|116050727|	2	59	8,86E-01	5,56E-01	1,01	0,05	bacA mutant	WT	PA14_28730	transcriptional regulator
YP_790461.1|gi|116050718|	8	260	7,30E-01	5,22E-01	1,05	0,06	WT	bacA mutant	PA14_28830	hypothetical protein
YP_790468.1|gi|116050712|	6	252	7,83E-03	5,95E-02	1,42	0,95	bacA mutant	WT	PA14_28920	chaperone
YP_790478.1|gi|116050702|	9	576	1,90E-01	2,53E-01	1,18	0,23	WT	bacA mutant	PA14_29020	chloroperoxidase
YP_790486.1|gi|116050694|	11	810	1,28E-01	2,04E-01	1,18	0,31	WT	bacA mutant	PA14_29110	cysteine synthase A
YP_790488.1|gi|116050692|	7	332	4,64E-02	1,20E-01	1,28	0,58	WT	bacA mutant	PA14_29130	ATPase
YP_790490.1|gi|116050690|	2	70	6,92E-01	5,11E-01	1,11	0,06	WT	bacA mutant	PA14_29160	hypothetical protein
YP_790496.1|gi|116050684|	10	478	2,69E-01	3,14E-01	1,15	0,17	WT	bacA mutant	PA14_29230	hypothetical protein
YP_790514.1|gi|116050666|	2	123	4,00E-01	3,87E-01	1,20	0,11	bacA mutant	WT	PA14_29460	quinone oxidoreductase
YP_790527.1|gi|116050654|	4	232	8,82E-01	5,56E-01	1,04	0,05	WT	bacA mutant	PA14_29590	transcriptional regulator
YP_790534.1|gi|116050647|	2	63	4,76E-01	4,25E-01	1,16	0,09	WT	bacA mutant	PA14_29690	hypothetical protein
YP_790542.1|gi|116050639|	6	301	7,77E-01	5,38E-01	1,04	0,06	WT	bacA mutant	PA14_29800	chemotaxis transducer
YP_790546.1|gi|116050635|	2	196	6,51E-01	4,95E-01	1,18	0,07	bacA mutant	WT	PA14_29860	NADH dehydrogenase subunit M
YP_790547.1|gi|116050634|	2	37	5,49E-01	4,54E-01	1,17	0,08	bacA mutant	WT	PA14_29880	NADH dehydrogenase subunit L
YP_790550.1|gi|116050631|	6	277	1,24E-02	6,84E-02	1,63	0,89	WT	bacA mutant	PA14_29920	NADH dehydrogenase subunit I
YP_790551.1|gi|116050630|	3	162	8,44E-01	5,52E-01	1,32	0,05	WT	bacA mutant	PA14_29930	NADH dehydrogenase subunit H
YP_790552.1|gi|116050629|	19	1148	7,57E-01	5,29E-01	1,04	0,06	WT	bacA mutant	PA14_29940	NADH dehydrogenase subunit G
YP_790553.1|gi|116050628|	8	415	4,76E-01	4,25E-01	1,03	0,09	bacA mutant	WT	PA14_29970	NADH dehydrogenase I subunit F
YP_790555.1|gi|116050626|	14	670	3,03E-01	3,39E-01	1,23	0,15	WT	bacA mutant	PA14_29990	bifunctional NADH:ubiquinone oxidoreductase subunit C/D
YP_790556.1|gi|116050625|	5	285	3,58E-02	1,08E-01	1,21	0,65	WT	bacA mutant	PA14_30010	NADH dehydrogenase subunit B
YP_790560.1|gi|116050621|	24	1414	3,37E-03	4,75E-02	1,33	0,99	WT	bacA mutant	PA14_30050	isocitrate lyase
YP_790564.1|gi|116050617|	4	237	6,13E-01	4,80E-01	1,09	0,07	bacA mutant	WT	PA14_30100	hypothetical protein
YP_790565.1|gi|116050616|	12	559	7,03E-01	5,16E-01	1,11	0,06	WT	bacA mutant	PA14_30110	adenylosuccinate lyase
YP_790567.1|gi|116050614|	3	190	1,69E-01	2,35E-01	1,42	0,25	bacA mutant	WT	PA14_30140	hypothetical protein
YP_790568.1|gi|116050613|	7	365	1,10E-01	1,89E-01	1,09	0,35	bacA mutant	WT	PA14_30150	tRNA-specific 2-thiouridylase MnmA
YP_790569.1|gi|116050612|	2	83	7,73E-02	1,53E-01	1,24	0,44	WT	bacA mutant	PA14_30160	hypothetical protein

YP_790570.1|gi|116050611|	8	572	4,84E-02	1,20E-01	1,37	0,57	bacA mutant	WT	PA14_30180	monomeric isocitrate dehydrogenase
YP_790571.1|gi|116050610|	40	3378	4,87E-03	4,75E-02	1,26	0,98	WT	bacA mutant	PA14_30190	isocitrate dehydrogenase
YP_790574.1|gi|116050607|	8	279	5,34E-01	4,50E-01	1,10	0,08	WT	bacA mutant	PA14_30230	ATP-dependent Clp protease, ATP-binding subunit ClpA
YP_790577.1|gi|116050604|	2	87	9,58E-01	5,73E-01	1,03	0,05	WT	bacA mutant	PA14_30270	leucyl/phenylalanyl-tRNA--protein transferase
YP_790578.1|gi|116050603|	7	879	2,09E-01	2,70E-01	1,24	0,21	WT	bacA mutant	PA14_30280	thioredoxin reductase 1
YP_790580.1|gi|116050601|	5	177	7,10E-02	1,45E-01	1,53	0,46	WT	bacA mutant	PA14_30310	outer-membrane lipoprotein carrier protein
YP_790581.1|gi|116050600|	4	107	3,44E-01	3,59E-01	1,13	0,13	WT	bacA mutant	PA14_30320	recombination factor protein RarA
YP_790582.1|gi|116050599|	10	531	8,86E-01	5,56E-01	1,01	0,05	bacA mutant	WT	PA14_30330	seryl-tRNA synthetase
YP_790583.1|gi|116050598|	8	442	2,42E-03	4,16E-02	1,27	1,00	bacA mutant	WT	PA14_30340	siroheme synthase
YP_790591.1|gi|116050590|	4	200	1,51E-01	2,21E-01	1,19	0,28	bacA mutant	WT	PA14_30430	thiosulfate sulfurtransferase
YP_790602.1|gi|116050579|	2	66	3,37E-01	3,55E-01	1,32	0,14	bacA mutant	WT	PA14_30570	periplasmic spermidine/putrescine-binding protein
YP_790606.1|gi|116050575|	8	415	2,94E-02	9,98E-02	1,17	0,70	WT	bacA mutant	PA14_30620	AraC family transcriptional regulator
YP_790607.1|gi|116050574|	16	874	1,99E-01	2,60E-01	1,14	0,22	bacA mutant	WT	PA14_30630	FAD-dependent monooxygenase
YP_790613.1|gi|116050568|	3	193	1,84E-02	8,13E-02	1,87	0,82	bacA mutant	WT	PA14_30710	osmoprotectant transporter activator protein
YP_790616.1|gi|116050565|	2	61	1,08E-01	1,87E-01	1,40	0,35	WT	bacA mutant	PA14_30750	tryptophan oxygenase
YP_790620.1|gi|116050561|	4	146	2,63E-02	9,65E-02	1,40	0,73	WT	bacA mutant	PA14_30800	hypothetical protein
YP_790622.1|gi|116050559|	3	164	8,73E-01	5,55E-01	1,46	0,05	WT	bacA mutant	PA14_30820	methyl-accepting chemotaxis transducer
YP_790623.1|gi|116050558|	5	224	4,43E-01	4,09E-01	1,21	0,10	WT	bacA mutant	PA14_30830	two-component response regulator
YP_790624.1|gi|116050557|	6	324	6,47E-01	4,93E-01	1,05	0,07	bacA mutant	WT	PA14_30840	signal transduction histidine kinase
YP_790642.1|gi|116050539|	2	46	7,14E-01	5,18E-01	1,08	0,06	WT	bacA mutant	PA14_31040	cation efflux system protein
YP_790670.1|gi|116050511|	2	99	4,21E-01	3,98E-01	1,23	0,11	WT	bacA mutant	PA14_31360	hypothetical protein
YP_790671.1|gi|116050510|	3	207	5,87E-05	1,92E-02	1,48	1,00	WT	bacA mutant	PA14_31370	hypothetical protein
YP_790680.1|gi|116050501|	2	78	3,07E-01	3,41E-01	1,39	0,15	WT	bacA mutant	PA14_31470	AMP-binding protein
YP_790682.1|gi|116050499|	6	341	8,72E-01	5,55E-01	1,02	0,05	bacA mutant	WT	PA14_31500	AMP-binding protein
YP_790683.1|gi|116050498|	9	551	1,44E-02	7,30E-02	1,67	0,87	WT	bacA mutant	PA14_31510	short-chain dehydrogenase
YP_790684.1|gi|116050497|	14	1245	3,11E-01	3,43E-01	1,07	0,15	WT	bacA mutant	PA14_31530	acyl-CoA thiolase

YP_790685.1|gi|116050496|	8	424	8,26E-01	5,48E-01	1,02	0,05	WT	bacA mutant	PA14_31540	acyl-CoA dehydrogenase
YP_790686.1|gi|116050495|	5	245	2,60E-01	3,08E-01	1,15	0,18	WT	bacA mutant	PA14_31560	LysR family transcriptional regulator
YP_790692.1|gi|116050489|	7	298	3,70E-03	4,75E-02	1,22	0,99	WT	bacA mutant	PA14_31650	exonuclease III
YP_790693.1|gi|116050488|	4	233	2,48E-02	9,43E-02	1,55	0,75	WT	bacA mutant	PA14_31660	hypothetical protein
YP_790694.1|gi|116050487|	10	526	9,04E-01	5,59E-01	1,06	0,05	bacA mutant	WT	PA14_31680	hypothetical protein
YP_790705.1|gi|116050476|	9	1097	3,45E-02	1,06E-01	1,68	0,66	WT	bacA mutant	PA14_31810	thiol peroxidase
YP_790707.1|gi|116050474|	6	312	8,05E-04	2,86E-02	1,81	1,00	bacA mutant	WT	PA14_31840	hypothetical protein
YP_790708.1|gi|116050473|	7	456	2,69E-01	3,15E-01	1,13	0,17	bacA mutant	WT	PA14_31850	hypothetical protein
YP_790709.1|gi|116050472|	6	283	9,64E-01	5,74E-01	1,02	0,05	WT	bacA mutant	PA14_31870	RND efflux membrane fusion protein
YP_790712.1|gi|116050469|	5	225	6,14E-01	4,81E-01	1,26	0,07	bacA mutant	WT	PA14_31920	outer membrane protein
YP_790733.1|gi|116050448|	5	322	1,70E-01	2,35E-01	1,29	0,25	WT	bacA mutant	PA14_32280	hypothetical protein
YP_790745.1|gi|116050436|	3	152	1,49E-01	2,19E-01	1,22	0,28	bacA mutant	WT	PA14_32410	transcriptional regulator MexT
YP_790746.1|gi|116050435|	5	338	3,24E-01	3,50E-01	1,17	0,14	bacA mutant	WT	PA14_32420	oxidoreductase
YP_790761.1|gi|116050420|	3	133	5,47E-02	1,28E-01	1,51	0,53	WT	bacA mutant	PA14_32610	disulfide isomerase/thiol-disulfide oxidase
YP_790787.1|gi|116050395|	4	149	6,32E-01	4,87E-01	1,08	0,07	WT	bacA mutant	PA14_32950	hypothetical protein
YP_790789.1|gi|116054442|	3	152	1,75E-01	2,40E-01	1,36	0,25	bacA mutant	WT	PA14_32985	glycine cleavage system protein H
YP_790790.1|gi|116050393|	12	521	8,74E-01	5,55E-01	1,11	0,05	WT	bacA mutant	PA14_33000	glycine dehydrogenase
YP_790791.1|gi|116050392|	2	240	2,72E-01	3,16E-01	1,16	0,17	bacA mutant	WT	PA14_33010	serine hydroxymethyltransferase
YP_790793.1|gi|116050390|	12	633	1,32E-02	6,92E-02	1,18	0,88	bacA mutant	WT	PA14_33040	glycine cleavage system protein T2
YP_790814.1|gi|116050369|	6	397	6,28E-01	4,86E-01	1,07	0,07	WT	bacA mutant	PA14_33300	hypothetical protein
YP_790815.1|gi|116050368|	15	1139	6,30E-01	4,86E-01	1,02	0,07	bacA mutant	WT	PA14_33310	hypothetical protein
YP_790816.1|gi|116050367|	8	646	7,25E-01	5,20E-01	1,01	0,06	bacA mutant	WT	PA14_33320	hypothetical protein
YP_790817.1|gi|116050366|	6	243	1,43E-01	2,17E-01	1,23	0,29	bacA mutant	WT	PA14_33330	hypothetical protein
YP_790819.1|gi|116050364|	2	41	2,96E-02	1,00E-01	1,32	0,70	bacA mutant	WT	PA14_33350	hypothetical protein
YP_790827.1|gi|116050356|	2	91	2,21E-01	2,79E-01	1,23	0,20	bacA mutant	WT	PA14_33450	trehalase
YP_790830.1|gi|116050353|	5	228	2,86E-03	4,37E-02	2,63	1,00	bacA mutant	WT	PA14_33500	diaminobutyrate--2-oxoglutarate aminotransferase

YP_790833.1|gi|116050350|	6	309	1,04E-01	1,83E-01	1,40	0,36	WT	bacA mutant	PA14_33530	hypothetical protein
YP_790836.1|gi|116050347|	5	402	5,02E-02	1,22E-01	1,21	0,56	WT	bacA mutant	PA14_33560	adhesion protein
YP_790841.1|gi|116050342|	2	89	1,74E-01	2,39E-01	1,99	0,25	bacA mutant	WT	PA14_33610	peptide synthase
YP_790842.1|gi|116050341|	3	127	5,35E-01	4,50E-01	1,19	0,08	bacA mutant	WT	PA14_33630	protein PvdJ
YP_790844.1|gi|116050339|	19	1104	3,09E-01	3,42E-01	1,30	0,15	bacA mutant	WT	PA14_33680	ferripyoverdine receptor
YP_790846.1|gi|116050337|	3	177	3,45E-03	4,75E-02	2,45	0,99	bacA mutant	WT	PA14_33700	pyoverdine synthetase F
YP_790848.1|gi|116050335|	2	116	6,90E-03	5,79E-02	1,55	0,96	bacA mutant	WT	PA14_33720	protein PvdN
YP_790849.1|gi|116050334|	5	242	1,38E-01	2,13E-01	1,27	0,30	bacA mutant	WT	PA14_33730	dipeptidase
YP_790850.1|gi|116050333|	7	270	9,89E-03	6,57E-02	1,97	0,92	bacA mutant	WT	PA14_33740	protein PvdP
YP_790851.1|gi|116050332|	4	166	4,30E-01	4,04E-01	1,24	0,11	bacA mutant	WT	PA14_33750	outer membrane protein
YP_790856.1|gi|116050327|	10	622	2,06E-03	3,96E-02	1,97	1,00	bacA mutant	WT	PA14_33810	L-ornithine N5-oxygenase
YP_790860.1|gi|116050323|	2	92	6,63E-02	1,41E-01	1,57	0,48	bacA mutant	WT	PA14_33860	L-lactate dehydrogenase
YP_790898.1|gi|116050285|	6	394	7,91E-01	5,39E-01	1,02	0,06	bacA mutant	WT	PA14_34330	hypothetical protein
YP_790908.1|gi|116050275|	5	337	4,01E-01	3,87E-01	1,09	0,11	bacA mutant	WT	PA14_34460	hypothetical protein
YP_790909.1|gi|116050274|	12	858	2,20E-02	9,21E-02	1,63	0,78	bacA mutant	WT	PA14_34490	hypothetical protein
YP_790916.1|gi|116050267|	14	895	1,78E-01	2,43E-01	1,11	0,24	bacA mutant	WT	PA14_34600	glyceraldehyde-3-phosphate dehydrogenase
YP_790922.1|gi|116050261|	2	128	5,63E-01	4,59E-01	1,05	0,08	bacA mutant	WT	PA14_34680	oxidoreductase
YP_790934.1|gi|116050249|	4	157	5,57E-01	4,57E-01	1,48	0,08	WT	bacA mutant	PA14_34810	non-ribosomal peptide synthetase
YP_790935.1|gi|116050248|	8	451	2,05E-01	2,66E-01	1,07	0,22	bacA mutant	WT	PA14_34820	regulatory protein
YP_790936.1|gi|116050247|	6	454	4,83E-03	4,75E-02	1,17	0,98	bacA mutant	WT	PA14_34830	regulatory protein
YP_790937.1|gi|116050246|	32	1950	4,83E-01	4,28E-01	1,21	0,09	WT	bacA mutant	PA14_34840	non-ribosomal peptide synthetase
YP_790938.1|gi|116050245|	2	41	4,23E-02	1,16E-01	1,55	0,60	WT	bacA mutant	PA14_34850	tRNA synthase
YP_790939.1|gi|116050244|	14	1458	5,69E-01	4,60E-01	1,12	0,08	WT	bacA mutant	PA14_34870	chitinase
YP_790947.1|gi|116050236|	3	109	8,02E-01	5,42E-01	1,11	0,06	bacA mutant	WT	PA14_34990	TonB-dependent receptor
YP_790963.1|gi|116050220|	2	67	3,57E-01	3,66E-01	1,19	0,13	WT	bacA mutant	PA14_35170	redox-sensing activator of soxS
YP_790968.1|gi|116050215|	5	222	8,59E-01	5,53E-01	1,02	0,05	WT	bacA mutant	PA14_35240	hypothetical protein

YP_790977.1|gi|116050206|	3	71	7,12E-01	5,18E-01	1,08	0,06	WT	bacA mutant	PA14_35370	transcriptional regulator PtxS
YP_790986.1|gi|116050197|	30	2570	1,24E-03	3,32E-02	1,55	1,00	WT	bacA mutant	PA14_35490	dihydrolipoamide dehydrogenase
YP_790987.1|gi|116050196|	26	2098	4,97E-03	4,78E-02	1,38	0,98	WT	bacA mutant	PA14_35500	branched-chain alpha-keto acid dehydrogenase subunit E2
YP_790988.1|gi|116050195|	6	329	5,91E-03	5,30E-02	2,55	0,97	WT	bacA mutant	PA14_35520	2-oxoisovalerate dehydrogenase subunit beta
YP_790989.1|gi|116050194|	15	974	3,63E-02	1,08E-01	1,45	0,65	WT	bacA mutant	PA14_35530	2-oxoisovalerate dehydrogenase subunit alpha
YP_791004.1|gi|116050179|	2	96	5,38E-01	4,51E-01	1,07	0,08	WT	bacA mutant	PA14_35720	hypothetical protein
YP_791008.1|gi|116050175|	2	50	1,33E-01	2,09E-01	1,49	0,30	WT	bacA mutant	PA14_35760	hypothetical protein
YP_791011.1|gi|116050172|	6	293	8,95E-01	5,58E-01	1,01	0,05	bacA mutant	WT	PA14_35790	homospermidine synthase
YP_791012.1|gi|116050171|	4	80	3,24E-01	3,50E-01	1,28	0,14	WT	bacA mutant	PA14_35800	hypothetical protein
YP_791013.1|gi|116050170|	2	84	9,86E-02	1,78E-01	1,48	0,38	WT	bacA mutant	PA14_35810	hypothetical protein
YP_791015.1|gi|116050168|	3	220	3,95E-01	3,83E-01	1,18	0,12	WT	bacA mutant	PA14_35830	cointegrate resolution protein T
YP_791016.1|gi|116050167|	2	168	7,84E-01	5,39E-01	1,02	0,06	bacA mutant	WT	PA14_35840	hypothetical protein
YP_791017.1|gi|116050166|	2	86	3,28E-01	3,52E-01	1,23	0,14	bacA mutant	WT	PA14_35850	hypothetical protein
YP_791020.1|gi|116050163|	2	59	8,22E-01	5,48E-01	1,02	0,05	bacA mutant	WT	PA14_35890	diaminobutyrate--2-oxoglutarate aminotransferase
YP_791046.1|gi|116050137|	2	93	1,45E-01	2,18E-01	1,35	0,29	bacA mutant	WT	PA14_36200	ABC transporter substrate-binding protein
YP_791049.1|gi|116050134|	2	57	7,80E-01	5,38E-01	1,01	0,06	bacA mutant	WT	PA14_36250	hypothetical protein
YP_791053.1|gi|116050130|	4	169	4,56E-01	4,16E-01	1,13	0,10	bacA mutant	WT	PA14_36290	hypothetical protein
YP_791055.1|gi|116050128|	10	707	3,91E-01	3,81E-01	1,14	0,12	WT	bacA mutant	PA14_36310	hydrogen cyanide synthase HcnC
YP_791056.1|gi|116050127|	15	791	5,00E-02	1,21E-01	1,21	0,56	WT	bacA mutant	PA14_36320	hydrogen cyanide synthase HcnB
YP_791067.1|gi|116050118|	4	302	9,54E-01	5,72E-01	1,01	0,05	bacA mutant	WT	PA14_36450	hypothetical protein
YP_791072.1|gi|116050113|	4	211	5,43E-01	4,52E-01	1,06	0,08	bacA mutant	WT	PA14_36500	hypothetical protein
YP_791074.1|gi|116050111|	5	409	7,00E-01	5,14E-01	1,06	0,06	bacA mutant	WT	PA14_36530	hypothetical protein
YP_791079.1|gi|116050106|	2	39	1,33E-01	2,09E-01	1,38	0,30	bacA mutant	WT	PA14_36580	glycosyl hydrolase
YP_791083.1|gi|116050103|	6	284	2,41E-01	2,95E-01	1,17	0,19	bacA mutant	WT	PA14_36630	glycosyl hydrolase
YP_791084.1|gi|116050102|	2	70	2,32E-02	9,26E-02	6,69	0,76	bacA mutant	WT	PA14_36650	hypothetical protein
YP_791090.1|gi|116050096|	2	57	7,23E-01	5,19E-01	1,04	0,06	bacA mutant	WT	PA14_36710	glycogen branching protein

YP_791097.1|gi|116050089|	17	826	7,70E-03	5,89E-02	2,30	0,95	bacA mutant	WT	PA14_36810	hydroperoxidase II
YP_791100.1|gi|116050086|	2	79	4,64E-01	4,19E-01	1,28	0,10	bacA mutant	WT	PA14_36840	glycogen phosphorylase
YP_791103.1|gi|116050083|	2	76	6,64E-02	1,41E-01	1,86	0,48	bacA mutant	WT	PA14_36870	short-chain dehydrogenase
YP_791129.1|gi|116050057|	2	40	1,45E-01	2,18E-01	1,34	0,29	WT	bacA mutant	PA14_37200	hypothetical protein
YP_791130.1|gi|116050056|	3	134	1,67E-01	2,33E-01	1,36	0,26	bacA mutant	WT	PA14_37210	hypothetical protein
YP_791133.1|gi|116050053|	8	397	1,58E-01	2,26E-01	1,32	0,27	WT	bacA mutant	PA14_37260	porin
YP_791141.1|gi|116050045|	2	66	1,46E-04	2,46E-02	4,42	1,00	WT	bacA mutant	PA14_37370	esterase
YP_791159.1|gi|116050027|	6	232	6,18E-01	4,82E-01	1,07	0,07	bacA mutant	WT	PA14_37610	kynureninase
YP_791167.1|gi|116050019|	7	335	1,37E-01	2,11E-01	1,22	0,30	bacA mutant	WT	PA14_37710	elongation factor G
YP_791169.1|gi|116054447|	12	773	7,73E-01	5,36E-01	1,02	0,06	WT	bacA mutant	PA14_37745	carbamoyl transferase
YP_791172.1|gi|116050015|	3	130	1,22E-02	6,84E-02	1,24	0,89	WT	bacA mutant	PA14_37780	hypothetical protein
YP_791197.1|gi|116049992|	3	195	4,61E-01	4,17E-01	1,22	0,10	bacA mutant	WT	PA14_38110	serine/threonine transporter SstT
YP_791199.1|gi|116049990|	2	152	3,80E-01	3,76E-01	1,40	0,12	WT	bacA mutant	PA14_38140	glutamine synthetase
YP_791206.1|gi|116049983|	5	236	5,66E-01	4,60E-01	1,04	0,08	bacA mutant	WT	PA14_38220	hypothetical protein
YP_791214.1|gi|116049975|	12	824	2,40E-02	9,35E-02	1,40	0,75	WT	bacA mutant	PA14_38330	glutathione reductase
YP_791216.1|gi|116049973|	4	292	2,02E-03	3,96E-02	1,32	1,00	WT	bacA mutant	PA14_38350	UTP-glucose-1-phosphate uridylyltransferase
YP_791224.1|gi|116049966|	8	433	4,18E-01	3,97E-01	1,05	0,11	bacA mutant	WT	PA14_38440	citronelloyl-CoA dehydrogenase, GnyD
YP_791225.1|gi|116049965|	17	1054	8,74E-01	5,55E-01	1,02	0,05	bacA mutant	WT	PA14_38460	acyl-CoA carboxyltransferase subunit beta
YP_791226.1|gi|116049964|	2	79	4,38E-02	1,17E-01	1,37	0,59	bacA mutant	WT	PA14_38470	gamma-carboxygeranoyl-CoA hydratase
YP_791227.1|gi|116049963|	7	363	3,28E-01	3,52E-01	1,11	0,14	bacA mutant	WT	PA14_38480	alpha subunit of geranoyl-CoA carboxylase, GnyA
YP_791228.1|gi|116049962|	3	143	1,25E-02	6,84E-02	1,14	0,89	WT	bacA mutant	PA14_38490	hydroxymethylglutaryl-CoA lyase
YP_791230.1|gi|116049960|	19	1469	1,51E-02	7,49E-02	1,26	0,86	bacA mutant	WT	PA14_38510	homogentisate 1,2-dioxygenase
YP_791231.1|gi|116049959|	13	977	5,86E-03	5,29E-02	1,37	0,97	bacA mutant	WT	PA14_38530	fumarylacetoacetase
YP_791232.1|gi|116049958|	7	307	5,06E-02	1,22E-01	1,48	0,55	bacA mutant	WT	PA14_38550	maleylacetoacetate isomerase
YP_791234.1|gi|116049956|	4	121	7,37E-02	1,49E-01	1,19	0,45	WT	bacA mutant	PA14_38570	transcriptional regulator
YP_791236.1|gi|116049954|	5	297	3,86E-01	3,80E-01	1,05	0,12	WT	bacA mutant	PA14_38590	3-hydroxybutyrate dehydrogenase

YP_791238.1|gi|116049952|	18	1294	2,42E-01	2,95E-01	1,15	0,19	bacA mutant	WT	PA14_38630	acetyl-CoA acetyltransferase
YP_791239.1|gi|116049951|	4	235	6,91E-01	5,10E-01	1,02	0,06	bacA mutant	WT	PA14_38640	CoA transferase subunit B
YP_791240.1|gi|116049950|	4	249	1,14E-02	6,67E-02	1,61	0,90	bacA mutant	WT	PA14_38660	CoA transferase, subunit A
YP_791271.1|gi|116049920|	3	163	2,45E-01	2,98E-01	1,26	0,18	WT	bacA mutant	PA14_39070	hypothetical protein
YP_791289.1|gi|116049902|	2	161	5,07E-01	4,37E-01	1,20	0,09	WT	bacA mutant	PA14_39270	hypothetical protein
YP_791290.1|gi|116049901|	4	212	9,87E-01	5,82E-01	1,00	0,05	WT	bacA mutant	PA14_39280	ribokinase
YP_791291.1|gi|116049900|	3	105	2,60E-02	9,65E-02	1,11	0,73	bacA mutant	WT	PA14_39300	ribose operon repressor RbsR
YP_791294.1|gi|116049897|	3	162	4,82E-03	4,75E-02	2,41	0,98	WT	bacA mutant	PA14_39350	ribose ABC transporter substrate-binding protein
YP_791296.1|gi|116049895|;<br>YP_791900.1|gi|116049297|	7	412	7,92E-01	5,39E-01	1,03	0,06	bacA mutant	WT	PA14_39390	30S ribosomal protein S6 modification protein
YP_791297.1|gi|116049894|	2	123	1,85E-02	8,13E-02	2,00	0,81	WT	bacA mutant	PA14_39410	hypothetical protein
YP_791307.1|gi|116049884|	2	89	6,87E-01	5,08E-01	1,13	0,06	WT	bacA mutant	PA14_39560	chemotaxis transducer
YP_791325.1|gi|116049866|	3	145	2,50E-02	9,43E-02	1,62	0,74	bacA mutant	WT	PA14_39780	hypothetical protein
YP_791339.1|gi|116049854|	3	186	1,18E-01	1,95E-01	1,42	0,33	WT	bacA mutant	PA14_39960	phenazine biosynthesis protein
YP_791345.1|gi|116049848|	2	163	1,45E-01	2,18E-01	1,82	0,28	WT	bacA mutant	PA14_40030	hypothetical protein
YP_791349.1|gi|116049844|	2	147	3,44E-01	3,59E-01	1,34	0,13	bacA mutant	WT	PA14_40070	glutathione S-transferase
YP_791350.1|gi|116049843|	2	69	7,57E-01	5,29E-01	1,07	0,06	WT	bacA mutant	PA14_40080	hypothetical protein
YP_791359.1|gi|116049834|	5	224	2,88E-01	3,29E-01	1,10	0,16	WT	bacA mutant	PA14_40200	oxidoreductase
YP_791367.1|gi|116049826|	5	259	6,00E-02	1,35E-01	1,40	0,51	bacA mutant	WT	PA14_40280	hypothetical protein
YP_791368.1|gi|116049825|	3	258	1,75E-02	8,01E-02	1,60	0,83	WT	bacA mutant	PA14_40290	LasA protease
YP_791377.1|gi|116049816|	2	44	7,91E-03	5,95E-02	3,78	0,95	WT	bacA mutant	PA14_40390	molybdate-binding periplasmic protein precursor modA
YP_791380.1|gi|116049813|	6	247	2,16E-02	9,13E-02	1,66	0,78	WT	bacA mutant	PA14_40430	hypothetical protein
YP_791394.1|gi|116049799|	4	161	8,30E-03	6,00E-02	2,33	0,94	WT	bacA mutant	PA14_40630	hypothetical protein
YP_791405.1|gi|116049788|	7	353	5,67E-02	1,32E-01	1,24	0,52	bacA mutant	WT	PA14_40770	sulfite reductase
YP_791406.1|gi|116049787|	3	152	6,69E-01	5,02E-01	1,05	0,07	WT	bacA mutant	PA14_40780	hypothetical protein
YP_791410.1|gi|116049783|	14	952	3,44E-02	1,06E-01	1,23	0,66	WT	bacA mutant	PA14_40830	oxidoreductase

YP_791411.1|gi|116049782|	4	192	8,49E-01	5,52E-01	1,02	0,05	WT	bacA mutant	PA14_40840	periplasmic protease
YP_791412.1|gi|116049781|	3	151	5,56E-01	4,57E-01	1,04	0,08	bacA mutant	WT	PA14_40850	hypothetical protein
YP_791414.1|gi|116049779|	2	84	8,17E-01	5,46E-01	1,06	0,05	WT	bacA mutant	PA14_40880	hypothetical protein
YP_791415.1|gi|116049778|	4	302	2,33E-01	2,88E-01	1,20	0,19	WT	bacA mutant	PA14_40890	short chain dehydrogenase
YP_791418.1|gi|116049775|	2	58	9,26E-01	5,65E-01	1,88	0,05	WT	bacA mutant	PA14_40930	hypothetical protein
YP_791421.1|gi|116049772|	4	177	4,44E-03	4,75E-02	1,28	0,99	bacA mutant	WT	PA14_40960	pilin biosynthetic protein
YP_791422.1|gi|116049771|	7	350	6,17E-02	1,36E-01	1,18	0,50	WT	bacA mutant	PA14_40980	enoyl-CoA hydratase
YP_791425.1|gi|116049768|	5	240	4,73E-01	4,23E-01	1,25	0,10	bacA mutant	WT	PA14_41020	Orn/Arg/Lys decarboxylase
YP_791433.1|gi|116049760|	4	227	1,98E-01	2,60E-01	1,16	0,22	bacA mutant	WT	PA14_41130	ABC transporter substrate-binding protein NppA2
YP_791436.1|gi|116049757|;<br>YP_791177.1|gi|116050010|	5	178	1,46E-01	2,18E-01	1,33	0,28	bacA mutant	WT	PA14_41160	peptidyl nucleoside antibiotic ABC transporter ATP-binding protein NppD
YP_791437.1|gi|116049756|	2	92	2,63E-02	9,65E-02	1,56	0,73	WT	bacA mutant	PA14_41170	NADH-dependent enoyl-ACP reductase
YP_791438.1|gi|116049755|	14	890	8,01E-01	5,42E-01	1,02	0,06	bacA mutant	WT	PA14_41190	peptidyl-prolyl cis-trans isomerase D
YP_791439.1|gi|116049754|	3	359	1,98E-01	2,60E-01	1,38	0,22	bacA mutant	WT	PA14_41210	DNA-binding protein HU
YP_791440.1|gi|116049753|	21	1118	2,16E-01	2,76E-01	1,20	0,21	bacA mutant	WT	PA14_41220	Lon protease
YP_791441.1|gi|116049752|	14	770	8,87E-03	6,14E-02	1,14	0,94	bacA mutant	WT	PA14_41230	ATP-dependent protease ATP-binding subunit ClpX
YP_791442.1|gi|116049751|	6	275	1,47E-02	7,44E-02	1,32	0,86	WT	bacA mutant	PA14_41240	ATP-dependent Clp protease proteolytic subunit
YP_791443.1|gi|116049750|	16	938	8,34E-01	5,50E-01	1,02	0,05	bacA mutant	WT	PA14_41250	trigger factor
YP_791450.1|gi|116049743|	12	748	1,49E-01	2,19E-01	1,14	0,28	WT	bacA mutant	PA14_41360	cysteinyl-tRNA synthetase
YP_791451.1|gi|116049742|	18	1226	7,73E-01	5,36E-01	1,02	0,06	bacA mutant	WT	PA14_41380	glutaminyl-tRNA synthetase
YP_791452.1|gi|116049741|	7	372	4,36E-02	1,17E-01	1,79	0,59	WT	bacA mutant	PA14_41390	peptidyl-prolyl cis-trans isomerase B
YP_791453.1|gi|116049740|	2	77	7,24E-01	5,20E-01	1,04	0,06	bacA mutant	WT	PA14_41400	UDP-2,3-diacylglucosamine hydrolase
YP_791455.1|gi|116049738|	2	65	3,14E-01	3,44E-01	1,02	0,15	WT	bacA mutant	PA14_41430	hypothetical protein
YP_791456.1|gi|116049737|	15	1092	4,55E-01	4,16E-01	1,10	0,10	WT	bacA mutant	PA14_41440	hypothetical protein
YP_791458.1|gi|116049735|	34	2312	3,89E-01	3,81E-01	1,22	0,12	WT	bacA mutant	PA14_41470	bifunctional aconitate hydratase 2/2-methylisocitrate dehydratase
YP_791468.1|gi|116049726|	28	2681	9,24E-01	5,65E-01	1,07	0,05	bacA mutant	WT	PA14_41570	major porin and structural outer membrane porin OprF precursor

YP_791469.1|gi|116054443|	2	82	1,36E-01	2,11E-01	1,28	0,30	bacA mutant	WT	PA14_41575	RNA polymerase sigma factor SigX
YP_791470.1|gi|116049725|	8	585	8,46E-01	5,52E-01	1,15	0,05	WT	bacA mutant	PA14_41590	cytoplasmic membrane protein
YP_791472.1|gi|116049723|	2	87	5,95E-01	4,71E-01	1,44	0,07	WT	bacA mutant	PA14_41630	cytoplasmic membrane-associated protein
YP_791473.1|gi|116049722|	5	265	7,80E-04	2,86E-02	2,03	1,00	WT	bacA mutant	PA14_41640	ribonuclease activity regulator protein RraA
YP_791475.1|gi|116049720|	35	2427	4,48E-02	1,18E-01	1,30	0,59	bacA mutant	WT	PA14_41670	phosphoenolpyruvate synthase
YP_791476.1|gi|116049719|	5	181	4,60E-03	4,75E-02	1,61	0,99	WT	bacA mutant	PA14_41680	hypothetical protein
YP_791477.1|gi|116049718|	2	83	9,71E-01	5,77E-01	1,00	0,05	WT	bacA mutant	PA14_41690	hypothetical protein
YP_791478.1|gi|116049717|	3	171	9,01E-01	5,59E-01	1,14	0,05	WT	bacA mutant	PA14_41710	hypothetical protein
YP_791479.1|gi|116049716|	3	142	1,14E-01	1,92E-01	1,24	0,34	WT	bacA mutant	PA14_41730	hypothetical protein
YP_791488.1|gi|116049707|	5	176	2,83E-02	9,88E-02	1,42	0,71	WT	bacA mutant	PA14_41830	phosphoserine phosphatase
YP_791491.1|gi|116049704|	8	476	1,92E-01	2,54E-01	1,33	0,23	WT	bacA mutant	PA14_41870	transcriptional regulator CysB
YP_791492.1|gi|116049703|	2	48	2,46E-01	2,98E-01	1,33	0,18	WT	bacA mutant	PA14_41880	universal stress protein
YP_791493.1|gi|116049702|	5	225	1,62E-01	2,30E-01	1,16	0,26	bacA mutant	WT	PA14_41900	2-dehydropantoate 2-reductase
YP_791495.1|gi|116049700|	3	172	1,31E-01	2,07E-01	1,14	0,31	WT	bacA mutant	PA14_41920	phospho-2-dehydro-3-deoxyheptonate aldolase
YP_791497.1|gi|116049698|	7	836	3,88E-03	4,75E-02	1,93	0,99	WT	bacA mutant	PA14_41950	enoyl-CoA hydratase
YP_791499.1|gi|116049696|	6	326	2,52E-01	3,03E-01	1,32	0,18	bacA mutant	WT	PA14_41970	hypothetical protein
YP_791500.1|gi|116049695|	6	262	2,29E-02	9,26E-02	1,36	0,77	WT	bacA mutant	PA14_41980	hypothetical protein
YP_791503.1|gi|116049692|	7	358	1,77E-02	8,05E-02	1,39	0,82	bacA mutant	WT	PA14_42010	amidotransferase
YP_791504.1|gi|116049691|	2	85	7,92E-01	5,39E-01	1,04	0,06	WT	bacA mutant	PA14_42020	hypothetical protein
YP_791508.1|gi|116049687|	13	550	7,10E-03	5,85E-02	1,58	0,96	WT	bacA mutant	PA14_42080	3-hydroxyacyl-CoA dehydrogenase
YP_791509.1|gi|116049686|	3	218	1,20E-03	3,32E-02	2,30	1,00	WT	bacA mutant	PA14_42090	acetyl-CoA acetyltransferase
YP_791515.1|gi|116049680|	2	95	1,46E-01	2,18E-01	1,19	0,28	bacA mutant	WT	PA14_42160	hypothetical protein
YP_791516.1|gi|116049679|	2	72	5,73E-01	4,62E-01	1,05	0,08	bacA mutant	WT	PA14_42180	hypothetical protein
YP_791556.1|gi|116049639|	4	216	4,80E-01	4,27E-01	1,11	0,09	bacA mutant	WT	PA14_42670	hypothetical protein
YP_791558.1|gi|116049637|	5	246	2,56E-02	9,61E-02	1,42	0,74	WT	bacA mutant	PA14_42690	spermidine synthase
YP_791561.1|gi|116049634|	5	284	8,24E-01	5,48E-01	1,02	0,05	WT	bacA mutant	PA14_42720	enolase-phosphatase

YP_791562.1|gi|116049633|	3	365	2,45E-01	2,98E-01	1,23	0,18	bacA mutant	WT	PA14_42730	oxidase
YP_791565.1|gi|116049630|	4	183	1,15E-01	1,93E-01	1,24	0,34	WT	bacA mutant	PA14_42760	chorismate synthase
YP_791568.1|gi|116049627|	3	193	7,44E-01	5,27E-01	1,14	0,06	WT	bacA mutant	PA14_42790	N5-glutamine S-adenosyl-L-methionine-dependent methyltransferase
YP_791569.1|gi|116049626|	7	514	1,41E-02	7,23E-02	1,46	0,87	WT	bacA mutant	PA14_42820	isochorismatase family hydrolase
YP_791572.1|gi|116049623|	4	153	9,89E-04	3,01E-02	1,57	1,00	WT	bacA mutant	PA14_42850	GTP cyclohydrolase I
YP_791573.1|gi|116049622|	2	121	2,28E-01	2,85E-01	1,17	0,20	bacA mutant	WT	PA14_42860	hypothetical protein
YP_791584.1|gi|116049611|	9	510	1,43E-01	2,17E-01	1,52	0,29	bacA mutant	WT	PA14_42980	ClpV2
YP_791588.1|gi|116049607|	21	1332	4,84E-01	4,28E-01	1,17	0,09	WT	bacA mutant	PA14_43030	HsiC2
YP_791589.1|gi|116049606|	10	659	3,61E-01	3,67E-01	1,29	0,13	WT	bacA mutant	PA14_43040	HsiB2
YP_791602.1|gi|116049593|	2	58	8,36E-01	5,50E-01	1,01	0,05	bacA mutant	WT	PA14_43190	oxidoreductase
YP_791607.1|gi|116049588|	2	101	1,61E-02	7,70E-02	2,48	0,84	WT	bacA mutant	PA14_43250	hypothetical protein
YP_791608.1|gi|116049587|	5	393	5,65E-01	4,60E-01	1,07	0,08	WT	bacA mutant	PA14_43270	tRNA 2-selenouridine synthase
YP_791609.1|gi|116049586|	7	427	9,29E-01	5,67E-01	1,01	0,05	bacA mutant	WT	PA14_43280	selenophosphate synthetase
YP_791610.1|gi|116049585|	3	133	3,57E-01	3,66E-01	1,70	0,13	bacA mutant	WT	PA14_43290	lipoprotein
YP_791628.1|gi|116049568|	4	176	5,87E-01	4,68E-01	1,03	0,07	WT	bacA mutant	PA14_43530	hypothetical protein
YP_791634.1|gi|116049562|	2	71	3,79E-02	1,11E-01	1,79	0,63	WT	bacA mutant	PA14_43610	AMP-binding protein
YP_791637.1|gi|116049559|	6	351	1,48E-01	2,19E-01	1,27	0,28	bacA mutant	WT	PA14_43640	NAD(P)H-dependent glycerol-3-phosphate dehydrogenase
YP_791641.1|gi|116049555|	2	127	5,98E-01	4,72E-01	1,09	0,07	WT	bacA mutant	PA14_43680	3-hydroxydecanoyl-ACP dehydratase
YP_791642.1|gi|116049554|	8	656	3,37E-02	1,06E-01	1,37	0,67	WT	bacA mutant	PA14_43690	3-oxoacyl-ACP synthase
YP_791654.1|gi|116049542|	4	225	1,82E-02	8,12E-02	1,69	0,82	WT	bacA mutant	PA14_43840	hypothetical protein
YP_791655.1|gi|116049541|	32	2347	9,54E-01	5,72E-01	1,01	0,05	WT	bacA mutant	PA14_43850	heat shock protein 90
YP_791659.1|gi|116049537|	2	80	4,27E-02	1,16E-01	1,47	0,60	WT	bacA mutant	PA14_43900	hypothetical protein
YP_791662.1|gi|116049534|	17	1099	1,12E-02	6,67E-02	1,55	0,91	WT	bacA mutant	PA14_43940	succinyl-CoA synthetase subunit alpha
YP_791663.1|gi|116049533|	19	1543	4,28E-02	1,16E-01	1,29	0,60	WT	bacA mutant	PA14_43950	succinyl-CoA synthetase subunit beta
YP_791664.1|gi|116049532|	24	2152	2,23E-02	9,26E-02	1,26	0,77	WT	bacA mutant	PA14_43970	dihydrolipoamide dehydrogenase
YP_791665.1|gi|116049531|	8	631	5,82E-01	4,66E-01	1,07	0,08	WT	bacA mutant	PA14_44000	dihydrolipoamide succinyltransferase

YP_791666.1|gi|116049530|	19	888	4,98E-02	1,21E-01	1,71	0,56	WT	bacA mutant	PA14_44010	2-oxoglutarate dehydrogenase E1
YP_791667.1|gi|116049529|	14	852	2,93E-02	9,98E-02	1,32	0,70	WT	bacA mutant	PA14_44020	succinate dehydrogenase iron-sulfur subunit
YP_791668.1|gi|116049528|	16	755	8,02E-02	1,56E-01	1,20	0,43	WT	bacA mutant	PA14_44030	succinate dehydrogenase flavoprotein subunit
YP_791669.1|gi|116049527|	2	95	5,48E-01	4,54E-01	1,14	0,08	bacA mutant	WT	PA14_44050	succinate dehydrogenase (D subunit)
YP_791670.1|gi|116049526|	4	222	6,60E-01	4,98E-01	1,03	0,07	WT	bacA mutant	PA14_44060	succinate dehydrogenase, cytochrome b556 subunit
YP_791671.1|gi|116049525|	23	1486	3,46E-02	1,06E-01	1,53	0,66	WT	bacA mutant	PA14_44070	type II citrate synthase
YP_791672.1|gi|116049524|	3	227	4,49E-02	1,18E-01	1,94	0,59	WT	bacA mutant	PA14_44080	hypothetical protein
YP_791676.1|gi|116049520|	7	311	2,95E-02	1,00E-01	1,18	0,70	WT	bacA mutant	PA14_44120	3-hydroxyisobutyrate dehydrogenase
YP_791680.1|gi|116049516|	3	145	1,98E-03	3,96E-02	1,40	1,00	WT	bacA mutant	PA14_44160	ATP-NAD kinase
YP_791690.1|gi|116049506|	4	171	2,01E-01	2,62E-01	1,13	0,22	WT	bacA mutant	PA14_44280	RNA 2'-O-ribose methyltransferase
YP_791691.1|gi|116049505|	30	1692	4,00E-02	1,14E-01	1,72	0,62	WT	bacA mutant	PA14_44290	aconitate hydratase
YP_791692.1|gi|116049504|	7	447	3,67E-01	3,71E-01	1,37	0,12	bacA mutant	WT	PA14_44300	aerotaxis receptor Aer
YP_791695.1|gi|116049502|	2	94	9,79E-01	5,79E-01	1,27	0,05	WT	bacA mutant	PA14_44340	cbb3-type cytochrome c oxidase subunit I
YP_791696.1|gi|116049501|	12	698	3,91E-01	3,81E-01	1,17	0,12	WT	bacA mutant	PA14_44350	cbb3-type cytochrome c oxidase subunit II
YP_791697.1|gi|116049500|	10	618	8,65E-01	5,53E-01	1,12	0,05	WT	bacA mutant	PA14_44360	cytochrome c oxidase, cbb3-type subunit III
YP_791699.1|gi|116049498|	6	467	9,49E-01	5,72E-01	1,02	0,05	bacA mutant	WT	PA14_44380	cbb3-type cytochrome c oxidase subunit II
YP_791701.1|gi|116049496|	11	708	5,28E-01	4,47E-01	1,19	0,08	bacA mutant	WT	PA14_44400	cytochrome c oxidase, cbb3-type subunit III
YP_791702.1|gi|116049495|	6	286	7,22E-01	5,19E-01	1,61	0,06	WT	bacA mutant	PA14_44420	ferredoxin
YP_791703.1|gi|116049494|	5	409	8,53E-01	5,53E-01	1,04	0,05	WT	bacA mutant	PA14_44430	hypothetical protein
YP_791707.1|gi|116049490|	15	724	8,65E-01	5,53E-01	1,05	0,05	WT	bacA mutant	PA14_44470	coproporphyrinogen III oxidase
YP_791709.1|gi|116049488|	4	224	3,34E-01	3,54E-01	1,20	0,14	WT	bacA mutant	PA14_44490	transcriptional regulator Anr
YP_791710.1|gi|116049487|	3	98	3,98E-03	4,75E-02	1,32	0,99	WT	bacA mutant	PA14_44500	adenine phosphoribosyltransferase
YP_791721.1|gi|116049476|	2	98	3,28E-01	3,52E-01	1,14	0,14	WT	bacA mutant	PA14_44630	DNA polymerase III subunits gamma and tau
YP_791724.1|gi|116049473|	7	316	2,41E-01	2,95E-01	1,39	0,19	bacA mutant	WT	PA14_44660	NAD-dependent DNA ligase LigA
YP_791725.1|gi|116049472|	8	955	7,16E-01	5,18E-01	1,04	0,06	bacA mutant	WT	PA14_44670	cell division protein ZipA
YP_791727.1|gi|116049470|	2	58	8,60E-01	5,53E-01	1,14	0,05	WT	bacA mutant	PA14_44690	GntR family transcriptional regulator

YP_791729.1|gi|116049468|	5	209	2,79E-01	3,22E-01	1,20	0,16	WT	bacA mutant	PA14_44710	xanthine dehydrogenase
YP_791732.1|gi|116049465|	5	249	8,40E-01	5,51E-01	1,01	0,05	WT	bacA mutant	PA14_44770	guanine deaminase
YP_791733.1|gi|116049464|	5	381	1,91E-01	2,54E-01	1,17	0,23	bacA mutant	WT	PA14_44780	transcriptional regulator
YP_791749.1|gi|116049448|	3	152	5,21E-02	1,25E-01	1,29	0,55	WT	bacA mutant	PA14_44980	TetR family transcriptional regulator
YP_791759.1|gi|116049438|	4	230	2,24E-03	4,08E-02	1,46	1,00	WT	bacA mutant	PA14_45100	hypothetical protein
YP_791760.1|gi|116049437|	8	395	2,07E-02	8,87E-02	1,82	0,79	WT	bacA mutant	PA14_45110	sulfate-binding protein of ABC transporter
YP_791769.1|gi|116049428|	2	56	4,62E-02	1,20E-01	1,22	0,58	WT	bacA mutant	PA14_45250	transcriptional regulator
YP_791771.1|gi|116049426|	9	569	3,90E-01	3,81E-01	1,45	0,12	WT	bacA mutant	PA14_45280	cytochrome c-type biogenesis protein
YP_791772.1|gi|116049425|	2	100	7,92E-01	5,39E-01	1,28	0,06	WT	bacA mutant	PA14_45290	cytochrome C-type biogenesis protein CcmH
YP_791773.1|gi|116049424|	5	256	7,86E-01	5,39E-01	1,03	0,06	bacA mutant	WT	PA14_45300	cytochrome C biogenesis protein CcmG
YP_791775.1|gi|116049422|	3	136	2,20E-01	2,78E-01	1,40	0,20	WT	bacA mutant	PA14_45330	cytochrome c-type biogenesis protein CcmE
YP_791788.1|gi|116049409|	6	444	3,76E-02	1,11E-01	1,27	0,64	WT	bacA mutant	PA14_45500	purine-binding chemotaxis protein
YP_791793.1|gi|116049404|	11	622	9,76E-01	5,78E-01	1,01	0,05	bacA mutant	WT	PA14_45580	chemotaxis-specific methylesterase
YP_791794.1|gi|116049403|	3	125	5,94E-01	4,71E-01	1,21	0,07	WT	bacA mutant	PA14_45590	two-component sensor
YP_791795.1|gi|116049402|	7	279	2,03E-01	2,64E-01	1,08	0,22	WT	bacA mutant	PA14_45610	chemotaxis protein CheZ
YP_791797.1|gi|116049400|	4	228	9,65E-02	1,76E-01	1,31	0,38	WT	bacA mutant	PA14_45630	flagellar biosynthesis sigma factor
YP_791798.1|gi|116049399|	6	258	1,25E-01	2,01E-01	1,17	0,32	WT	bacA mutant	PA14_45640	flagellar synthesis regulator FleN
YP_791809.1|gi|116049388|	3	118	1,63E-01	2,30E-01	1,21	0,26	bacA mutant	WT	PA14_45800	flagellar motor switch protein FliM
YP_791810.1|gi|116049387|	2	59	5,67E-01	4,60E-01	1,22	0,08	WT	bacA mutant	PA14_45810	flagellar basal body-associated protein FliL
YP_791812.1|gi|116049385|	2	104	8,63E-01	5,53E-01	1,05	0,05	WT	bacA mutant	PA14_45840	hypothetical protein
YP_791819.1|gi|116049378|	3	113	7,76E-01	5,37E-01	1,49	0,06	WT	bacA mutant	PA14_45930	hypothetical protein
YP_791822.1|gi|116049375|	6	277	2,23E-01	2,81E-01	1,24	0,20	bacA mutant	WT	PA14_45960	transcriptional regulator LasR
YP_791823.1|gi|116049374|	4	145	9,25E-01	5,65E-01	1,15	0,05	WT	bacA mutant	PA14_45970	cation-transporting P-type ATPase
YP_791862.1|gi|116049335|	3	75	2,95E-01	3,34E-01	1,58	0,16	WT	bacA mutant	PA14_46450	bifunctional isocitrate dehydrogenase kinase/phosphatase
YP_791864.1|gi|116049333|	2	47	7,60E-01	5,30E-01	1,03	0,06	bacA mutant	WT	PA14_46470	erythronate-4-phosphate dehydrogenase
YP_791866.1|gi|116049331|	2	46	1,56E-01	2,25E-01	1,20	0,27	WT	bacA mutant	PA14_46490	3-oxoacyl-ACP synthase

YP_791868.1|gi|116049329|	5	276	6,91E-02	1,44E-01	1,63	0,47	WT	bacA mutant	PA14_46520	hypothetical protein
YP_791870.1|gi|116049327|	6	258	3,43E-01	3,59E-01	1,14	0,13	bacA mutant	WT	PA14_46540	hypothetical protein
YP_791878.1|gi|116049319|	2	64	3,75E-01	3,73E-01	1,36	0,12	WT	bacA mutant	PA14_46620	pyridine nucleotide-disulfide oxidoreductase
YP_791901.1|gi|116049296|	13	958	2,88E-02	9,95E-02	1,40	0,71	WT	bacA mutant	PA14_46890	short-chain dehydrogenase
YP_791903.1|gi|116049294|; YP_793551.1|gi|116053228|	20	1299	1,81E-02	8,10E-02	1,78	0,82	WT	bacA mutant	PA14_46910	ABC transporter substrate-binding protein
YP_791904.1|gi|116049293|	3	139	9,52E-01	5,72E-01	1,18	0,05	WT	bacA mutant	PA14_46920	ABC transporter permease
YP_791906.1|gi|116049291|	3	165	4,68E-02	1,20E-01	1,30	0,58	WT	bacA mutant	PA14_46950	ABC transporter ATP-binding protein
YP_791908.1|gi|116049289|	12	1102	6,57E-02	1,40E-01	1,30	0,48	WT	bacA mutant	PA14_46970	glutaminase-asparaginase
YP_791910.1|gi|116049287|	2	58	5,84E-01	4,67E-01	1,07	0,08	bacA mutant	WT	PA14_46990	two-component response regulator
YP_791918.1|gi|116049279|	19	775	2,20E-01	2,78E-01	1,15	0,20	WT	bacA mutant	PA14_47090	protease
YP_791921.1|gi|116049276|	6	298	4,47E-02	1,18E-01	1,72	0,59	WT	bacA mutant	PA14_47120	hypothetical protein
YP_791941.1|gi|116049256|	2	85	9,37E-01	5,70E-01	1,04	0,05	WT	bacA mutant	PA14_47360	oligopeptidase
YP_791951.1|gi|116049246|	2	88	1,22E-01	1,98E-01	1,53	0,32	bacA mutant	WT	PA14_47460	ribonuclease D
YP_791952.1|gi|116049245|	7	419	4,54E-01	4,16E-01	1,20	0,10	WT	bacA mutant	PA14_47490	hypothetical protein
YP_791953.1|gi|116049244|	4	199	3,68E-03	4,75E-02	1,24	0,99	WT	bacA mutant	PA14_47500	3-mercaptopyruvate sulfurtransferase
YP_791957.1|gi|116049240|	15	1366	6,68E-01	5,02E-01	1,17	0,07	bacA mutant	WT	PA14_47540	outer membrane protein
YP_791958.1|gi|116049239|	5	219	3,60E-02	1,08E-01	1,99	0,65	WT	bacA mutant	PA14_47550	glutathione peroxidase
YP_791962.1|gi|116049235|	2	81	7,37E-01	5,25E-01	1,11	0,06	WT	bacA mutant	PA14_47610	transcriptional regulator
YP_791974.1|gi|116049223|	18	1289	1,84E-01	2,49E-01	1,48	0,24	bacA mutant	WT	PA14_47800	tonB-dependent receptor
YP_791993.1|gi|116049204|	2	52	9,85E-02	1,78E-01	1,26	0,38	WT	bacA mutant	PA14_48020	L-malate dehydrogenase
YP_791996.1|gi|116049201|	4	250	3,36E-01	3,55E-01	1,31	0,14	bacA mutant	WT	PA14_48060	alkaline metalloproteinase
YP_791997.1|gi|116049200|	5	295	2,83E-01	3,26E-01	1,24	0,16	bacA mutant	WT	PA14_48090	alkaline protease secretion outer membrane protein AprF precursor
YP_792028.1|gi|116049170|	4	158	9,58E-01	5,73E-01	1,00	0,05	WT	bacA mutant	PA14_48520	membrane-bound lytic murein transglycosylase A
YP_792034.1|gi|116049164|	2	166	9,60E-02	1,75E-01	1,18	0,38	bacA mutant	WT	PA14_48590	hypothetical protein
YP_792044.1|gi|116049154|	2	65	5,53E-01	4,56E-01	1,03	0,08	WT	bacA mutant	PA14_48710	hypothetical protein

YP_792046.1|gi|116049152|	2	111	3,88E-03	4,75E-02	2,99	0,99	WT	bacA mutant	PA14_48740	hypothetical protein
YP_792048.1|gi|116049150|	8	494	3,48E-01	3,62E-01	1,19	0,13	WT	bacA mutant	PA14_48760	hydrolase
YP_792052.1|gi|116049146|	2	60	4,92E-02	1,21E-01	1,35	0,56	WT	bacA mutant	PA14_48800	hypothetical protein
YP_792057.1|gi|116049141|	3	88	4,05E-01	3,89E-01	1,07	0,11	WT	bacA mutant	PA14_48860	hypothetical protein
YP_792074.1|gi|116049124|	9	516	1,18E-02	6,76E-02	1,23	0,90	WT	bacA mutant	PA14_49030	hypothetical protein
YP_792086.1|gi|116049112|	3	215	9,62E-01	5,74E-01	1,39	0,05	WT	bacA mutant	PA14_49170	two-component sensor PhoQ
YP_792087.1|gi|116049111|	12	711	2,79E-03	4,37E-02	1,74	1,00	WT	bacA mutant	PA14_49180	two-component response regulator PhoP
YP_792088.1|gi|116049110|	13	1279	5,51E-01	4,56E-01	1,19	0,08	WT	bacA mutant	PA14_49200	PhoP/Q and low Mg2+ inducible outer membrane prote
YP_792092.1|gi|116049106|	17	682	7,38E-02	1,49E-01	1,42	0,45	bacA mutant	WT	PA14_49250	nitrate reductase catalytic subunit
YP_792095.1|gi|116049103|	6	396	7,92E-01	5,39E-01	1,02	0,06	WT	bacA mutant	PA14_49280	transglycosylase
YP_792097.1|gi|116049101|	6	373	1,60E-01	2,27E-01	2,17	0,27	WT	bacA mutant	PA14_49300	lipoxygenase
YP_792100.1|gi|116049098|	4	137	8,77E-03	6,10E-02	2,20	0,94	WT	bacA mutant	PA14_49330	hypothetical protein
YP_792104.1|gi|116049094|	2	141	4,83E-02	1,20E-01	1,38	0,57	bacA mutant	WT	PA14_49380	succinyl-diaminopimelate desuccinylase
YP_792107.1|gi|116049091|	4	268	8,76E-01	5,55E-01	1,05	0,05	WT	bacA mutant	PA14_49410	cold-shock protein
YP_792109.1|gi|116049089|	3	89	7,16E-01	5,18E-01	1,23	0,06	WT	bacA mutant	PA14_49440	two-component response regulator
YP_792110.1|gi|116049088|	14	720	9,91E-01	5,83E-01	1,06	0,05	WT	bacA mutant	PA14_49460	ribonucleotide-diphosphate reductase subunit alpha
YP_792111.1|gi|116049087|	6	452	8,95E-01	5,58E-01	1,10	0,05	WT	bacA mutant	PA14_49470	ribonucleotide-diphosphate reductase subunit beta
YP_792112.1|gi|116049086|	4	95	5,04E-01	4,35E-01	1,14	0,09	bacA mutant	WT	PA14_49480	hypothetical protein
YP_792131.1|gi|116049067|	4	125	6,34E-04	2,67E-02	2,53	1,00	WT	bacA mutant	PA14_49710	chaperone protein HchA
YP_792139.1|gi|116049059|	4	329	8,47E-03	6,03E-02	1,19	0,94	WT	bacA mutant	PA14_49800	oxidoreductase
YP_792142.1|gi|116049056|	6	227	8,32E-02	1,60E-01	1,59	0,42	WT	bacA mutant	PA14_49840	deoxyguanosinetriphosphate triphosphohydrolase
YP_792145.1|gi|116049053|	5	335	3,45E-02	1,06E-01	1,43	0,66	WT	bacA mutant	PA14_49870	peptide deformylase
YP_792148.1|gi|116049050|	6	255	4,78E-02	1,20E-01	1,50	0,57	WT	bacA mutant	PA14_49900	hypothetical protein
YP_792157.1|gi|116049041|	2	123	3,77E-01	3,74E-01	1,35	0,12	bacA mutant	WT	PA14_50010	dehydrogenase
YP_792170.1|gi|116049028|	3	121	6,26E-02	1,37E-01	1,42	0,49	bacA mutant	WT	PA14_50180	two-component response regulator
YP_792172.1|gi|116049026|	13	679	8,54E-01	5,53E-01	1,03	0,05	WT	bacA mutant	PA14_50220	transcriptional regulator FleQ

YP_792177.1|gi|116049021|	19	1945	5,27E-01	4,47E-01	1,14	0,08	bacA mutant	WT	PA14_50290	flagellin type B
YP_792185.1|gi|116049013|	2	61	5,74E-01	4,63E-01	1,21	0,08	bacA mutant	WT	PA14_50410	flagellar basal body P-ring protein
YP_792195.1|gi|116049003|	18	1289	2,77E-04	2,46E-02	2,54	1,00	WT	bacA mutant	PA14_50520	branched-chain amino acid transport protein BraC
YP_792200.1|gi|116048998|	5	336	9,00E-01	5,59E-01	1,04	0,05	bacA mutant	WT	PA14_50570	hypothetical protein
YP_792201.1|gi|116048997|	3	123	8,94E-01	5,58E-01	1,00	0,05	bacA mutant	WT	PA14_50590	HSP90 family protein
YP_792205.1|gi|116048993|	3	213	4,72E-01	4,23E-01	1,12	0,10	WT	bacA mutant	PA14_50630	hypothetical protein
YP_792208.1|gi|116048990|	5	376	4,20E-02	1,16E-01	1,49	0,61	bacA mutant	WT	PA14_50660	hypothetical protein
YP_792216.1|gi|116048982|	6	590	8,35E-01	5,50E-01	1,01	0,05	WT	bacA mutant	PA14_50740	hypothetical protein
YP_792221.1|gi|116048977|	3	139	1,41E-01	2,15E-01	1,57	0,29	WT	bacA mutant	PA14_50800	pyridoxamine 5'-phosphate oxidase
YP_792223.1|gi|116048975|	12	566	2,71E-01	3,16E-01	1,11	0,17	bacA mutant	WT	PA14_50820	esterase
YP_792229.1|gi|116048969|	7	752	1,90E-01	2,53E-01	1,42	0,23	WT	bacA mutant	PA14_50880	hypothetical protein
YP_792230.1|gi|116048968|	3	118	5,29E-01	4,47E-01	1,06	0,08	WT	bacA mutant	PA14_50890	hypothetical protein
YP_792237.1|gi|116048961|	2	61	5,75E-02	1,33E-01	1,47	0,52	WT	bacA mutant	PA14_50970	glutathione S-transferase
YP_792238.1|gi|116048960|	5	248	4,84E-02	1,20E-01	1,12	0,57	bacA mutant	WT	PA14_50980	penicillin amidase
YP_792243.1|gi|116048955|	3	95	1,12E-01	1,90E-01	1,15	0,34	bacA mutant	WT	PA14_51050	aldehyde dehydrogenase
YP_792257.1|gi|116048942|	6	256	1,87E-01	2,51E-01	1,34	0,23	WT	bacA mutant	PA14_51220	glycosyl transferase family protein
YP_792258.1|gi|116048941|	8	418	6,71E-02	1,42E-01	1,17	0,48	WT	bacA mutant	PA14_51240	phosphoribosylaminoimidazole-succinocarboxamide synthase
YP_792260.1|gi|116048939|	7	333	8,03E-02	1,56E-01	1,27	0,43	WT	bacA mutant	PA14_51260	hypothetical protein
YP_792261.1|gi|116048938|	9	441	1,11E-01	1,89E-01	1,21	0,35	WT	bacA mutant	PA14_51270	dihydrodipicolinate synthase
YP_792263.1|gi|116048936|	7	363	3,43E-02	1,06E-01	2,13	0,66	WT	bacA mutant	PA14_51290	bacterioferritin comigratory protein
YP_792266.1|gi|116048933|	6	261	8,64E-01	5,53E-01	1,02	0,05	WT	bacA mutant	PA14_51320	hypothetical protein
YP_792267.1|gi|116048932|	3	127	3,73E-01	3,73E-01	1,04	0,12	bacA mutant	WT	PA14_51330	quinolinate synthetase
YP_792271.1|gi|116048928|	8	505	6,64E-01	5,00E-01	1,05	0,07	bacA mutant	WT	PA14_51380	quinolone signal response protein
YP_792272.1|gi|116048927|	12	587	3,03E-01	3,39E-01	1,08	0,15	bacA mutant	WT	PA14_51390	3-oxoacyl-ACP synthase
YP_792273.1|gi|116048926|	11	885	3,34E-01	3,54E-01	1,10	0,14	bacA mutant	WT	PA14_51410	PqsC
YP_792274.1|gi|116048925|	7	621	8,42E-02	1,60E-01	1,26	0,42	bacA mutant	WT	PA14_51420	PqsB

YP_792275.1|gi|116048924|	2	64	3,58E-02	1,08E-01	1,79	0,65	bacA mutant	WT	PA14_51430	PqsA
YP_792300.1|gi|116048899|	4	263	5,28E-02	1,26E-01	1,52	0,54	WT	bacA mutant	PA14_51690	hypothetical protein
YP_792301.1|gi|116048898|	10	1249	7,97E-01	5,41E-01	1,03	0,06	bacA mutant	WT	PA14_51710	peptidoglycan associated lipoprotein OprL precursor
YP_792302.1|gi|116048897|	24	1592	2,36E-04	2,46E-02	1,38	1,00	WT	bacA mutant	PA14_51720	translocation protein TolB
YP_792304.1|gi|116048895|	2	60	6,87E-01	5,08E-01	1,21	0,06	WT	bacA mutant	PA14_51740	TolR protein
YP_792305.1|gi|116048894|	6	359	6,23E-01	4,84E-01	1,36	0,07	WT	bacA mutant	PA14_51750	TolQ protein
YP_792307.1|gi|116048892|	5	169	1,23E-01	1,99E-01	1,15	0,32	bacA mutant	WT	PA14_51780	Holliday junction DNA helicase RuvB
YP_792308.1|gi|116048891|	4	226	5,55E-01	4,57E-01	1,10	0,08	WT	bacA mutant	PA14_51790	Holliday junction DNA helicase RuvA
YP_792310.1|gi|116048889|	4	246	3,03E-01	3,39E-01	1,12	0,15	WT	bacA mutant	PA14_51810	hypothetical protein
YP_792311.1|gi|116048888|	25	1469	4,88E-01	4,30E-01	1,06	0,09	WT	bacA mutant	PA14_51820	aspartyl-tRNA synthetase
YP_792312.1|gi|116048887|	7	374	2,50E-02	9,43E-02	1,29	0,74	WT	bacA mutant	PA14_51830	DNA-binding stress protein
YP_792313.1|gi|116048886|	2	103	6,45E-01	4,92E-01	1,07	0,07	bacA mutant	WT	PA14_51840	cold-shock protein
YP_792315.1|gi|116048884|	5	192	4,30E-03	4,75E-02	1,90	0,99	WT	bacA mutant	PA14_51860	hypothetical protein
YP_792316.1|gi|116048883|	21	2380	6,72E-01	5,04E-01	1,12	0,06	bacA mutant	WT	PA14_51880	basic amino acid, basic peptide and imipenem outer membrane porin OprD precursor
YP_792318.1|gi|116048881|	16	881	4,51E-02	1,19E-01	1,14	0,59	WT	bacA mutant	PA14_51900	prolyl-tRNA synthetase
YP_792321.1|gi|116048878|	3	179	5,32E-01	4,48E-01	1,06	0,08	WT	bacA mutant	PA14_51930	thioredoxin
YP_792325.1|gi|116048874|	3	144	8,57E-02	1,62E-01	1,51	0,41	WT	bacA mutant	PA14_51980	arsenate reductase
YP_792328.1|gi|116048871|	2	63	1,16E-01	1,94E-01	1,48	0,34	WT	bacA mutant	PA14_52010	DNA replication initiation factor
YP_792329.1|gi|116048870|	6	329	4,63E-02	1,20E-01	1,47	0,58	WT	bacA mutant	PA14_52020	hypothetical protein
YP_792330.1|gi|116048869|	4	183	6,06E-01	4,76E-01	1,03	0,07	WT	bacA mutant	PA14_52040	phosphoribosylaminoimidazole synthetase
YP_792332.1|gi|116048867|	9	407	3,30E-03	4,75E-02	1,82	0,99	WT	bacA mutant	PA14_52060	hypothetical protein
YP_792338.1|gi|116048861|	5	164	2,48E-01	3,00E-01	1,56	0,18	bacA mutant	WT	PA14_52130	hypothetical protein
YP_792339.1|gi|116048860|	2	142	1,11E-01	1,89E-01	1,22	0,35	WT	bacA mutant	PA14_52140	hypothetical protein
YP_792340.1|gi|116048859|	9	432	4,75E-01	4,24E-01	1,29	0,09	bacA mutant	WT	PA14_52150	lipopolysaccharide biosynthetic protein LpxO2
YP_792341.1|gi|116048858|	3	93	2,08E-01	2,69E-01	1,14	0,21	bacA mutant	WT	PA14_52160	nucleoside triphosphate pyrophosphohydrolase
YP_792342.1|gi|116048857|	7	295	4,78E-02	1,20E-01	1,47	0,57	WT	bacA mutant	PA14_52180	GTP pyrophosphokinase

YP_792343.1|gi|116048856|	4	131	1,46E-01	2,18E-01	1,15	0,28	bacA mutant	WT	PA14_52190	23S rRNA 5-methyluridine methyltransferase
YP_792344.1|gi|116048855|	2	75	3,19E-01	3,48E-01	1,28	0,14	WT	bacA mutant	PA14_52210	cysteine synthase B
YP_792349.1|gi|116048850|	4	326	1,89E-01	2,53E-01	1,28	0,23	bacA mutant	WT	PA14_52270	D-lactate dehydrogenase
YP_792357.1|gi|116048842|	3	92	4,65E-01	4,20E-01	1,27	0,10	WT	bacA mutant	PA14_52370	hypothetical protein
YP_792360.1|gi|116048839|	2	59	1,71E-01	2,36E-01	1,50	0,25	WT	bacA mutant	PA14_52420	ribosomal protein S12 methylthiotransferase
YP_792372.1|gi|116048828|	9	559	3,52E-01	3,64E-01	1,18	0,13	WT	bacA mutant	PA14_52580	aspartate kinase
YP_792373.1|gi|116048827|	6	299	3,88E-02	1,12E-01	1,73	0,63	WT	bacA mutant	PA14_52600	alanyl-tRNA synthetase
YP_792375.1|gi|116048825|	7	347	3,92E-01	3,82E-01	1,16	0,12	WT	bacA mutant	PA14_52630	succinylglutamate desuccinylase
YP_792376.1|gi|116048824|	2	103	1,75E-01	2,40E-01	1,18	0,25	WT	bacA mutant	PA14_52640	hypothetical protein
YP_792377.1|gi|116048823|	5	297	3,49E-01	3,63E-01	1,09	0,13	WT	bacA mutant	PA14_52660	succinylarginine dihydrolase
YP_792378.1|gi|116048822|	6	312	1,37E-02	7,10E-02	1,58	0,87	bacA mutant	WT	PA14_52670	succinylglutamic semialdehyde dehydrogenase
YP_792379.1|gi|116048821|	6	213	1,30E-01	2,07E-01	1,21	0,31	WT	bacA mutant	PA14_52690	arginine/ornithine succinyltransferase AII subunit
YP_792380.1|gi|116048820|	4	343	5,24E-01	4,46E-01	1,12	0,08	bacA mutant	WT	PA14_52700	arginine/ornithine succinyltransferase AI subunit
YP_792381.1|gi|116048819|	16	1122	4,93E-01	4,32E-01	1,10	0,09	bacA mutant	WT	PA14_52720	bifunctional N-succinyldiaminopimelate-aminotransferase/acetylornithine transaminase
YP_792383.1|gi|116048817|	4	260	3,74E-01	3,73E-01	1,21	0,12	bacA mutant	WT	PA14_52740	transcriptional regulator ArgR
YP_792384.1|gi|116048816|	3	122	1,55E-01	2,25E-01	1,34	0,27	WT	bacA mutant	PA14_52750	arginine/ornithine transport protein AotP
YP_792385.1|gi|116048815|	5	311	9,69E-01	5,76E-01	1,00	0,05	bacA mutant	WT	PA14_52760	hypothetical protein
YP_792388.1|gi|116048812|	12	848	4,25E-02	1,16E-01	1,48	0,60	WT	bacA mutant	PA14_52790	arginine/ornithine binding protein AotJ
YP_792389.1|gi|116048811|	5	300	1,76E-01	2,40E-01	1,33	0,25	bacA mutant	WT	PA14_52800	acetyl-CoA synthetase
YP_792404.1|gi|116048796|	5	231	4,52E-01	4,15E-01	1,13	0,10	bacA mutant	WT	PA14_52990	phenylalanine 4-monooxygenase
YP_792405.1|gi|116048795|	9	444	3,04E-01	3,39E-01	1,07	0,15	bacA mutant	WT	PA14_53000	pterin-4-alpha-carbinolamine dehydratase
YP_792406.1|gi|116048794|	22	1475	9,45E-04	2,94E-02	1,26	1,00	bacA mutant	WT	PA14_53010	aromatic amino acid aminotransferase
YP_792409.1|gi|116048791|	6	353	4,11E-03	4,75E-02	1,62	0,99	WT	bacA mutant	PA14_53040	lysozyme inhibitor
YP_792411.1|gi|116048789|	20	1635	6,30E-01	4,86E-01	1,02	0,07	bacA mutant	WT	PA14_53070	4-hydroxyphenylpyruvate dioxygenase
YP_792414.1|gi|116048786|	2	97	5,43E-01	4,52E-01	1,11	0,08	WT	bacA mutant	PA14_53110	oxidoreductase
YP_792421.1|gi|116048779|	8	533	1,07E-02	6,67E-02	1,80	0,91	WT	bacA mutant	PA14_53200	hypothetical protein

YP_792423.1|gi|116048777|	21	1423	3,60E-01	3,67E-01	1,12	0,13	bacA mutant	WT	PA14_53220	fumarate hydratase
YP_792424.1|gi|116048776|	3	209	4,13E-02	1,16E-01	1,38	0,61	WT	bacA mutant	PA14_53230	oxidoreductase
YP_792425.1|gi|116048775|	16	1536	2,89E-02	9,95E-02	1,58	0,71	WT	bacA mutant	PA14_53250	chitin-binding protein CbpD
YP_792429.1|gi|116048771|	4	254	4,93E-02	1,21E-01	1,38	0,56	bacA mutant	WT	PA14_53300	alkyl hydroperoxide reductase
YP_792439.1|gi|116048761|	3	197	2,98E-02	1,00E-01	1,51	0,70	WT	bacA mutant	PA14_53420	glutathione peroxidase
YP_792442.1|gi|116048758|	9	455	2,98E-03	4,49E-02	1,10	1,00	WT	bacA mutant	PA14_53470	acetate kinase
YP_792443.1|gi|116048757|	15	894	5,30E-01	4,47E-01	1,07	0,08	bacA mutant	WT	PA14_53480	phosphate acetyltransferase
YP_792445.1|gi|116048755|	8	595	3,86E-02	1,12E-01	1,78	0,63	WT	bacA mutant	PA14_53500	hypothetical protein
YP_792454.1|gi|116048746|	11	407	5,04E-01	4,35E-01	1,13	0,09	bacA mutant	WT	PA14_53590	hypothetical protein
YP_792475.1|gi|116048725|	6	393	6,58E-01	4,97E-01	1,06	0,07	bacA mutant	WT	PA14_53820	hypothetical protein
YP_792478.1|gi|116048722|	2	139	3,19E-02	1,04E-01	1,38	0,68	WT	bacA mutant	PA14_53850	oxidoreductase
YP_792486.1|gi|116048714|	11	753	4,86E-03	4,75E-02	1,64	0,98	WT	bacA mutant	PA14_53940	2-methylisocitrate lyase
YP_792487.1|gi|116048713|	9	783	4,15E-02	1,16E-01	1,44	0,61	WT	bacA mutant	PA14_53950	methylcitrate synthase
YP_792488.1|gi|116048712|	10	490	8,29E-01	5,49E-01	1,06	0,05	WT	bacA mutant	PA14_53970	aconitate hydratase
YP_792489.1|gi|116048711|	3	107	1,16E-02	6,72E-02	1,22	0,90	WT	bacA mutant	PA14_53980	hypothetical protein
YP_792490.1|gi|116048710|	10	727	8,88E-01	5,57E-01	1,02	0,05	WT	bacA mutant	PA14_54000	2-methylcitrate dehydratase
YP_792501.1|gi|116048699|	3	178	7,08E-01	5,17E-01	1,07	0,06	WT	bacA mutant	PA14_54150	sodium/proline symporter PutP
YP_792502.1|gi|116048698|	20	1040	1,35E-01	2,10E-01	1,22	0,30	WT	bacA mutant	PA14_54170	bifunctional proline dehydrogenase/pyrroline-5-carboxylate dehydrogenase
YP_792505.1|gi|116048695|	18	927	1,17E-01	1,95E-01	1,35	0,33	bacA mutant	WT	PA14_54210	ATP-dependent protease
YP_792506.1|gi|116048694|	2	118	1,57E-01	2,26E-01	1,26	0,27	bacA mutant	WT	PA14_54220	inhibitor of cysteine peptidase
YP_792511.1|gi|116048689|	7	612	9,49E-01	5,72E-01	1,04	0,05	bacA mutant	WT	PA14_54290	pyridoxine 5'-phosphate synthase
YP_792513.1|gi|116048687|	5	172	1,48E-02	7,44E-02	1,29	0,86	WT	bacA mutant	PA14_54320	GTP-binding protein Era
YP_792514.1|gi|116048686|	3	103	9,96E-01	5,84E-01	1,00	0,05	WT	bacA mutant	PA14_54330	ribonuclease III
YP_792516.1|gi|116048684|	9	355	9,62E-01	5,74E-01	1,13	0,05	WT	bacA mutant	PA14_54350	signal peptidase I
YP_792517.1|gi|116048683|	8	343	1,58E-01	2,26E-01	1,14	0,27	WT	bacA mutant	PA14_54370	GTP-binding protein LepA
YP_792518.1|gi|116048682|	15	941	3,33E-03	4,75E-02	1,36	0,99	WT	bacA mutant	PA14_54390	serine protease MucD

YP_792520.1|gi|116048680|	10	565	1,71E-02	7,88E-02	1,55	0,83	WT	bacA mutant	PA14_54410	negative regulator for alginate biosynthesis MucB
YP_792521.1|gi|116048679|	4	255	6,95E-02	1,44E-01	1,36	0,47	WT	bacA mutant	PA14_54420	anti-sigma factor MucA
YP_792522.1|gi|116048678|	8	507	4,26E-02	1,16E-01	1,15	0,60	WT	bacA mutant	PA14_54430	RNA polymerase sigma factor AlgU
YP_792523.1|gi|116048677|	5	310	3,05E-02	1,01E-01	1,33	0,69	WT	bacA mutant	PA14_54450	L-aspartate oxidase
YP_792526.1|gi|116048674|	9	671	4,49E-01	4,13E-01	1,08	0,10	bacA mutant	WT	PA14_54480	hypothetical protein
YP_792527.1|gi|116048673|	4	259	3,33E-01	3,54E-01	1,12	0,14	WT	bacA mutant	PA14_54490	hypothetical protein
YP_792530.1|gi|116048670|	4	175	3,28E-01	3,52E-01	2,03	0,14	bacA mutant	WT	PA14_54520	porin
YP_792538.1|gi|116048662|	7	610	6,53E-04	2,67E-02	1,29	1,00	WT	bacA mutant	PA14_54620	aldehyde dehydrogenase
YP_792539.1|gi|116048661|	10	597	9,59E-01	5,73E-01	1,03	0,05	WT	bacA mutant	PA14_54630	acyl-CoA dehydrogenase
YP_792540.1|gi|116048660|	16	854	7,89E-03	5,95E-02	1,68	0,95	WT	bacA mutant	PA14_54640	enoyl-CoA hydratase
YP_792541.1|gi|116048659|	11	554	7,55E-01	5,29E-01	1,04	0,06	WT	bacA mutant	PA14_54660	enoyl-CoA hydratase/isomerase
YP_792544.1|gi|116048656|	2	117	6,22E-02	1,37E-01	1,69	0,50	WT	bacA mutant	PA14_54690	hypothetical protein
YP_792579.1|gi|116048621|	4	178	9,07E-01	5,60E-01	1,30	0,05	WT	bacA mutant	PA14_55080	hypothetical protein
YP_792590.1|gi|116048611|	7	301	1,41E-02	7,23E-02	1,21	0,87	bacA mutant	WT	PA14_55200	amidase
YP_792653.1|gi|116052342|	12	589	7,50E-01	5,28E-01	1,02	0,06	bacA mutant	WT	PA14_55980	hypothetical protein
YP_792654.1|gi|116052343|;<br>YP_792652.1|gi|116052341|;<br>YP_790540.1|gi|116050641|	13	836	8,69E-01	5,55E-01	1,04	0,05	WT	bacA mutant	PA14_56000	chemotactic transducer PctA
YP_792655.1|gi|116052344|	3	152	6,56E-01	4,96E-01	1,04	0,07	bacA mutant	WT	PA14_56010	chemotactic transducer PctB
YP_792657.1|gi|116052346|	2	112	7,56E-02	1,51E-01	1,45	0,44	bacA mutant	WT	PA14_56040	hypothetical protein
YP_792659.1|gi|116052348|	4	223	5,37E-01	4,50E-01	1,07	0,08	WT	bacA mutant	PA14_56060	formyltetrahydrofolate deformylase
YP_792660.1|gi|116052349|	10	547	1,35E-01	2,10E-01	1,26	0,30	WT	bacA mutant	PA14_56070	transcriptional regulator MvaT, P16 subunit
YP_792661.1|gi|116052350|	8	336	1,57E-01	2,26E-01	1,11	0,27	bacA mutant	WT	PA14_56080	exonuclease I
YP_792662.1|gi|116052351|	2	94	2,70E-01	3,16E-01	1,68	0,17	bacA mutant	WT	PA14_56090	hypothetical protein
YP_792673.1|gi|116052362|	11	683	6,93E-01	5,11E-01	1,03	0,06	WT	bacA mutant	PA14_56220	hypothetical protein
YP_792674.1|gi|116052363|	26	1821	1,81E-01	2,46E-01	1,08	0,24	bacA mutant	WT	PA14_56240	pyruvate kinase
YP_792678.1|gi|116052367|	8	477	2,49E-01	3,00E-01	1,14	0,18	WT	bacA mutant	PA14_56300	fumarase

YP_792681.1|gi|116052370|	3	225	1,96E-01	2,58E-01	1,14	0,22	WT	bacA mutant	PA14_56370	hypothetical protein
YP_792690.1|gi|116052379|	4	223	1,11E-02	6,67E-02	2,68	0,91	WT	bacA mutant	PA14_56510	hypothetical protein
YP_792693.1|gi|116052382|	3	97	2,28E-01	2,84E-01	1,19	0,20	WT	bacA mutant	PA14_56540	hypothetical protein
YP_792694.1|gi|116052383|	3	156	1,63E-01	2,30E-01	1,35	0,26	bacA mutant	WT	PA14_56550	hypothetical protein
YP_792697.1|gi|116052386|	18	1503	2,23E-01	2,81E-01	1,21	0,20	WT	bacA mutant	PA14_56590	hypothetical protein
YP_792703.1|gi|116052392|	2	81	6,73E-01	5,04E-01	1,69	0,06	WT	bacA mutant	PA14_56680	ferrous iron transport protein B
YP_792706.1|gi|116052395|	5	192	2,21E-02	9,25E-02	1,42	0,77	WT	bacA mutant	PA14_56720	oxidoreductase
YP_792707.1|gi|116052396|	11	566	4,88E-02	1,20E-01	1,19	0,56	WT	bacA mutant	PA14_56730	hypothetical protein
YP_792711.1|gi|116052400|	6	378	2,37E-02	9,30E-02	2,21	0,76	WT	bacA mutant	PA14_56780	superoxide dismutase
YP_792712.1|gi|116052401|	2	51	6,76E-01	5,05E-01	1,07	0,06	WT	bacA mutant	PA14_56790	diguanylate cyclase
YP_792714.1|gi|116052403|	2	128	9,02E-01	5,59E-01	1,00	0,05	WT	bacA mutant	PA14_56810	lemA-like protein
YP_792715.1|gi|116052404|	25	1936	2,48E-02	9,43E-02	1,38	0,75	WT	bacA mutant	PA14_56830	metalloproteinase outer membrane
YP_792717.1|gi|116052406|	13	786	5,62E-01	4,59E-01	1,05	0,08	WT	bacA mutant	PA14_56850	lipoprotein
YP_792719.1|gi|116052408|	3	168	2,78E-01	3,22E-01	1,37	0,16	bacA mutant	WT	PA14_56880	membrane fusion protein
YP_792721.1|gi|116052410|;<br>YP_793387.1|gi|116053068|	4	162	8,56E-01	5,53E-01	1,06	0,05	WT	bacA mutant	PA14_56900	nicotinate phosphoribosyltransferase
YP_792731.1|gi|116052420|	49	4716	1,17E-01	1,95E-01	1,24	0,33	WT	bacA mutant	PA14_57010	chaperonin GroEL
YP_792732.1|gi|116052421|	5	345	2,17E-01	2,76E-01	1,41	0,21	WT	bacA mutant	PA14_57020	co-chaperonin GroES
YP_792734.1|gi|116052423|	4	212	2,62E-01	3,09E-01	1,10	0,17	bacA mutant	WT	PA14_57040	hypothetical protein
YP_792735.1|gi|116052424|	8	347	1,32E-02	6,92E-02	1,64	0,88	WT	bacA mutant	PA14_57050	3-ketoacyl-ACP reductase
YP_792736.1|gi|116052425|	9	518	6,46E-01	4,92E-01	1,15	0,07	bacA mutant	WT	PA14_57060	hypothetical protein
YP_792740.1|gi|116052429|	7	778	8,17E-01	5,46E-01	1,06	0,05	WT	bacA mutant	PA14_57110	hypothetical protein
YP_792741.1|gi|116052430|	9	437	4,84E-02	1,20E-01	1,60	0,57	WT	bacA mutant	PA14_57130	nucleotide-binding protein
YP_792743.1|gi|116052432|	2	104	1,48E-01	2,19E-01	1,50	0,28	WT	bacA mutant	PA14_57160	2-dehydropantoate 2-reductase
YP_792745.1|gi|116052434|	5	229	3,41E-01	3,59E-01	1,08	0,13	WT	bacA mutant	PA14_57180	hypothetical protein
YP_792746.1|gi|116052435|;<br>YP_793307.1|gi|116052989|	5	338	6,96E-01	5,12E-01	1,02	0,06	WT	bacA mutant	PA14_57190	hypothetical protein

YP_792748.1|gi|116052437|	10	744	2,71E-01	3,16E-01	1,10	0,17	WT	bacA mutant	PA14_57210	bifunctional ornithine acetyltransferase/N-acetylglutamate synthase
YP_792749.1|gi|116052438|	14	617	1,28E-01	2,04E-01	1,56	0,31	WT	bacA mutant	PA14_57220	preprotein translocase subunit SecA
YP_792752.1|gi|116052441|	11	675	7,40E-03	5,86E-02	1,26	0,96	WT	bacA mutant	PA14_57260	UDP-3-O-[3-hydroxymyristoyl] N-acetylglucosamine deacetylase
YP_792753.1|gi|116054417|	13	755	3,75E-02	1,11E-01	1,26	0,64	WT	bacA mutant	PA14_57275	cell division protein FtsZ
YP_792754.1|gi|116052442|	16	1044	8,18E-02	1,58E-01	1,11	0,42	WT	bacA mutant	PA14_57290	cell division protein FtsA
YP_792756.1|gi|116052444|	7	300	5,59E-01	4,58E-01	1,09	0,08	WT	bacA mutant	PA14_57320	D-alanine--D-alanine ligase
YP_792757.1|gi|116052445|	7	430	4,48E-01	4,12E-01	1,09	0,10	bacA mutant	WT	PA14_57330	UDP-N-acetylmuramate--L-alanine ligase
YP_792758.1|gi|116052446|	9	454	1,19E-01	1,96E-01	1,36	0,33	bacA mutant	WT	PA14_57340	UDPdiphospho-muramoylpentapeptide beta-N-acetylglucosaminyltransferase
YP_792760.1|gi|116052448|	14	633	8,32E-02	1,60E-01	1,09	0,42	WT	bacA mutant	PA14_57370	UDP-N-acetylmuramoyl-L-alanyl-D-glutamate synthetase
YP_792762.1|gi|116052450|	10	631	6,80E-01	5,06E-01	1,04	0,06	bacA mutant	WT	PA14_57390	UDP-N-acetylmuramoylalanyl-D-glutamyl-2,6- diaminopimelate--D-alanyl-D-alanine ligase
YP_792763.1|gi|116052451|	13	825	4,49E-01	4,13E-01	1,06	0,10	bacA mutant	WT	PA14_57410	UDP-N-acetylmuramoylalanyl-D-glutamate--2, 6-diaminopimelate ligase
YP_792766.1|gi|116052453|	9	704	9,26E-01	5,65E-01	1,00	0,05	bacA mutant	WT	PA14_57450	S-adenosyl-methyltransferase MraW
YP_792768.1|gi|116052455|	4	107	8,83E-01	5,56E-01	1,04	0,05	WT	bacA mutant	PA14_57470	hypothetical protein
YP_792769.1|gi|116052456|	16	1181	7,23E-01	5,19E-01	1,03	0,06	WT	bacA mutant	PA14_57480	lipoprotein
YP_792771.1|gi|116052458|	6	511	4,22E-03	4,75E-02	1,99	0,99	WT	bacA mutant	PA14_57500	phosphoheptose isomerase
YP_792772.1|gi|116052459|	6	390	1,04E-01	1,83E-01	1,39	0,36	WT	bacA mutant	PA14_57510	secreted lipoprotein
YP_792774.1|gi|116052461|	3	184	1,00E-01	1,80E-01	1,16	0,37	WT	bacA mutant	PA14_57530	stringent starvation protein A
YP_792775.1|gi|116052462|	12	737	7,64E-01	5,32E-01	1,09	0,06	WT	bacA mutant	PA14_57540	cytochrome c1
YP_792776.1|gi|116052463|	7	383	7,29E-01	5,22E-01	1,03	0,06	bacA mutant	WT	PA14_57560	cytochrome b
YP_792777.1|gi|116052464|	7	377	1,80E-02	8,08E-02	1,60	0,82	WT	bacA mutant	PA14_57570	cytochrome c reductase, iron-sulfur subun
YP_792778.1|gi|116052465|	4	201	8,80E-01	5,55E-01	1,10	0,05	bacA mutant	WT	PA14_57580	30S ribosomal protein S9
YP_792779.1|gi|116052466|	6	444	9,18E-01	5,63E-01	1,00	0,05	WT	bacA mutant	PA14_57590	50S ribosomal protein L13
YP_792780.1|gi|116052467|	4	217	8,96E-01	5,59E-01	1,00	0,05	bacA mutant	WT	PA14_57600	oxidoreductase
YP_792783.1|gi|116052470|	2	66	2,61E-01	3,09E-01	1,20	0,17	bacA mutant	WT	PA14_57650	hypothetical protein
YP_792784.1|gi|116052471|	9	643	1,04E-01	1,83E-01	1,19	0,36	bacA mutant	WT	PA14_57670	tryptophanyl-tRNA synthetase
YP_792785.1|gi|116052472|	3	124	5,80E-03	5,27E-02	1,90	0,97	bacA mutant	WT	PA14_57680	hypothetical protein

YP_792786.1|gi|116052473|	9	847	6,28E-01	4,86E-01	1,06	0,07	WT	bacA mutant	PA14_57690	hypothetical protein
YP_792787.1|gi|116052474|	15	874	3,52E-01	3,64E-01	1,22	0,13	WT	bacA mutant	PA14_57710	bifunctional sulfate adenylyltransferase subunit 1/adenylylsulfate kinase
YP_792788.1|gi|116052475|	7	304	1,50E-01	2,21E-01	1,20	0,28	WT	bacA mutant	PA14_57720	sulfate adenylyltransferase subunit 2
YP_792790.1|gi|116052477|	5	219	4,15E-02	1,16E-01	1,38	0,61	WT	bacA mutant	PA14_57740	hypothetical protein
YP_792791.1|gi|116052478|	7	303	7,02E-01	5,15E-01	1,09	0,06	WT	bacA mutant	PA14_57760	AlgW protein
YP_792792.1|gi|116052479|	4	148	2,91E-01	3,32E-01	1,15	0,16	bacA mutant	WT	PA14_57770	histidinol-phosphate aminotransferase
YP_792793.1|gi|116052480|	10	775	1,17E-01	1,95E-01	1,06	0,33	WT	bacA mutant	PA14_57780	histidinol dehydrogenase
YP_792794.1|gi|116052481|	3	143	6,43E-01	4,92E-01	1,08	0,07	WT	bacA mutant	PA14_57800	ATP phosphoribosyltransferase
YP_792795.1|gi|116052482|	21	1197	1,63E-01	2,30E-01	1,13	0,26	WT	bacA mutant	PA14_57810	UDP-N-acetylglucosamine 1-carboxyvinyltransferase
YP_792798.1|gi|116052485|	8	355	2,84E-02	9,88E-02	1,91	0,71	WT	bacA mutant	PA14_57840	hypothetical protein
YP_792799.1|gi|116052486|	5	309	4,96E-01	4,33E-01	1,15	0,09	bacA mutant	WT	PA14_57850	hypothetical protein
YP_792800.1|gi|116052487|	2	79	8,05E-01	5,43E-01	1,08	0,05	WT	bacA mutant	PA14_57870	ABC transporter permease
YP_792801.1|gi|116052488|	2	64	1,22E-01	1,98E-01	1,27	0,32	bacA mutant	WT	PA14_57880	ABC transporter ATP-binding protein
YP_792802.1|gi|116052489|	3	139	7,96E-01	5,41E-01	1,01	0,06	bacA mutant	WT	PA14_57890	hypothetical protein
YP_792804.1|gi|116052491|	3	169	4,43E-01	4,09E-01	1,15	0,10	WT	bacA mutant	PA14_57910	hypothetical protein
YP_792805.1|gi|116052492|	4	203	2,65E-02	9,67E-02	1,46	0,73	WT	bacA mutant	PA14_57920	hypothetical protein
YP_792806.1|gi|116052493|	5	234	7,10E-02	1,45E-01	1,23	0,46	WT	bacA mutant	PA14_57930	ABC transporter ATP-binding protein
YP_792807.1|gi|116052494|	9	442	8,92E-01	5,58E-01	1,01	0,05	bacA mutant	WT	PA14_57940	RNA polymerase factor sigma-54
YP_792809.1|gi|116052496|	4	326	3,66E-01	3,71E-01	1,08	0,13	bacA mutant	WT	PA14_57960	nitrogen regulatory IIA protein
YP_792810.1|gi|116052497|	7	517	2,13E-01	2,73E-01	1,16	0,21	WT	bacA mutant	PA14_57970	hypothetical protein
YP_792813.1|gi|116052500|	5	352	1,60E-02	7,70E-02	1,54	0,84	WT	bacA mutant	PA14_58000	superoxide dismutase
YP_792815.1|gi|116052502|	8	501	2,73E-01	3,17E-01	1,19	0,17	bacA mutant	WT	PA14_58030	fumarate hydratase
YP_792817.1|gi|116052504|	14	789	4,90E-01	4,31E-01	1,03	0,09	WT	bacA mutant	PA14_58050	PmbA protein
YP_792818.1|gi|116052505|	3	166	8,35E-01	5,50E-01	1,03	0,05	WT	bacA mutant	PA14_58060	hypothetical protein
YP_792819.1|gi|116052506|	8	468	7,53E-01	5,28E-01	1,04	0,06	WT	bacA mutant	PA14_58070	hypothetical protein
YP_792820.1|gi|116052507|	3	162	1,89E-03	3,93E-02	1,59	1,00	WT	bacA mutant	PA14_58080	hypothetical protein

YP_792822.1|gi|116052509|	5	325	7,45E-01	5,27E-01	1,04	0,06	bacA mutant	WT	PA14_58100	cytoplasmic axial filament protein
YP_792825.1|gi|116052512|	3	146	8,40E-01	5,51E-01	1,06	0,05	WT	bacA mutant	PA14_58130	rod shape-determining protein MreC
YP_792826.1|gi|116052513|	18	1062	5,04E-01	4,35E-01	1,04	0,09	WT	bacA mutant	PA14_58150	rod shape-determining protein MreB
YP_792828.1|gi|116052515|	14	770	2,86E-01	3,28E-01	1,09	0,16	bacA mutant	WT	PA14_58180	aspartyl/glutamyl-tRNA amidotransferase subunit A
YP_792829.1|gi|116052516|	19	914	8,01E-02	1,56E-01	1,16	0,43	WT	bacA mutant	PA14_58190	aspartyl/glutamyl-tRNA amidotransferase subunit B
YP_792834.1|gi|116052521|	2	106	4,95E-01	4,33E-01	1,14	0,09	bacA mutant	WT	PA14_58250	hypothetical protein
YP_792836.1|gi|116052523|	8	430	9,76E-01	5,78E-01	1,01	0,05	WT	bacA mutant	PA14_58270	hypothetical protein
YP_792838.1|gi|116052525|	4	313	6,26E-01	4,86E-01	1,07	0,07	WT	bacA mutant	PA14_58300	two-component response regulator
YP_792840.1|gi|116052527|	13	1119	2,42E-03	4,16E-02	2,42	1,00	WT	bacA mutant	PA14_58330	hypothetical protein
YP_792841.1|gi|116052528|	5	314	1,39E-03	3,63E-02	1,95	1,00	WT	bacA mutant	PA14_58350	dipeptide ABC transporter substrate-binding protein DppA1
YP_792842.1|gi|116052529|	3	209	5,84E-01	4,67E-01	1,11	0,08	bacA mutant	WT	PA14_58360	dipeptide ABC transporter substrate-binding protein DppA2
YP_792843.1|gi|116054453|	11	538	1,14E-01	1,92E-01	1,09	0,34	bacA mutant	WT	PA14_58375	metallopeptidase MdpA
YP_792845.1|gi|116052531|	16	837	1,23E-02	6,84E-02	1,48	0,89	WT	bacA mutant	PA14_58390	dipeptide ABC transporter substrate-binding protein DppA3
YP_792846.1|gi|116052532|	15	1425	4,40E-01	4,08E-01	1,17	0,10	bacA mutant	WT	PA14_58410	glycine-glutamate dipeptide porin OpdP
YP_792847.1|gi|116052533|	7	329	5,00E-01	4,34E-01	1,09	0,09	bacA mutant	WT	PA14_58420	dipeptide ABC transporter substrate-binding protein DppA4
YP_792848.1|gi|116052534|	2	72	9,88E-01	5,82E-01	1,18	0,05	WT	bacA mutant	PA14_58440	dipeptide ABC transporter permease DppB
YP_792849.1|gi|116052535|	4	140	9,61E-01	5,73E-01	1,22	0,05	WT	bacA mutant	PA14_58450	dipeptide ABC transporter permease DppC
YP_792850.1|gi|116052536|;<br>YP_793861.1|gi|116053534|	7	281	1,57E-01	2,26E-01	1,34	0,27	WT	bacA mutant	PA14_58470	dipeptide ABC transporter ATP-binding protein DppD
YP_792851.1|gi|116052537|	5	426	4,77E-01	4,25E-01	1,14	0,09	WT	bacA mutant	PA14_58490	dipeptide ABC transporter ATP-binding protein DppF
YP_792859.1|gi|116052544|	8	394	2,84E-02	9,88E-02	1,62	0,71	WT	bacA mutant	PA14_58570	outer membrane ferric siderophore receptor
YP_792864.1|gi|116052549|	11	735	6,50E-02	1,40E-01	1,27	0,48	WT	bacA mutant	PA14_58630	ornithine decarboxylase
YP_792869.1|gi|116052554|	4	120	1,41E-01	2,15E-01	1,30	0,29	WT	bacA mutant	PA14_58700	nicotinate-nucleotide pyrophosphorylase
YP_792871.1|gi|116052556|	6	661	6,68E-01	5,02E-01	1,08	0,07	WT	bacA mutant	PA14_58730	type IV pilin structural subunit
YP_792873.1|gi|116052558|	7	381	2,72E-01	3,16E-01	1,10	0,17	bacA mutant	WT	PA14_58750	type 4 fimbrial biogenesis protein PilB
YP_792874.1|gi|116052559|	2	36	5,63E-01	4,59E-01	1,07	0,08	bacA mutant	WT	PA14_58760	type 4 fimbrial biogenesis protein pilC

YP_792876.1|gi|116052561|	2	158	4,60E-01	4,17E-01	1,13	0,10	WT	bacA mutant	PA14_58780	dephospho-CoA kinase
YP_792882.1|gi|116052567|	2	89	9,88E-02	1,78E-01	1,39	0,38	WT	bacA mutant	PA14_58840	hypothetical protein
YP_792945.1|gi|116052630|	2	61	8,22E-01	5,48E-01	1,02	0,05	WT	bacA mutant	PA14_59550	hypothetical protein
YP_792971.1|gi|116052656|	12	1410	3,16E-02	1,03E-01	1,70	0,68	WT	bacA mutant	PA14_59840	hypothetical protein
YP_792998.1|gi|116052682|	2	57	5,59E-01	4,58E-01	1,06	0,08	bacA mutant	WT	PA14_60100	deoxycytidine triphosphate deaminase
YP_792999.1|gi|116052683|	2	60	1,13E-01	1,91E-01	1,67	0,34	bacA mutant	WT	PA14_60110	hypothetical protein
YP_793003.1|gi|116052687|	29	1959	3,42E-01	3,59E-01	1,30	0,13	bacA mutant	WT	PA14_60190	clpB protein
YP_793005.1|gi|116052689|	2	97	5,53E-01	4,56E-01	1,07	0,08	bacA mutant	WT	PA14_60210	pseudouridine synthase
YP_793006.1|gi|116052690|	10	495	6,29E-02	1,38E-01	1,29	0,49	WT	bacA mutant	PA14_60230	competence protein ComL
YP_793009.1|gi|116052693|	10	622	9,32E-01	5,68E-01	1,00	0,05	bacA mutant	WT	PA14_60260	two-component response regulator PilR
YP_793010.1|gi|116052694|	2	116	3,31E-01	3,53E-01	1,13	0,14	WT	bacA mutant	PA14_60270	glycine/D-amino acid oxidases
YP_793016.1|gi|116052700|	5	193	3,68E-02	1,09E-01	1,11	0,64	bacA mutant	WT	PA14_60330	4-hydroxy-3-methylbut-2-enyl diphosphate reductase
YP_793019.1|gi|116052703|	10	473	3,42E-01	3,59E-01	1,22	0,13	WT	bacA mutant	PA14_60370	isoleucyl-tRNA synthetase
YP_793020.1|gi|116052704|	4	177	9,35E-01	5,69E-01	1,01	0,05	bacA mutant	WT	PA14_60380	bifunctional riboflavin kinase/FMN adenylyltransferase
YP_793024.1|gi|116052708|	6	548	3,28E-01	3,52E-01	1,14	0,14	bacA mutant	WT	PA14_60420	gamma-glutamyl kinase
YP_793025.1|gi|116054395|	10	787	9,17E-01	5,63E-01	1,01	0,05	WT	bacA mutant	PA14_60445	GTPase ObgE
YP_793026.1|gi|116052709|	3	101	2,48E-01	3,00E-01	1,48	0,18	bacA mutant	WT	PA14_60450	50S ribosomal protein L27
YP_793027.1|gi|116052710|	7	449	2,50E-01	3,01E-01	1,28	0,18	bacA mutant	WT	PA14_60460	50S ribosomal protein L21
YP_793028.1|gi|116052711|	6	467	9,54E-01	5,72E-01	1,02	0,05	WT	bacA mutant	PA14_60470	octaprenyl-diphosphate synthase
YP_793035.1|gi|116052718|	4	213	3,68E-03	4,75E-02	2,45	0,99	bacA mutant	WT	PA14_60550	ATP-dependent protease
YP_793036.1|gi|116052719|	4	181	7,45E-01	5,27E-01	1,06	0,06	WT	bacA mutant	PA14_60560	hypothetical protein
YP_793038.1|gi|116052721|	3	138	4,67E-01	4,20E-01	1,21	0,10	bacA mutant	WT	PA14_60580	hypothetical protein
YP_793046.1|gi|116052729|	9	623	2,65E-01	3,12E-01	1,10	0,17	WT	bacA mutant	PA14_60700	cytochrome c551 peroxidase
YP_793047.1|gi|116052730|	4	221	2,18E-01	2,76E-01	1,18	0,21	WT	bacA mutant	PA14_60710	glutamate dehydrogenase
YP_793048.1|gi|116052731|	7	490	4,81E-01	4,28E-01	1,27	0,09	bacA mutant	WT	PA14_60730	outer membrane protein
YP_793049.1|gi|116052732|	4	232	1,22E-03	3,32E-02	2,63	1,00	WT	bacA mutant	PA14_60750	protein activator

YP_793051.1|gi|116052734|	2	68	7,81E-01	5,38E-01	1,19	0,06	bacA mutant	WT	PA14_60770	outer membrane protein
YP_793054.1|gi|116052737|	25	1813	1,45E-01	2,18E-01	1,09	0,29	WT	bacA mutant	PA14_60800	ABC transporter ATP-binding protein
YP_793061.1|gi|116052744|	6	274	2,17E-02	9,13E-02	1,30	0,78	WT	bacA mutant	PA14_60890	serine hydroxymethyltransferase
YP_793063.1|gi|116052746|	6	370	5,58E-01	4,57E-01	1,05	0,08	WT	bacA mutant	PA14_60920	hypothetical protein
YP_793065.1|gi|116052748|	3	195	5,56E-01	4,57E-01	1,38	0,08	WT	bacA mutant	PA14_60950	hypothetical protein
YP_793070.1|gi|116052753|	2	89	5,08E-01	4,37E-01	1,24	0,09	bacA mutant	WT	PA14_61010	hypothetical protein
YP_793073.1|gi|116052756|	2	177	7,67E-03	5,89E-02	7,50	0,95	WT	bacA mutant	PA14_61050	large-conductance mechanosensitive channel
YP_793074.1|gi|116052757|	4	170	1,78E-01	2,42E-01	1,11	0,24	bacA mutant	WT	PA14_61060	oxidoreductase
YP_793084.1|gi|116052767|	36	2815	5,52E-02	1,29E-01	1,60	0,53	WT	bacA mutant	PA14_61190	hypothetical protein
YP_793085.1|gi|116052768|	6	344	4,31E-02	1,16E-01	1,74	0,60	WT	bacA mutant	PA14_61200	hypothetical protein
YP_793091.1|gi|116052774|	4	102	6,28E-03	5,40E-02	1,98	0,97	WT	bacA mutant	PA14_61280	epimerase
YP_793092.1|gi|116052775|	6	285	1,18E-01	1,95E-01	1,41	0,33	WT	bacA mutant	PA14_61290	lipoprotein
YP_793101.1|gi|116052784|	10	609	2,84E-01	3,26E-01	1,16	0,16	WT	bacA mutant	PA14_61390	lipoprotein
YP_793102.1|gi|116052785|	11	607	1,35E-01	2,11E-01	1,21	0,30	WT	bacA mutant	PA14_61400	malate:quinone oxidoreductase
YP_793108.1|gi|116052791|	4	203	2,99E-02	1,00E-01	1,50	0,70	WT	bacA mutant	PA14_61470	uracil phosphoribosyltransferase
YP_793117.1|gi|116052800|	3	92	6,04E-01	4,76E-01	1,05	0,07	bacA mutant	WT	PA14_61580	ferrochelatase
YP_793118.1|gi|116052801|	5	283	3,19E-01	3,48E-01	1,06	0,14	bacA mutant	WT	PA14_61590	hypothetical protein
YP_793119.1|gi|116052802|	5	286	3,14E-01	3,44E-01	1,07	0,15	bacA mutant	WT	PA14_61600	hypothetical protein
YP_793123.1|gi|116052806|	8	713	8,04E-01	5,43E-01	1,03	0,05	WT	bacA mutant	PA14_61650	Lipid A 3-O-deacylase
YP_793125.1|gi|116052808|	2	69	9,93E-01	5,83E-01	1,00	0,05	WT	bacA mutant	PA14_61670	molybdopterin biosynthesis protein MoeB
YP_793128.1|gi|116052811|	4	145	9,61E-01	5,73E-01	1,02	0,05	bacA mutant	WT	PA14_61710	glutamyl-tRNA reductase
YP_793129.1|gi|116052812|	9	658	9,83E-01	5,80E-01	1,01	0,05	bacA mutant	WT	PA14_61720	hypothetical protein
YP_793130.1|gi|116052813|	3	245	3,16E-01	3,45E-01	1,17	0,15	WT	bacA mutant	PA14_61740	outer membrane lipoprotein LolB
YP_793132.1|gi|116052815|	16	1258	2,92E-01	3,32E-01	1,08	0,16	WT	bacA mutant	PA14_61770	ribose-phosphate pyrophosphokinase
YP_793133.1|gi|116052816|	11	873	6,54E-01	4,96E-01	1,03	0,07	WT	bacA mutant	PA14_61780	50S ribosomal protein L25
YP_793135.1|gi|116052818|	8	454	8,81E-02	1,64E-01	1,10	0,40	WT	bacA mutant	PA14_61820	GTP-dependent nucleic acid-binding protein EngD

YP_793137.1|gi|116052820|	12	702	7,11E-01	5,18E-01	1,05	0,06	WT	bacA mutant	PA14_61850	TonB-dependent receptor
YP_793151.1|gi|116052834|; YP_791031.1|gi|116050152|	12	674	3,29E-01	3,52E-01	1,20	0,14	bacA mutant	WT	PA14_62020	paraquat-inducible protein B-like protein
YP_793157.1|gi|116052840|	14	1116	1,20E-03	3,32E-02	1,39	1,00	WT	bacA mutant	PA14_62130	ketol-acid reductoisomerase
YP_793158.1|gi|116052841|	3	223	7,38E-01	5,25E-01	1,04	0,06	WT	bacA mutant	PA14_62150	acetolactate synthase 3 regulatory subunit
YP_793159.1|gi|116052842|	8	286	1,63E-01	2,30E-01	1,38	0,26	WT	bacA mutant	PA14_62160	acetolactate synthase 3 catalytic subunit
YP_793162.1|gi|116052845|	4	301	2,98E-01	3,36E-01	1,17	0,15	WT	bacA mutant	PA14_62190	hypothetical protein
YP_793163.1|gi|116052846|	2	68	5,96E-01	4,71E-01	1,17	0,07	bacA mutant	WT	PA14_62200	penicillin-binding protein 1B
YP_793164.1|gi|116052847|	18	1241	7,24E-01	5,19E-01	1,04	0,06	bacA mutant	WT	PA14_62230	hypothetical protein
YP_793171.1|gi|116052854|	7	402	1,27E-02	6,84E-02	1,81	0,89	WT	bacA mutant	PA14_62300	hypothetical protein
YP_793172.1|gi|116052855|	4	160	3,63E-01	3,69E-01	1,09	0,13	WT	bacA mutant	PA14_62330	hemin degrading factor
YP_793173.1|gi|116052856|	17	992	8,35E-02	1,60E-01	1,43	0,42	WT	bacA mutant	PA14_62350	heme/hemoglobin uptake outer membrane receptor PhuR
YP_793178.1|gi|116052861|	5	219	3,44E-01	3,59E-01	1,10	0,13	WT	bacA mutant	PA14_62400	aminotransferase
YP_793180.1|gi|116052863|	2	271	7,83E-01	5,39E-01	1,04	0,06	bacA mutant	WT	PA14_62420	hypothetical protein
YP_793185.1|gi|116052868|	6	303	5,39E-01	4,51E-01	1,09	0,08	bacA mutant	WT	PA14_62480	hypothetical protein
YP_793186.1|gi|116052869|	10	420	1,26E-02	6,84E-02	2,01	0,89	WT	bacA mutant	PA14_62490	suppressor protein DksA
YP_793190.1|gi|116052873|	10	593	6,00E-02	1,35E-01	1,15	0,51	WT	bacA mutant	PA14_62540	two-component response regulator CbrB
YP_793191.1|gi|116052874|	2	105	4,70E-01	4,22E-01	1,10	0,10	WT	bacA mutant	PA14_62560	poly(A) polymerase
YP_793193.1|gi|116052876|	7	480	6,22E-01	4,84E-01	1,07	0,07	bacA mutant	WT	PA14_62580	3-methyl-2-oxobutanoate hydroxymethyltransferase
YP_793194.1|gi|116052877|	6	291	5,47E-01	4,54E-01	1,05	0,08	WT	bacA mutant	PA14_62590	pantoate--beta-alanine ligase
YP_793196.1|gi|116052879|	18	869	1,50E-01	2,21E-01	1,63	0,28	WT	bacA mutant	PA14_62620	glucose-6-phosphate isomerase
YP_793197.1|gi|116052880|	11	527	4,42E-03	4,75E-02	1,77	0,99	WT	bacA mutant	PA14_62630	acetyl-CoA synthetase
YP_793199.1|gi|116052882|	4	190	1,86E-01	2,51E-01	1,88	0,23	WT	bacA mutant	PA14_62650	hypothetical protein
YP_793203.1|gi|116052886|	6	292	1,09E-01	1,87E-01	1,42	0,35	WT	bacA mutant	PA14_62690	hypothetical protein
YP_793204.1|gi|116052887|	38	2679	7,46E-01	5,27E-01	1,04	0,06	WT	bacA mutant	PA14_62710	polynucleotide phosphorylase
YP_793205.1|gi|116052888|	3	223	1,66E-01	2,32E-01	1,25	0,26	bacA mutant	WT	PA14_62720	30S ribosomal protein S15

YP_793206.1|gi|116052889|	3	185	8,99E-01	5,59E-01	1,03	0,05	bacA mutant	WT	PA14_62730	tRNA pseudouridine synthase B
YP_793208.1|gi|116052891|	20	1054	2,25E-01	2,81E-01	1,19	0,20	WT	bacA mutant	PA14_62760	translation initiation factor IF-2
YP_793209.1|gi|116052892|	24	1883	7,79E-01	5,38E-01	1,01	0,06	WT	bacA mutant	PA14_62770	transcription elongation factor NusA
YP_793210.1|gi|116052893|	2	111	3,22E-01	3,49E-01	1,11	0,14	bacA mutant	WT	PA14_62780	hypothetical protein
YP_793211.1|gi|116052894|	3	167	9,07E-01	5,60E-01	1,09	0,05	WT	bacA mutant	PA14_62810	preprotein translocase subunit SecG
YP_793212.1|gi|116052895|	4	188	6,77E-02	1,43E-01	1,15	0,47	WT	bacA mutant	PA14_62830	triosephosphate isomerase
YP_793213.1|gi|116052896|	11	598	4,77E-02	1,20E-01	1,31	0,57	WT	bacA mutant	PA14_62840	phosphoglucosamine mutase
YP_793214.1|gi|116052897|	5	225	9,12E-01	5,61E-01	1,01	0,05	bacA mutant	WT	PA14_62850	dihydropteroate synthase
YP_793215.1|gi|116052898|	14	736	8,46E-01	5,52E-01	1,02	0,05	WT	bacA mutant	PA14_62860	cell division protein FtsH
YP_793219.1|gi|116052902|	7	438	5,77E-02	1,33E-01	1,68	0,52	WT	bacA mutant	PA14_62900	transcription elongation factor GreA
YP_793220.1|gi|116052903|	12	814	7,61E-02	1,51E-01	1,62	0,44	WT	bacA mutant	PA14_62910	carbamoyl phosphate synthase large subunit
YP_793222.1|gi|116052905|	10	594	7,75E-01	5,36E-01	1,05	0,06	WT	bacA mutant	PA14_62930	carbamoyl phosphate synthase small subunit
YP_793223.1|gi|116052906|	12	726	8,75E-01	5,55E-01	1,01	0,05	WT	bacA mutant	PA14_62940	dihydrodipicolinate reductase
YP_793224.1|gi|116052907|	11	487	6,51E-02	1,40E-01	1,23	0,48	WT	bacA mutant	PA14_62960	chaperone protein DnaJ
YP_793225.1|gi|116052908|	44	4048	4,60E-01	4,17E-01	1,08	0,10	WT	bacA mutant	PA14_62970	molecular chaperone DnaK
YP_793226.1|gi|116052909|	10	743	7,00E-02	1,44E-01	1,58	0,46	WT	bacA mutant	PA14_62990	heat shock protein GrpE
YP_793227.1|gi|116052910|	2	75	4,02E-01	3,87E-01	1,09	0,11	WT	bacA mutant	PA14_63010	DNA repair protein RecN
YP_793228.1|gi|116052911|	8	493	4,72E-03	4,75E-02	2,05	0,98	WT	bacA mutant	PA14_63020	ferric uptake regulation protein
YP_793235.1|gi|116052918|	2	63	4,41E-01	4,08E-01	1,07	0,10	bacA mutant	WT	PA14_63090	L-lactate dehydrogenase
YP_793242.1|gi|116052925|	5	212	1,12E-01	1,90E-01	1,19	0,34	bacA mutant	WT	PA14_63170	transcriptional regulator
YP_793249.1|gi|116052932|	10	490	7,53E-03	5,89E-02	1,38	0,95	WT	bacA mutant	PA14_63250	acetyl-CoA acetyltransferase
YP_793250.1|gi|116052933|	17	1010	3,95E-01	3,83E-01	1,05	0,12	WT	bacA mutant	PA14_63270	3-ketoacyl-ACP reductase
YP_793251.1|gi|116052934|	4	137	2,94E-01	3,33E-01	1,21	0,16	WT	bacA mutant	PA14_63280	transcriptional regulator
YP_793252.1|gi|116052935|	2	70	6,42E-01	4,92E-01	1,05	0,07	WT	bacA mutant	PA14_63290	hypothetical protein
YP_793256.1|gi|116052939|	2	70	1,68E-01	2,33E-01	1,51	0,26	WT	bacA mutant	PA14_63330	glycerolphosphodiesterase
YP_793257.1|gi|116052940|	6	272	4,60E-02	1,19E-01	1,57	0,58	WT	bacA mutant	PA14_63340	lipoprotein

YP_793272.1|gi|116052955|	2	100	3,75E-01	3,73E-01	1,21	0,12	WT	bacA mutant	PA14_63530	selenocysteine-specific elongation factor
YP_793273.1|gi|116052956|	10	512	6,86E-02	1,44E-01	1,31	0,47	bacA mutant	WT	PA14_63540	selenocysteine synthase
YP_793277.1|gi|116054418|	3	153	7,48E-01	5,28E-01	1,04	0,06	bacA mutant	WT	PA14_63605	formate dehydrogenase-O, major subunit
YP_793293.1|gi|116052975|	2	101	6,39E-01	4,90E-01	1,11	0,07	WT	bacA mutant	PA14_63830	N-hydroxyarylamine O-acetyltransferase
YP_793295.1|gi|116052977|	4	241	3,66E-02	1,09E-01	1,46	0,64	WT	bacA mutant	PA14_63850	dihydrolipoamide dehydrogenase
YP_793305.1|gi|116052987|	5	214	4,40E-01	4,08E-01	1,18	0,10	WT	bacA mutant	PA14_63990	arginine decarboxylase
YP_793308.1|gi|116052990|	14	1126	9,93E-01	5,83E-01	1,03	0,05	WT	bacA mutant	PA14_64030	hypothetical protein
YP_793309.1|gi|116052991|	3	142	1,64E-01	2,31E-01	1,32	0,26	WT	bacA mutant	PA14_64050	two-component response regulator
YP_793312.1|gi|116052994|	4	462	1,52E-01	2,22E-01	1,33	0,27	bacA mutant	WT	PA14_64090	3-dehydroquinate dehydratase
YP_793313.1|gi|116052995|	5	472	6,93E-02	1,44E-01	1,70	0,47	WT	bacA mutant	PA14_64100	acetyl-CoA carboxylase biotin carboxyl carrier protein subunit
YP_793314.1|gi|116052996|	16	929	1,11E-01	1,90E-01	1,18	0,35	WT	bacA mutant	PA14_64110	acetyl-CoA carboxylase biotin carboxylase subunit
YP_793316.1|gi|116052998|	3	131	2,61E-01	3,09E-01	1,21	0,17	bacA mutant	WT	PA14_64140	50S ribosomal protein L11 methyltransferase
YP_793317.1|gi|116052999|	6	283	2,94E-01	3,33E-01	1,10	0,16	bacA mutant	WT	PA14_64170	hypothetical protein
YP_793320.1|gi|116053002|	10	458	3,37E-01	3,55E-01	1,11	0,14	bacA mutant	WT	PA14_64200	bifunctional phosphoribosylaminoimidazolecarboxamide formyltransferase/IMP cyclohydrolase
YP_793321.1|gi|116053003|	8	526	8,93E-02	1,66E-01	1,21	0,40	WT	bacA mutant	PA14_64220	phosphoribosylamine--glycine ligase
YP_793335.1|gi|116053016|	5	242	1,66E-01	2,32E-01	1,16	0,26	bacA mutant	WT	PA14_64390	urease subunit alpha
YP_793340.1|gi|116053021|	4	260	3,96E-01	3,83E-01	1,09	0,12	WT	bacA mutant	PA14_64440	hypothetical protein
YP_793342.1|gi|116053023|	4	120	1,31E-01	2,07E-01	2,02	0,31	WT	bacA mutant	PA14_64460	hypothetical protein
YP_793344.1|gi|116053025|	9	688	4,57E-02	1,19E-01	1,34	0,58	WT	bacA mutant	PA14_64480	DNA-binding transcriptional activator OsmE
YP_793345.1|gi|116053026|	2	99	4,69E-01	4,22E-01	1,21	0,10	WT	bacA mutant	PA14_64490	hypothetical protein
YP_793348.1|gi|116053029|	10	595	6,35E-02	1,38E-01	1,13	0,49	bacA mutant	WT	PA14_64520	bacterioferritin
YP_793375.1|gi|116053056|	5	195	2,37E-03	4,16E-02	1,37	1,00	WT	bacA mutant	PA14_64840	short-chain dehydrogenase
YP_793381.1|gi|116053062|	2	118	1,13E-02	6,67E-02	2,22	0,91	WT	bacA mutant	PA14_64900	ABC transporter substrate-binding protein
YP_793383.1|gi|116053064|	6	262	4,70E-01	4,22E-01	1,50	0,10	WT	bacA mutant	PA14_64920	methyl-accepting chemotaxis protein
YP_793384.1|gi|116053065|	5	481	5,89E-01	4,69E-01	1,06	0,07	bacA mutant	WT	PA14_64930	hypothetical protein
YP_793388.1|gi|116053069|	8	539	3,38E-02	1,06E-01	1,30	0,66	WT	bacA mutant	PA14_64980	NAD synthetase

YP_793390.1|gi|116053071|	8	266	4,21E-03	4,75E-02	3,05	0,99	WT	bacA mutant	PA14_65000	azurin
YP_793398.1|gi|116053079|	3	143	8,61E-01	5,53E-01	1,03	0,05	WT	bacA mutant	PA14_65110	biosynthetic alanine racemase
YP_793399.1|gi|116053080|	3	115	5,71E-02	1,32E-01	1,33	0,52	WT	bacA mutant	PA14_65130	replicative DNA helicase
YP_793400.1|gi|116053081|	8	583	2,95E-01	3,34E-01	1,10	0,16	WT	bacA mutant	PA14_65150	50S ribosomal protein L9
YP_793403.1|gi|116053084|	10	820	3,52E-01	3,64E-01	1,18	0,13	WT	bacA mutant	PA14_65180	30S ribosomal protein S6
YP_793404.1|gi|116053085|	3	125	1,01E-01	1,80E-01	1,18	0,37	WT	bacA mutant	PA14_65190	TrmH family RNA methyltransferase , group 3
YP_793405.1|gi|116053086|	7	276	2,62E-01	3,09E-01	1,36	0,17	WT	bacA mutant	PA14_65200	exoribonuclease RNase R
YP_793406.1|gi|116053087|	20	1583	8,38E-01	5,51E-01	1,01	0,05	bacA mutant	WT	PA14_65230	adenylosuccinate synthetase
YP_793407.1|gi|116053088|	10	604	2,23E-01	2,81E-01	1,12	0,20	bacA mutant	WT	PA14_65250	ATP phosphoribosyltransferase
YP_793409.1|gi|116053090|	15	853	9,90E-01	5,83E-01	1,05	0,05	WT	bacA mutant	PA14_65270	protease subunit HflC
YP_793410.1|gi|116053091|	18	1568	6,44E-01	4,92E-01	1,06	0,07	WT	bacA mutant	PA14_65280	protease subunit HflK
YP_793411.1|gi|116053092|	8	411	7,83E-02	1,54E-01	1,28	0,43	bacA mutant	WT	PA14_65300	GTP-binding protein
YP_793412.1|gi|116053093|	7	388	4,99E-01	4,34E-01	1,11	0,09	bacA mutant	WT	PA14_65310	RNA-binding protein Hfq
YP_793414.1|gi|116053095|	6	207	1,94E-01	2,57E-01	1,16	0,23	WT	bacA mutant	PA14_65350	DNA mismatch repair protein
YP_793417.1|gi|116053098|	3	197	6,50E-02	1,40E-01	1,22	0,48	bacA mutant	WT	PA14_65390	hypothetical protein
YP_793419.1|gi|116053100|	3	118	5,83E-02	1,33E-01	1,69	0,51	WT	bacA mutant	PA14_65410	oligoribonuclease
YP_793421.1|gi|116053102|	4	226	2,16E-01	2,76E-01	1,14	0,21	WT	bacA mutant	PA14_65430	flagellar motor protein MotB
YP_793423.1|gi|116053104|	4	177	5,28E-01	4,47E-01	1,12	0,08	bacA mutant	WT	PA14_65470	hypothetical protein
YP_793424.1|gi|116053105|	3	186	4,11E-01	3,92E-01	1,28	0,11	bacA mutant	WT	PA14_65480	thiosulfate sulfurtransferase
YP_793425.1|gi|116053106|	5	209	6,36E-01	4,88E-01	1,14	0,07	WT	bacA mutant	PA14_65500	phosphatidylserine decarboxylase
YP_793426.1|gi|116053107|	3	150	6,73E-01	5,04E-01	1,11	0,06	bacA mutant	WT	PA14_65520	hypothetical protein
YP_793427.1|gi|116053108|	6	390	3,90E-01	3,81E-01	1,23	0,12	WT	bacA mutant	PA14_65540	hypothetical protein
YP_793428.1|gi|116053109|	4	121	6,21E-01	4,84E-01	1,11	0,07	WT	bacA mutant	PA14_65560	phosphoserine phosphatase
YP_793429.1|gi|116053110|	7	313	6,94E-01	5,11E-01	1,27	0,06	WT	bacA mutant	PA14_65570	hypothetical protein
YP_793431.1|gi|116053112|	3	182	4,78E-01	4,26E-01	1,11	0,09	bacA mutant	WT	PA14_65590	hypothetical protein
YP_793432.1|gi|116054424|	8	416	3,37E-02	1,06E-01	1,44	0,67	bacA mutant	WT	PA14_65605	DNA topoisomerase IV subunit A

YP_793434.1|gi|116053114|	2	42	9,35E-01	5,69E-01	1,01	0,05	WT	bacA mutant	PA14_65640	hypothetical protein
YP_793435.1|gi|116053115|	8	417	2,47E-01	2,99E-01	1,20	0,18	WT	bacA mutant	PA14_65660	DNA topoisomerase IV subunit B
YP_793439.1|gi|116053119|	4	196	4,17E-01	3,96E-01	1,17	0,11	WT	bacA mutant	PA14_65710	adenosine diphosphate sugar pyrophosphatase
YP_793440.1|gi|116053120|	3	80	3,36E-01	3,55E-01	1,11	0,14	WT	bacA mutant	PA14_65720	lipoprotein
YP_793442.1|gi|116053122|	26	2514	4,01E-01	3,87E-01	1,33	0,11	bacA mutant	WT	PA14_65750	outer membrane efflux protein
YP_793456.1|gi|116053135|	2	84	2,95E-01	3,34E-01	1,48	0,15	bacA mutant	WT	PA14_65950	transcriptional regulator
YP_793457.1|gi|116053136|	2	78	4,36E-01	4,07E-01	1,26	0,10	WT	bacA mutant	PA14_65960	3-deoxy-D-manno-octulosonic-acid transferase
YP_793465.1|gi|116053144|	8	371	2,66E-01	3,12E-01	1,08	0,17	WT	bacA mutant	PA14_66060	bifunctional heptose 7-phosphate kinase/heptose 1-phosphate adenyltransferase
YP_793466.1|gi|116053145|	2	155	8,74E-01	5,55E-01	1,04	0,05	WT	bacA mutant	PA14_66080	transport protein MsbA
YP_793467.1|gi|116053146|	2	60	2,83E-01	3,26E-01	1,42	0,16	bacA mutant	WT	PA14_66090	hypothetical protein
YP_793469.1|gi|116053148|	2	72	4,55E-02	1,19E-01	1,82	0,58	WT	bacA mutant	PA14_66110	glycosyl transferase family protein
YP_793470.1|gi|116053149|	5	171	5,89E-01	4,69E-01	1,10	0,07	bacA mutant	WT	PA14_66120	hypothetical protein
YP_793472.1|gi|116053151|	3	134	2,92E-01	3,32E-01	1,22	0,16	bacA mutant	WT	PA14_66150	hypothetical protein
YP_793473.1|gi|116053152|	8	463	1,02E-01	1,81E-01	1,32	0,37	bacA mutant	WT	PA14_66160	glycosyl transferase family protein
YP_793474.1|gi|116053153|	19	1124	4,98E-02	1,21E-01	1,41	0,56	WT	bacA mutant	PA14_66170	carbamoyl transferase
YP_793475.1|gi|116053154|	5	285	1,83E-01	2,47E-01	1,27	0,24	bacA mutant	WT	PA14_66190	hypothetical protein
YP_793477.1|gi|116053156|	3	208	6,72E-01	5,04E-01	1,07	0,06	WT	bacA mutant	PA14_66210	hypothetical protein
YP_793478.1|gi|116053157|	2	78	8,30E-01	5,49E-01	1,07	0,05	bacA mutant	WT	PA14_66220	lipopolysaccharide kinase WaaP
YP_793479.1|gi|116053158|	8	405	3,86E-01	3,80E-01	1,17	0,12	bacA mutant	WT	PA14_66230	UDP-glucose:(heptosyl) LPS alpha 1,3-glucosyltransferase WaaG
YP_793480.1|gi|116053159|	5	328	1,44E-01	2,17E-01	1,31	0,29	bacA mutant	WT	PA14_66240	lipopolysaccharide heptosyltransferase I
YP_793481.1|gi|116053160|	4	295	6,30E-02	1,38E-01	1,27	0,49	bacA mutant	WT	PA14_66250	heptosyltransferase II
YP_793482.1|gi|116053161|	17	1228	1,05E-01	1,84E-01	1,18	0,36	WT	bacA mutant	PA14_66260	branched-chain amino acid aminotransferase
YP_793484.1|gi|116053163|	21	998	9,08E-01	5,60E-01	1,02	0,05	bacA mutant	WT	PA14_66290	pyruvate dehydrogenase subunit E1
YP_793485.1|gi|116053164|	27	1725	1,47E-03	3,69E-02	1,31	1,00	WT	bacA mutant	PA14_66310	dihydrolipoamide acetyltransferase
YP_793487.1|gi|116053166|	4	196	1,09E-02	6,67E-02	1,78	0,91	WT	bacA mutant	PA14_66330	peptide methionine sulfoxide reductase
YP_793488.1|gi|116053167|	2	93	5,67E-02	1,32E-01	1,23	0,52	bacA mutant	WT	PA14_66340	hypothetical protein

YP_793491.1|gi|116053170|	4	132	6,49E-01	4,93E-01	1,00	0,07	WT	bacA mutant	PA14_66400	potassium efflux protein KefA
YP_793492.1|gi|116053171|	2	75	4,28E-01	4,04E-01	1,16	0,11	WT	bacA mutant	PA14_66410	hypothetical protein
YP_793494.1|gi|116053173|	9	495	4,90E-02	1,20E-01	1,30	0,56	WT	bacA mutant	PA14_66440	O-acetylhomoserine aminocarboxypropyltransferase
YP_793496.1|gi|116053175|	13	900	7,03E-03	5,85E-02	1,15	0,96	bacA mutant	WT	PA14_66460	hypothetical protein
YP_793497.1|gi|116053176|	3	247	6,10E-03	5,36E-02	1,46	0,97	WT	bacA mutant	PA14_66480	hypothetical protein
YP_793503.1|gi|116053182|	5	362	2,30E-02	9,26E-02	1,53	0,76	WT	bacA mutant	PA14_66550	uroporphyrinogen decarboxylase
YP_793504.1|gi|116053183|	5	297	6,93E-01	5,11E-01	1,07	0,06	WT	bacA mutant	PA14_66560	glutamate synthase subunit beta
YP_793506.1|gi|116053185|	5	280	8,66E-01	5,54E-01	1,01	0,05	bacA mutant	WT	PA14_66580	hypothetical protein
YP_793507.1|gi|116053186|	5	427	1,42E-01	2,17E-01	1,36	0,29	bacA mutant	WT	PA14_66600	3-dehydroquinate synthase
YP_793509.1|gi|116053188|	31	2018	3,75E-01	3,73E-01	1,45	0,12	bacA mutant	WT	PA14_66620	type 4 fimbrial biogenesis outer membrane protein PilQ precursor
YP_793511.1|gi|116053190|	3	223	7,67E-01	5,34E-01	1,04	0,06	WT	bacA mutant	PA14_66640	type 4 fimbrial biogenesis protein PilO
YP_793512.1|gi|116053191|	5	193	8,33E-01	5,50E-01	1,12	0,05	WT	bacA mutant	PA14_66650	type 4 fimbrial biogenesis protein PilN
YP_793513.1|gi|116053192|	4	237	3,12E-01	3,43E-01	1,07	0,15	WT	bacA mutant	PA14_66660	type 4 fimbrial biogenesis protein PilM
YP_793514.1|gi|116053193|	2	123	9,99E-01	5,85E-01	1,08	0,05	WT	bacA mutant	PA14_66670	penicillin-binding protein 1A
YP_793515.1|gi|116053194|	24	1497	1,52E-02	7,49E-02	1,33	0,86	WT	bacA mutant	PA14_66680	malic enzyme
YP_793520.1|gi|116053199|	20	1122	7,67E-01	5,34E-01	1,05	0,06	WT	bacA mutant	PA14_66750	arginyl-tRNA synthetase
YP_793522.1|gi|116053201|	6	321	1,76E-01	2,41E-01	1,16	0,24	bacA mutant	WT	PA14_66770	ATP-dependent protease peptidase subunit
YP_793523.1|gi|116053202|	14	711	2,11E-01	2,72E-01	1,27	0,21	bacA mutant	WT	PA14_66790	ATP-dependent protease ATP-binding subunit HslU
YP_793525.1|gi|116053204|	7	345	8,60E-02	1,62E-01	1,50	0,41	WT	bacA mutant	PA14_66820	poly(3-hydroxyalkanoic acid) synthase 1
YP_793527.1|gi|116053206|	11	609	6,35E-01	4,88E-01	1,10	0,07	WT	bacA mutant	PA14_66840	poly(3-hydroxyalkanoic acid) synthase 2
YP_793528.1|gi|116053207|	2	105	8,61E-01	5,53E-01	1,18	0,05	WT	bacA mutant	PA14_66850	TetR family transcriptional regulator
YP_793529.1|gi|116054455|	4	207	5,06E-01	4,36E-01	1,20	0,09	WT	bacA mutant	PA14_66875	polyhydroxyalkanoate synthesis protein PhaF
YP_793530.1|gi|116053208|	7	363	1,23E-01	1,99E-01	1,44	0,32	WT	bacA mutant	PA14_66880	hypothetical protein
YP_793532.1|gi|116053210|	11	530	2,04E-02	8,81E-02	1,55	0,79	WT	bacA mutant	PA14_66900	ubiquinone/menaquinone biosynthesis methyltransferase
YP_793533.1|gi|116053211|	2	81	1,47E-01	2,19E-01	1,38	0,28	bacA mutant	WT	PA14_66910	hypothetical protein
YP_793534.1|gi|116053212|	5	160	8,43E-01	5,52E-01	1,14	0,05	WT	bacA mutant	PA14_66920	ubiquinone biosynthesis protein UbiB

YP_793537.1|gi|116053215|	2	73	5,13E-01	4,41E-01	1,14	0,09	WT	bacA mutant	PA14_66960	twin arginine translocase A
YP_793545.1|gi|116053223|	4	253	1,21E-01	1,98E-01	1,73	0,33	WT	bacA mutant	PA14_67050	ABC transporter substrate-binding protein
YP_793547.1|gi|116053224|	15	899	9,05E-01	5,59E-01	1,01	0,05	WT	bacA mutant	PA14_67090	glucan biosynthesis protein G
YP_793549.1|gi|116053226|	7	324	9,37E-03	6,32E-02	1,19	0,93	WT	bacA mutant	PA14_67110	prolyl aminopeptidase
YP_793561.1|gi|116053238|	6	292	4,68E-02	1,20E-01	1,26	0,58	WT	bacA mutant	PA14_67240	N-formylglutamate amidohydrolase
YP_793562.1|gi|116053239|	5	222	1,68E-01	2,33E-01	1,19	0,26	WT	bacA mutant	PA14_67250	imidazolonepropionase
YP_793564.1|gi|116053241|	2	177	6,42E-01	4,92E-01	1,07	0,07	bacA mutant	WT	PA14_67270	ABC transporter ATP-binding protein
YP_793568.1|gi|116053245|	8	428	3,41E-02	1,06E-01	1,24	0,66	bacA mutant	WT	PA14_67320	histidine ammonia-lyase
YP_793570.1|gi|116053247|	20	1606	9,26E-01	5,65E-01	1,02	0,05	WT	bacA mutant	PA14_67350	urocanate hydratase
YP_793575.1|gi|116053252|	3	158	8,20E-01	5,47E-01	1,02	0,05	WT	bacA mutant	PA14_67420	histidine utilization genes repressor protein
YP_793576.1|gi|116053253|	9	572	5,25E-03	4,97E-02	1,60	0,98	bacA mutant	WT	PA14_67440	N-formimino-L-glutamate deiminase
YP_793577.1|gi|116053254|	5	200	2,61E-01	3,09E-01	1,20	0,17	WT	bacA mutant	PA14_67450	outer membrane lipoprotein Blc
YP_793579.1|gi|116053256|	2	58	7,06E-01	5,16E-01	1,06	0,06	WT	bacA mutant	PA14_67470	hypothetical protein
YP_793580.1|gi|116053257|	10	608	7,38E-01	5,25E-01	1,05	0,06	bacA mutant	WT	PA14_67490	fructose-1,6-bisphosphatase
YP_793581.1|gi|116053258|	5	182	9,46E-02	1,73E-01	1,18	0,39	WT	bacA mutant	PA14_67500	lactoylglutathione lyase
YP_793582.1|gi|116053259|	21	1670	9,47E-01	5,72E-01	1,01	0,05	bacA mutant	WT	PA14_67510	esterase EstA
YP_793583.1|gi|116053260|	6	276	4,39E-01	4,08E-01	1,22	0,10	bacA mutant	WT	PA14_67520	hypothetical protein
YP_793585.1|gi|116053262|	4	192	4,22E-01	3,99E-01	1,10	0,11	WT	bacA mutant	PA14_67540	hypothetical protein
YP_793587.1|gi|116053264|	5	310	1,00E-01	1,80E-01	1,33	0,37	bacA mutant	WT	PA14_67560	TypA
YP_793589.1|gi|116053266|	20	1532	2,50E-03	4,19E-02	1,45	1,00	WT	bacA mutant	PA14_67600	glutamine synthetase
YP_793595.1|gi|116053272|	3	85	1,25E-01	2,01E-01	1,27	0,32	WT	bacA mutant	PA14_67680	two-component response regulator NtrC
YP_793598.1|gi|116053275|	6	441	3,27E-01	3,52E-01	1,10	0,14	WT	bacA mutant	PA14_67720	preprotein translocase subunit SecB
YP_793601.1|gi|116053278|	13	692	2,73E-01	3,18E-01	1,08	0,17	bacA mutant	WT	PA14_67770	phosphoglyceromutase
YP_793603.1|gi|116053280|	3	100	6,15E-02	1,36E-01	1,70	0,50	bacA mutant	WT	PA14_67790	membrane-bound metallopeptidase
YP_793604.1|gi|116053281|	13	577	3,50E-01	3,63E-01	1,05	0,13	WT	bacA mutant	PA14_67810	carboxyl-terminal protease
YP_793611.1|gi|116053288|	3	86	4,29E-01	4,04E-01	1,05	0,11	WT	bacA mutant	PA14_67890	1-(5-phosphoribosyl)-5-[(5- phosphoribosylamino)methylideneamino] imidazole-4-carboxamide isomerase

YP_793613.1|gi|116053290|	3	154	7,26E-03	5,85E-02	1,31	0,96	WT	bacA mutant	PA14_67920	imidazole glycerol phosphate synthase subunit HisH
YP_793614.1|gi|116053291|	4	166	1,88E-03	3,93E-02	2,04	1,00	WT	bacA mutant	PA14_67930	imidazoleglycerol-phosphate dehydratase
YP_793618.1|gi|116054460|	7	312	7,44E-01	5,27E-01	1,05	0,06	bacA mutant	WT	PA14_67975	hypothetical protein
YP_793620.1|gi|116053296|	3	105	7,23E-02	1,47E-01	1,76	0,46	WT	bacA mutant	PA14_68000	hypothetical protein
YP_793621.1|gi|116053297|	5	221	1,90E-01	2,53E-01	1,25	0,23	bacA mutant	WT	PA14_68040	short-chain dehydrogenase
YP_793623.1|gi|116053299|	3	104	1,03E-01	1,83E-01	1,25	0,36	WT	bacA mutant	PA14_68060	ABC transporter ATP-binding protein
YP_793624.1|gi|116053300|	6	248	6,08E-03	5,36E-02	2,36	0,97	WT	bacA mutant	PA14_68070	periplasmic binding protein
YP_793632.1|gi|116053308|	16	1282	4,30E-01	4,04E-01	1,07	0,11	WT	bacA mutant	PA14_68170	dTDP-D-glucose 4,6-dehydratase
YP_793633.1|gi|116053309|	12	671	1,98E-01	2,60E-01	1,15	0,22	bacA mutant	WT	PA14_68190	dTDP-4-dehydrorhamnose reductase
YP_793634.1|gi|116053310|	8	514	1,49E-01	2,19E-01	1,07	0,28	WT	bacA mutant	PA14_68200	glucose-1-phosphate thymidylyltransferase
YP_793635.1|gi|116053311|	6	344	3,58E-01	3,66E-01	1,10	0,13	bacA mutant	WT	PA14_68210	dTDP-4-dehydrorhamnose 3,5-epimerase
YP_793637.1|gi|116053313|	6	344	6,09E-02	1,36E-01	1,03	0,50	WT	bacA mutant	PA14_68250	two-component response regulator
YP_793638.1|gi|116053314|;<br>YP_792392.1|gi|116048808|	4	133	4,54E-03	4,75E-02	1,86	0,99	WT	bacA mutant	PA14_68260	c4-dicarboxylate-binding protein
YP_793641.1|gi|116053317|	3	182	7,51E-01	5,28E-01	1,01	0,06	bacA mutant	WT	PA14_68300	arginine/ornithine antiporter
YP_793642.1|gi|116053318|	27	2394	4,71E-02	1,20E-01	1,15	0,57	bacA mutant	WT	PA14_68330	arginine deiminase
YP_793643.1|gi|116053319|	28	1631	7,97E-02	1,56E-01	1,15	0,43	WT	bacA mutant	PA14_68340	ornithine carbamoyltransferase
YP_793644.1|gi|116053320|	12	748	3,93E-02	1,13E-01	1,12	0,62	WT	bacA mutant	PA14_68350	carbamate kinase
YP_793645.1|gi|116053321|	17	1344	5,56E-01	4,57E-01	1,06	0,08	bacA mutant	WT	PA14_68360	beta-ketoacyl synthase
YP_793646.1|gi|116053322|	3	93	3,67E-01	3,71E-01	1,18	0,12	bacA mutant	WT	PA14_68370	3'(2'),5'-bisphosphate nucleotidase
YP_793647.1|gi|116053323|	3	149	1,01E-01	1,81E-01	1,24	0,37	WT	bacA mutant	PA14_68380	ADP-ribose diphosphatase NudE
YP_793649.1|gi|116053325|	15	1360	3,39E-04	2,46E-02	2,30	1,00	WT	bacA mutant	PA14_68400	LysM domain/BON superfamily protein
YP_793658.1|gi|116053334|	4	134	7,07E-02	1,45E-01	1,35	0,46	WT	bacA mutant	PA14_68500	iron-containing alcohol dehydrogenase
YP_793660.1|gi|116053336|	6	405	2,10E-01	2,71E-01	1,18	0,21	WT	bacA mutant	PA14_68530	3-hydroxyacyl-CoA dehydrogenase
YP_793664.1|gi|116053340|	14	912	2,80E-02	9,88E-02	1,13	0,71	bacA mutant	WT	PA14_68580	phosphoenolpyruvate carboxykinase
YP_793665.1|gi|116053341|	8	317	2,32E-01	2,87E-01	1,21	0,19	bacA mutant	WT	PA14_68610	Hsp33-like chaperonin

YP_793667.1|gi|116053343|	2	46	3,42E-02	1,06E-01	1,42	0,66	bacA mutant	WT	PA14_68630	heat shock protein
YP_793668.1|gi|116053344|	2	122	2,63E-02	9,65E-02	1,22	0,73	bacA mutant	WT	PA14_68640	hypothetical protein
YP_793669.1|gi|116053345|	2	109	9,21E-02	1,70E-01	1,27	0,39	bacA mutant	WT	PA14_68660	ribosomal protein S6 modification protein
YP_793670.1|gi|116053346|	2	71	9,33E-02	1,72E-01	1,60	0,39	WT	bacA mutant	PA14_68670	carboxypeptidase
YP_793671.1|gi|116053347|	4	211	7,90E-01	5,39E-01	1,08	0,06	WT	bacA mutant	PA14_68680	two-component sensor EnvZ
YP_793672.1|gi|116053348|	9	434	2,29E-02	9,26E-02	1,35	0,77	WT	bacA mutant	PA14_68700	osmolarity response regulator
YP_793673.1|gi|116053349|	2	97	4,73E-02	1,20E-01	1,82	0,57	bacA mutant	WT	PA14_68710	hypothetical protein
YP_793675.1|gi|116053351|	8	337	4,28E-01	4,04E-01	1,16	0,11	bacA mutant	WT	PA14_68730	glutamate--cysteine ligase
YP_793676.1|gi|116053352|	2	103	1,01E-01	1,80E-01	1,64	0,37	bacA mutant	WT	PA14_68740	N-acetylglutamate synthase
YP_793678.1|gi|116053354|	2	71	2,54E-01	3,04E-01	1,13	0,18	WT	bacA mutant	PA14_68770	acetylornithine deacetylase
YP_793680.1|gi|116053356|	5	350	1,44E-01	2,17E-01	1,21	0,29	WT	bacA mutant	PA14_68800	hypothetical protein
YP_793681.1|gi|116053357|	5	276	2,64E-01	3,11E-01	1,30	0,17	bacA mutant	WT	PA14_68810	hypothetical protein
YP_793682.1|gi|116053358|	10	567	8,29E-01	5,49E-01	1,02	0,05	WT	bacA mutant	PA14_68820	secretion pathway ATPase
YP_793685.1|gi|116053361|	3	151	2,90E-02	9,95E-02	1,73	0,71	WT	bacA mutant	PA14_68850	glycine dehydrogenase
YP_793686.1|gi|116053362|	3	140	5,83E-02	1,33E-01	1,91	0,51	WT	bacA mutant	PA14_68860	glycine cleavage system protein H
YP_793687.1|gi|116053363|	5	190	1,55E-01	2,25E-01	1,13	0,27	WT	bacA mutant	PA14_68870	glycine cleavage system aminomethyltransferase T
YP_793689.1|gi|116053365|	9	455	4,03E-02	1,14E-01	1,46	0,62	WT	bacA mutant	PA14_68900	iron ABC transporter substrate-binding protein
YP_793692.1|gi|116053368|	5	272	1,10E-02	6,67E-02	1,57	0,91	WT	bacA mutant	PA14_68940	hypothetical protein
YP_793693.1|gi|116054439|	5	200	5,91E-01	4,69E-01	1,07	0,07	bacA mutant	WT	PA14_68955	2-octaprenyl-3-methyl-6-methoxy-1,4-benzoquinol hydroxylase
YP_793695.1|gi|116053370|	5	189	5,39E-02	1,28E-01	1,10	0,54	WT	bacA mutant	PA14_68980	2-octaprenyl-6-methoxyphenyl hydroxylase
YP_793696.1|gi|116053371|	13	719	4,34E-01	4,06E-01	1,12	0,10	WT	bacA mutant	PA14_69000	aminopeptidase
YP_793697.1|gi|116053372|	3	167	8,63E-01	5,53E-01	1,03	0,05	WT	bacA mutant	PA14_69010	hypothetical protein
YP_793701.1|gi|116053376|	3	117	6,99E-02	1,44E-01	1,81	0,46	WT	bacA mutant	PA14_69050	hypothetical protein
YP_793703.1|gi|116053378|	8	362	9,66E-01	5,75E-01	1,18	0,05	WT	bacA mutant	PA14_69070	ABC transporter ATP-binding protein/permease
YP_793704.1|gi|116053379|	16	877	9,44E-01	5,71E-01	1,00	0,05	WT	bacA mutant	PA14_69090	hypothetical protein
YP_793706.1|gi|116053381|	3	143	8,59E-01	5,53E-01	1,02	0,05	bacA mutant	WT	PA14_69110	oxidoreductase

YP_793709.1|gi|116053384|	5	285	1,59E-01	2,26E-01	1,21	0,27	WT	bacA mutant	PA14_69150	hypothetical protein
YP_793711.1|gi|116053386|	18	938	8,83E-01	5,56E-01	1,02	0,05	WT	bacA mutant	PA14_69190	transcription termination factor Rho
YP_793712.1|gi|116053387|	5	243	2,32E-02	9,26E-02	1,51	0,76	WT	bacA mutant	PA14_69200	thioredoxin
YP_793713.1|gi|116053388|	11	550	7,18E-01	5,18E-01	1,04	0,06	bacA mutant	WT	PA14_69220	exopolyphosphatase
YP_793714.1|gi|116053389|	17	844	8,10E-01	5,44E-01	1,11	0,05	WT	bacA mutant	PA14_69230	polyphosphate kinase
YP_793715.1|gi|116053390|	11	800	2,58E-02	9,63E-02	1,39	0,74	WT	bacA mutant	PA14_69240	delta-aminolevulinic acid dehydratase
YP_793717.1|gi|116053392|	6	484	4,90E-03	4,75E-02	2,00	0,98	WT	bacA mutant	PA14_69260	isoprenoid biosynthesis protein with amidotransferase-like domain
YP_793723.1|gi|116053398|	2	125	7,73E-01	5,36E-01	1,17	0,06	WT	bacA mutant	PA14_69330	hypothetical protein
YP_793724.1|gi|116053399|	2	99	1,62E-01	2,29E-01	1,22	0,26	bacA mutant	WT	PA14_69340	ABC transporter ATP-binding protein
YP_793726.1|gi|116053401|	3	221	9,25E-01	5,65E-01	1,09	0,05	WT	bacA mutant	PA14_69370	alginate regulatory protein AlgP
YP_793730.1|gi|116053405|	13	729	6,79E-01	5,06E-01	1,05	0,06	bacA mutant	WT	PA14_69420	enzyme of heme biosynthesis
YP_793731.1|gi|116053406|	14	978	9,73E-01	5,77E-01	1,03	0,05	WT	bacA mutant	PA14_69430	hypothetical protein
YP_793733.1|gi|116053408|	4	195	3,39E-03	4,75E-02	1,53	0,99	WT	bacA mutant	PA14_69450	porphobilinogen deaminase
YP_793734.1|gi|116053409|	11	582	8,68E-02	1,63E-01	1,21	0,41	WT	bacA mutant	PA14_69470	alginate biosynthesis regulatory protein AlgR
YP_793736.1|gi|116053411|	5	201	5,43E-01	4,52E-01	1,20	0,08	WT	bacA mutant	PA14_69500	argininosuccinate lyase
YP_793748.1|gi|116053423|	5	487	6,57E-02	1,40E-01	1,65	0,48	WT	bacA mutant	PA14_69630	nucleoside diphosphate kinase regulator
YP_793751.1|gi|116053426|	12	861	7,15E-01	5,18E-01	1,02	0,06	WT	bacA mutant	PA14_69670	diaminopimelate decarboxylase
YP_793752.1|gi|116053427|	2	126	8,41E-02	1,60E-01	1,28	0,42	WT	bacA mutant	PA14_69690	diaminopimelate epimerase
YP_793762.1|gi|116053436|	4	319	3,50E-01	3,63E-01	1,32	0,13	WT	bacA mutant	PA14_69810	nitrogen regulatory protein P-II 2
YP_793765.1|gi|116053439|;<br>YP_793849.1|gi|116053522|	5	220	4,85E-01	4,29E-01	1,26	0,09	bacA mutant	WT	PA14_69850	choline transporter
YP_793773.1|gi|116053446|	6	289	3,15E-02	1,03E-01	1,36	0,68	WT	bacA mutant	PA14_69950	hypothetical protein
YP_793774.1|gi|116053447|	3	145	2,00E-01	2,61E-01	1,53	0,22	WT	bacA mutant	PA14_69970	cytochrome c5
YP_793776.1|gi|116053449|	3	152	9,17E-01	5,63E-01	1,01	0,05	WT	bacA mutant	PA14_69990	alanine racemase
YP_793777.1|gi|116053450|	4	211	9,09E-02	1,69E-01	1,38	0,40	WT	bacA mutant	PA14_70010	hypothetical protein
YP_793778.1|gi|116053451|	13	652	1,06E-01	1,85E-01	1,21	0,36	bacA mutant	WT	PA14_70040	D-amino acid dehydrogenase small subunit

YP_793782.1|gi|116053455|	4	174	2,37E-01	2,91E-01	1,35	0,19	WT	bacA mutant	PA14_70080	leucine-responsive regulatory protein
YP_793786.1|gi|116053459|	24	2131	1,06E-01	1,85E-01	1,21	0,36	WT	bacA mutant	PA14_70140	aldehyde dehydrogenase
YP_793787.1|gi|116053460|	10	511	3,99E-02	1,14E-01	1,15	0,62	WT	bacA mutant	PA14_70160	omega amino acid--pyruvate transaminase
YP_793789.1|gi|116053462|	2	213	3,61E-01	3,67E-01	1,27	0,13	bacA mutant	WT	PA14_70180	50S ribosomal protein L33
YP_793790.1|gi|116053463|	4	198	5,69E-01	4,60E-01	1,15	0,08	bacA mutant	WT	PA14_70190	50S ribosomal protein L28
YP_793791.1|gi|116053464|	4	192	2,63E-02	9,65E-02	1,18	0,73	WT	bacA mutant	PA14_70200	dipeptide ABC transporter substrate-binding protein DppA5
YP_793794.1|gi|116053467|	7	449	7,63E-01	5,32E-01	1,02	0,06	bacA mutant	WT	PA14_70240	bifunctional phosphopantothenoylcysteine decarboxylase/phosphopantothenate synthase
YP_793796.1|gi|116053469|	21	1817	5,91E-02	1,34E-01	1,13	0,51	bacA mutant	WT	PA14_70270	phosphomannomutase
YP_793797.1|gi|116053470|	10	515	3,18E-02	1,04E-01	1,47	0,68	WT	bacA mutant	PA14_70280	acetylglutamate kinase
YP_793803.1|gi|116053476|	2	67	8,30E-01	5,49E-01	1,07	0,05	WT	bacA mutant	PA14_70350	hypothetical protein
YP_793804.1|gi|116053477|	2	156	2,11E-01	2,71E-01	1,96	0,21	WT	bacA mutant	PA14_70360	hypothetical protein
YP_793805.1|gi|116053478|	5	370	8,37E-02	1,60E-01	1,26	0,42	WT	bacA mutant	PA14_70370	orotate phosphoribosyltransferase
YP_793806.1|gi|116053479|	6	452	5,96E-04	2,67E-02	1,24	1,00	WT	bacA mutant	PA14_70390	catabolite repression control protein
YP_793807.1|gi|116053480|	2	117	5,00E-01	4,34E-01	1,67	0,09	WT	bacA mutant	PA14_70400	hypothetical protein
YP_793808.1|gi|116053481|	4	231	4,26E-01	4,02E-01	1,11	0,11	bacA mutant	WT	PA14_70420	ribonuclease PH
YP_793809.1|gi|116053482|	4	113	8,37E-01	5,50E-01	1,01	0,05	WT	bacA mutant	PA14_70430	hypothetical protein
YP_793810.1|gi|116053483|	2	78	1,28E-02	6,84E-02	1,40	0,89	WT	bacA mutant	PA14_70440	guanylate kinase
YP_793812.1|gi|116053485|	5	198	3,54E-01	3,64E-01	1,18	0,13	WT	bacA mutant	PA14_70470	guanosine-3',5'-bis(diphosphate) 3'-pyrophosphohydrolase
YP_793813.1|gi|116053486|	9	466	1,32E-01	2,08E-01	1,33	0,31	WT	bacA mutant	PA14_70480	hypothetical protein
YP_793814.1|gi|116053487|	3	193	5,28E-01	4,47E-01	1,13	0,08	WT	bacA mutant	PA14_70490	lipoprotein
YP_793817.1|gi|116053490|	3	153	4,97E-01	4,33E-01	1,09	0,09	bacA mutant	WT	PA14_70550	hypothetical protein
YP_793818.1|gi|116053491|	12	535	4,56E-02	1,19E-01	1,19	0,58	WT	bacA mutant	PA14_70560	LysR family transcriptional regulator
YP_793820.1|gi|116053493|	8	315	7,09E-01	5,18E-01	1,13	0,06	WT	bacA mutant	PA14_70580	hypothetical protein
YP_793822.1|gi|116053495|	5	389	6,96E-01	5,12E-01	1,19	0,06	WT	bacA mutant	PA14_70600	HU family DNA-binding protein
YP_793823.1|gi|116053496|	5	297	6,82E-01	5,07E-01	1,02	0,06	bacA mutant	WT	PA14_70620	rubredoxin reductase
YP_793838.1|gi|116053511|	2	183	8,00E-01	5,42E-01	1,03	0,06	WT	bacA mutant	PA14_70790	two-component response regulator

YP_793839.1|gi|116053512|	6	267	1,09E-01	1,87E-01	1,18	0,35	WT	bacA mutant	PA14_70800	phosphate uptake regulatory protein PhoU
YP_793840.1|gi|116053513|	2	60	1,65E-01	2,32E-01	1,42	0,26	bacA mutant	WT	PA14_70810	phosphate transporter ATP-binding protein
YP_793846.1|gi|116053519|	3	305	9,64E-02	1,76E-01	1,23	0,38	bacA mutant	WT	PA14_70940	choline dehydrogenase
YP_793847.1|gi|116053520|	9	641	2,61E-02	9,65E-02	1,30	0,73	bacA mutant	WT	PA14_70950	betaine aldehyde dehydrogenase
YP_793870.1|gi|116053543|	2	76	1,31E-02	6,92E-02	2,55	0,88	WT	bacA mutant	PA14_71240	hypothetical protein
YP_793872.1|gi|116053545|	3	124	8,80E-04	2,90E-02	5,35	1,00	WT	bacA mutant	PA14_71260	FMN oxidoreductase
YP_793885.1|gi|116053558|	3	139	2,87E-03	4,37E-02	7,04	1,00	WT	bacA mutant	PA14_71410	ring hydroxylating dioxygenase, alpha-subunit
YP_793888.1|gi|116053561|	4	224	3,86E-01	3,80E-01	1,08	0,12	bacA mutant	WT	PA14_71440	low specificity l-threonine aldolase
YP_793889.1|gi|116053562|	2	110	3,04E-01	3,39E-01	1,11	0,15	bacA mutant	WT	PA14_71450	hypothetical protein
YP_793896.1|gi|116053569|	4	236	1,22E-02	6,84E-02	3,10	0,89	WT	bacA mutant	PA14_71560	glutathione-independent formaldehyde dehydrogenase
YP_793897.1|gi|116053570|	4	264	8,00E-03	5,98E-02	1,12	0,95	WT	bacA mutant	PA14_71570	hypothetical protein
YP_793900.1|gi|116053573|	5	281	1,68E-01	2,33E-01	1,14	0,25	WT	bacA mutant	PA14_71600	phosphoribosylaminoimidazole carboxylase ATPase subunit
YP_793902.1|gi|116053575|	17	1216	8,60E-02	1,62E-01	1,28	0,41	WT	bacA mutant	PA14_71630	alcohol dehydrogenase
YP_793903.1|gi|116053576|	2	73	5,25E-01	4,46E-01	1,21	0,08	WT	bacA mutant	PA14_71640	LysR family transcriptional regulator
YP_793904.1|gi|116053577|	12	911	1,06E-01	1,85E-01	1,13	0,36	WT	bacA mutant	PA14_71650	aspartate ammonia-lyase
YP_793910.1|gi|116053583|	18	905	1,52E-02	7,49E-02	1,35	0,85	WT	bacA mutant	PA14_71720	pyruvate carboxylase subunit B
YP_793911.1|gi|116053584|	7	342	7,21E-01	5,19E-01	1,11	0,06	WT	bacA mutant	PA14_71740	pyruvate carboxylase subunit A
YP_793914.1|gi|116053587|	5	217	4,16E-01	3,96E-01	1,15	0,11	WT	bacA mutant	PA14_71780	RpiR family transcriptional regulator
YP_793920.1|gi|116053593|	2	111	2,70E-01	3,16E-01	1,18	0,17	WT	bacA mutant	PA14_71870	DNA-dependent helicase II
YP_793929.1|gi|116053602|	2	54	8,28E-01	5,49E-01	1,07	0,05	WT	bacA mutant	PA14_71970	GDP-mannose pyrophosphorylase
YP_793930.1|gi|116053603|	7	397	3,59E-01	3,66E-01	1,13	0,13	WT	bacA mutant	PA14_71990	GDP-mannose 4,6-dehydratase
YP_793931.1|gi|116053604|	4	182	4,23E-02	1,16E-01	1,39	0,60	WT	bacA mutant	PA14_72000	oxidoreductase Rmd
YP_793934.1|gi|116053607|	2	43	3,47E-04	2,46E-02	1,90	1,00	bacA mutant	WT	PA14_72030	hypothetical protein
YP_793936.1|gi|116053609|	2	62	9,10E-01	5,60E-01	1,05	0,05	WT	bacA mutant	PA14_72050	hypothetical protein
YP_793952.1|gi|116053625|	4	274	6,52E-01	4,95E-01	1,04	0,07	bacA mutant	WT	PA14_72260	hypothetical protein
YP_793958.1|gi|116053631|	4	196	6,04E-01	4,76E-01	1,13	0,07	WT	bacA mutant	PA14_72360	hypothetical protein

YP_793960.1|gi|116053633|	13	826	1,14E-02	6,67E-02	1,41	0,90	WT	bacA mutant	PA14_72380	two-component response regulator AlgB
YP_793962.1|gi|116053635|	2	144	6,56E-01	4,96E-01	1,12	0,07	bacA mutant	WT	PA14_72400	N-acetylmuramoyl-L-alanine amidase family protein
YP_793966.1|gi|116053639|	5	374	8,96E-03	6,17E-02	1,97	0,94	WT	bacA mutant	PA14_72450	DsbA1
YP_793967.1|gi|116053640|	5	377	9,26E-03	6,31E-02	1,75	0,93	WT	bacA mutant	PA14_72460	cytochrome c4
YP_793969.1|gi|116053642|	2	62	4,30E-02	1,16E-01	1,34	0,60	WT	bacA mutant	PA14_72480	ribosome biogenesis GTP-binding protein YsxC
YP_793970.1|gi|116053643|	3	106	9,93E-02	1,79E-01	1,81	0,37	WT	bacA mutant	PA14_72490	DNA polymerase I
YP_793972.1|gi|116053645|	2	89	2,75E-01	3,19E-01	1,18	0,17	WT	bacA mutant	PA14_72510	homoserine kinase
YP_793973.1|gi|116053646|	4	224	9,13E-01	5,62E-01	1,00	0,05	WT	bacA mutant	PA14_72520	hypothetical protein
YP_793974.1|gi|116053647|	15	1052	4,07E-01	3,90E-01	1,15	0,11	bacA mutant	WT	PA14_72540	ribonucleotide reductase
YP_793982.1|gi|116053655|	8	359	1,61E-02	7,70E-02	1,94	0,84	WT	bacA mutant	PA14_72640	TonB-dependent receptor
YP_793984.1|gi|116053657|	3	157	1,06E-02	6,67E-02	1,70	0,91	bacA mutant	WT	PA14_72660	hypothetical protein
YP_793985.1|gi|116053658|	2	119	5,58E-04	2,67E-02	3,36	1,00	bacA mutant	WT	PA14_72690	glutamine synthetase
YP_793999.1|gi|116053672|	6	291	6,53E-02	1,40E-01	1,27	0,48	WT	bacA mutant	PA14_72840	short-chain dehydrogenase
YP_794000.1|gi|116053673|	2	96	8,61E-01	5,53E-01	1,02	0,05	bacA mutant	WT	PA14_72850	glutamine synthetase
YP_794001.1|gi|116053674|	5	327	4,44E-01	4,10E-01	1,09	0,10	WT	bacA mutant	PA14_72870	aminotransferase
YP_794006.1|gi|116053679|	6	310	7,43E-01	5,27E-01	1,12	0,06	WT	bacA mutant	PA14_72930	hypothetical protein
YP_794023.1|gi|116053696|	8	454	1,56E-04	2,46E-02	3,45	1,00	WT	bacA mutant	PA14_73120	hypothetical protein
YP_794027.1|gi|116053700|	8	566	8,55E-01	5,53E-01	1,03	0,05	bacA mutant	WT	PA14_73170	glucosamine--fructose-6-phosphate aminotransferase
YP_794030.1|gi|116053703|	9	417	9,50E-01	5,72E-01	1,00	0,05	WT	bacA mutant	PA14_73220	glucosamine-1-phosphate acetyltransferase/N-acetylglucosamine-1-phosphate uridyltransferase
YP_794031.1|gi|116053704|	4	229	1,36E-01	2,11E-01	1,31	0,30	WT	bacA mutant	PA14_73230	F0F1 ATP synthase subunit epsilon
YP_794032.1|gi|116053705|	37	3911	8,66E-03	6,09E-02	1,36	0,94	WT	bacA mutant	PA14_73240	F0F1 ATP synthase subunit beta
YP_794033.1|gi|116053706|	18	1225	9,49E-01	5,72E-01	1,00	0,05	bacA mutant	WT	PA14_73250	F0F1 ATP synthase subunit gamma
YP_794034.1|gi|116053707|	30	2446	2,32E-02	9,26E-02	1,31	0,76	WT	bacA mutant	PA14_73260	F0F1 ATP synthase subunit alpha
YP_794035.1|gi|116053708|	4	161	5,74E-02	1,33E-01	1,58	0,52	WT	bacA mutant	PA14_73280	F0F1 ATP synthase subunit delta
YP_794036.1|gi|116053709|	9	713	4,53E-01	4,15E-01	1,23	0,10	WT	bacA mutant	PA14_73290	F0F1 ATP synthase subunit B
YP_794038.1|gi|116053711|	3	217	9,97E-01	5,84E-01	1,19	0,05	WT	bacA mutant	PA14_73310	F0F1 ATP synthase subunit A

YP_794040.1|gi|116053713|	14	927	8,54E-01	5,53E-01	1,01	0,05	WT	bacA mutant	PA14_73330	chromosome partitioning protein Spo0J
YP_794041.1|gi|116053714|	4	252	5,98E-04	2,67E-02	1,42	1,00	WT	bacA mutant	PA14_73350	chromosome partitioning protein Soj
YP_794043.1|gi|116053716|	2	52	1,58E-01	2,26E-01	1,20	0,27	bacA mutant	WT	PA14_73370	tRNA uridine 5-carboxymethylaminomethyl modification protein GidA
YP_794045.1|gi|116053718|	3	117	3,22E-01	3,49E-01	1,20	0,14	WT	bacA mutant	PA14_73400	tRNA modification GTPase TrmE
YP_794046.1|gi|116053719|	6	354	8,56E-01	5,53E-01	1,01	0,05	WT	bacA mutant	PA14_73410	inner membrane protein translocase component YidC
