# Supplementary material for: BacA: a possible regulator that contributes to the biofilm formation of Pseudomonas aeruginosa
Source: Front Microbiol. 2024 Mar 5;15:1332448. doi: 10.3389/fmicb.2024.1332448 (PMC10948618; doi:10.3389/fmicb.2024.1332448)
Supplement: Supplementary file 4 [file Table_4.pdf]

Table S4. Structural homology search using I-TASSER with BacA as query.

Position	PDB	TM-score	RMSD	Description	Organism	DOI
1	1jyo	0.792	1.88	SicP, T3SS chaperone	*Salmonella enterica*	10.1038/35102073
2	6vu7	0.732	2.55	YbjN, putative transcription regulator	*Escherichia coli*	10.1371/journal.pone.0025293
3	3kxy	0.723	2.44	ExsC, T3SS chaperone/transcription regulator	*Pseudomonas aeruginosa*	10.1021/bi100432e
4	5fr7	0.718	2.77	AmyR, amylovoran repressor, YbjN protein family	*Erwinia amylovora*	10.1371/journal.pone.0176049
5	3epu	0.703	2.50	STM2138, T3SS chaperone	*Salmonella enterica*	
6	4g6t	0.7	2.40	ShcA, T3SS chaperone	*Pseudomonas syringae*	10.1128/JB.01621-12
7	2plg	0.68	2.79	T110839, putative sensory transduction regulator	*Synechococcus elongatus*	10.1074/jbc.M400077200
8	4h5b	0.678	2.62	DR_1245, YbjN domain-containing protein	*Deinococcus radiodurans*	10.1371/journal.pone.0056558
9	2fm8	0.670	3.01	InvB, T3SS chaperone	*Salmonella enterica*	10.1016/j.molcel.2006.01.026
10	1ry9	0.653	3.07	Spa15, T3SS chaperone	*Shigella flexneri*	10.1038/sj.embor.7400144
