# Supplementary material for: BacA: a possible regulator that contributes to the biofilm formation of Pseudomonas aeruginosa
Source: Front Microbiol. 2024 Mar 5;15:1332448. doi: 10.3389/fmicb.2024.1332448 (PMC10948618; doi:10.3389/fmicb.2024.1332448)
Supplement: Supplementary file 5 [file Image_1.pdf]

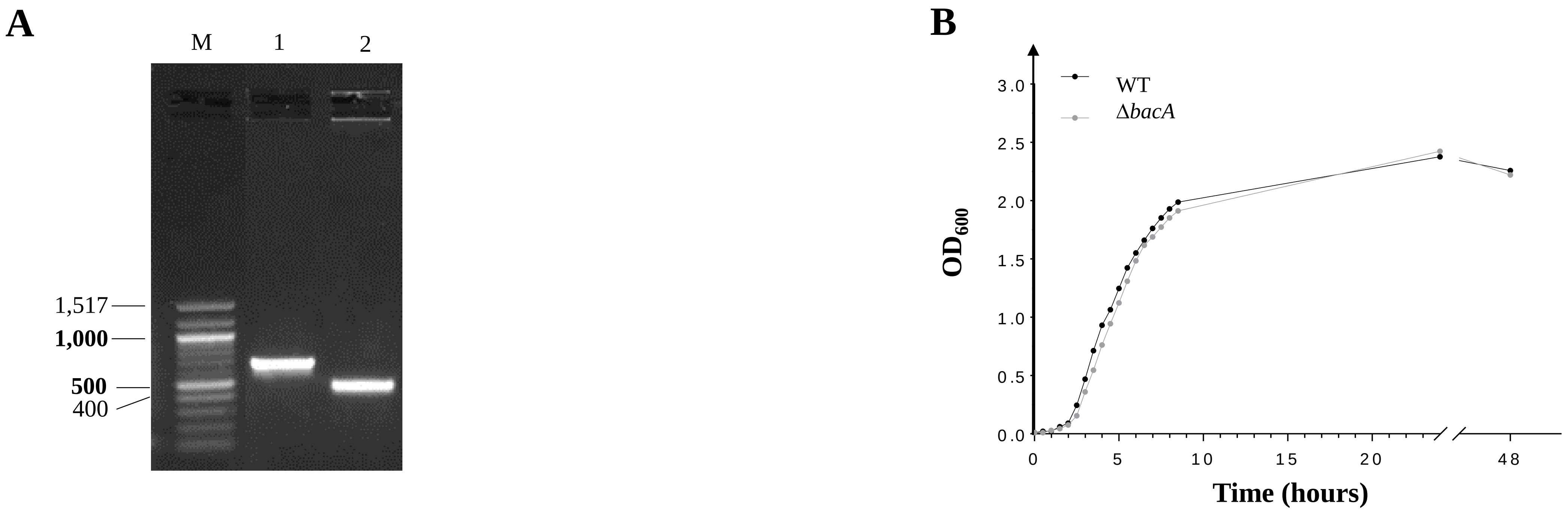

Supplementary Figure 1. Verification of the Δ*bacA*.

(A) MARxT7 transposon insertion verification. Lane 1: *bacA* mutated, lane 2: wild-type *bacA*, lane M: 100 bp ladder molecular size marker. **(B)** Growth curves of the WT and Δ*bacA* strains in LB under 140 rpm at 37°C. Data represent mean values (±SEM) from three independent biological experiments.
