# Supplementary material for: BacA: a possible regulator that contributes to the biofilm formation of Pseudomonas aeruginosa
Source: Front Microbiol. 2024 Mar 5;15:1332448. doi: 10.3389/fmicb.2024.1332448 (PMC10948618; doi:10.3389/fmicb.2024.1332448)
Supplement: Supplementary file 6 [file Image_2.pdf]

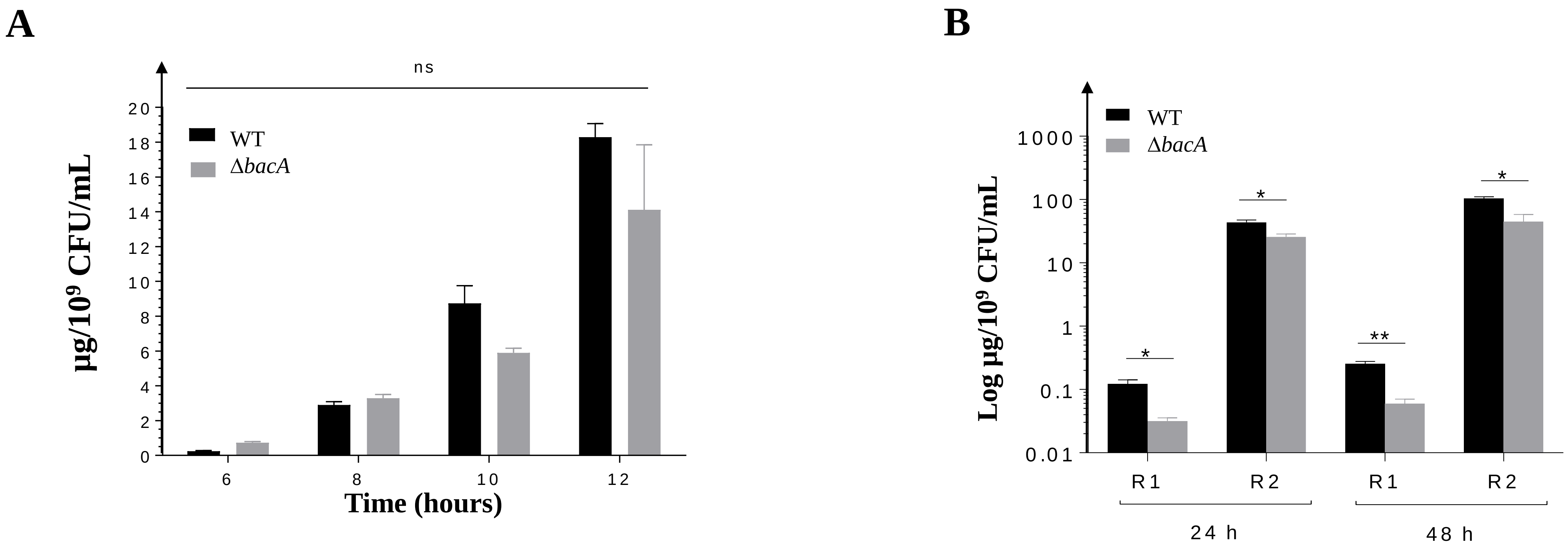

Supplementary Figure 2. Impact of the *bacA* mutation on the rhamnolipids secretion.

(A) Impact of the *bacA* mutation on rhamnolipid production. Rhamnolipids were detected by LC-MS/MS from supernatants of LB culture at specific times of growth. Data represent mean values (±SEM) from three independent biological experiments (ns, non-significant, 2way ANOVA test)). **(B)** Amount (log scale) of the two most abundant rhamnolipids, *i.e* the Rha-C_{10}-C_{10} (R1) and the Rha-Rha-C_{10}-C_{10} (R2). Data represent mean values (±SEM) from three independent biological experiments (**p=0.01 ; *p<0.05, Student's t-test).
