# Supplementary material for: BacA: a possible regulator that contributes to the biofilm formation of Pseudomonas aeruginosa
Source: Front Microbiol. 2024 Mar 5;15:1332448. doi: 10.3389/fmicb.2024.1332448 (PMC10948618; doi:10.3389/fmicb.2024.1332448)
Supplement: Supplementary file 7 [file Image_3.pdf]

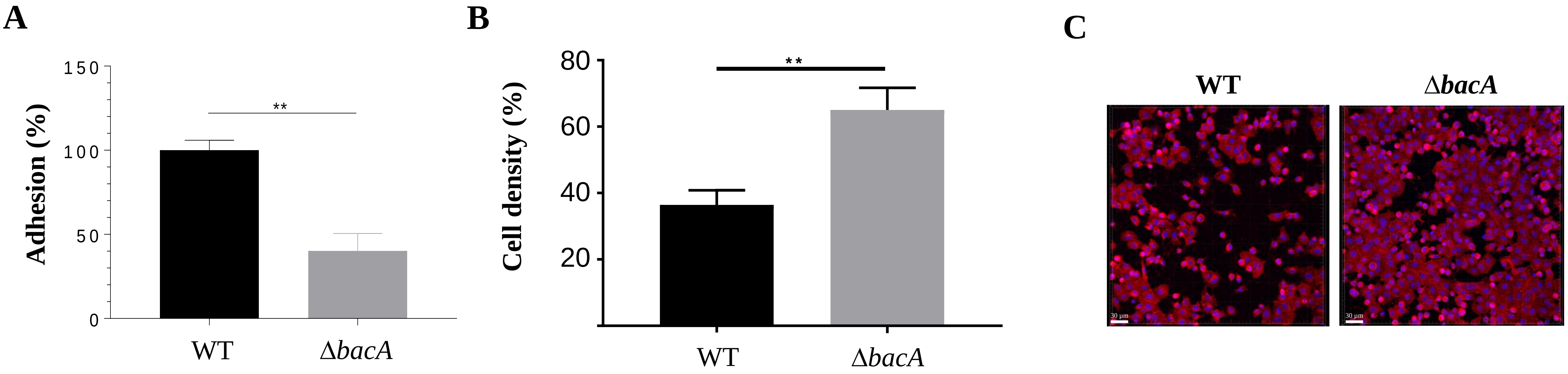

Supplementary Figure 3. The Δ*bacA* exhibits limited invading capacity.

(A) Percentage of adhesion after 2 h of contact with the WT or the mutant. Data shown represent mean values (±SEM) from at least eight independent biological experiments (**p<0.01). **(B)** Quantification of A549 cellular density after 2 h of infection with *P. aeruginosa* WT and *bacA* mutant, based on fluorescence measurements from confocal microscopy images. Quantification were realised from at least ten acquisitions from four independent biological experiments (**p<0,01). **(C)** Representative CLSM images of A549 cell layers after the 2 hours of incubation with the WT or the mutant. The nucleus (in blue) and actin (in pink) were labeled with DAPI and phalloidin rhodamine, respectively.
