# Supplementary material for: BacA: a possible regulator that contributes to the biofilm formation of Pseudomonas aeruginosa
Source: Front Microbiol. 2024 Mar 5;15:1332448. doi: 10.3389/fmicb.2024.1332448 (PMC10948618; doi:10.3389/fmicb.2024.1332448)
Supplement: Supplementary file 8 [file Image_4.pdf]

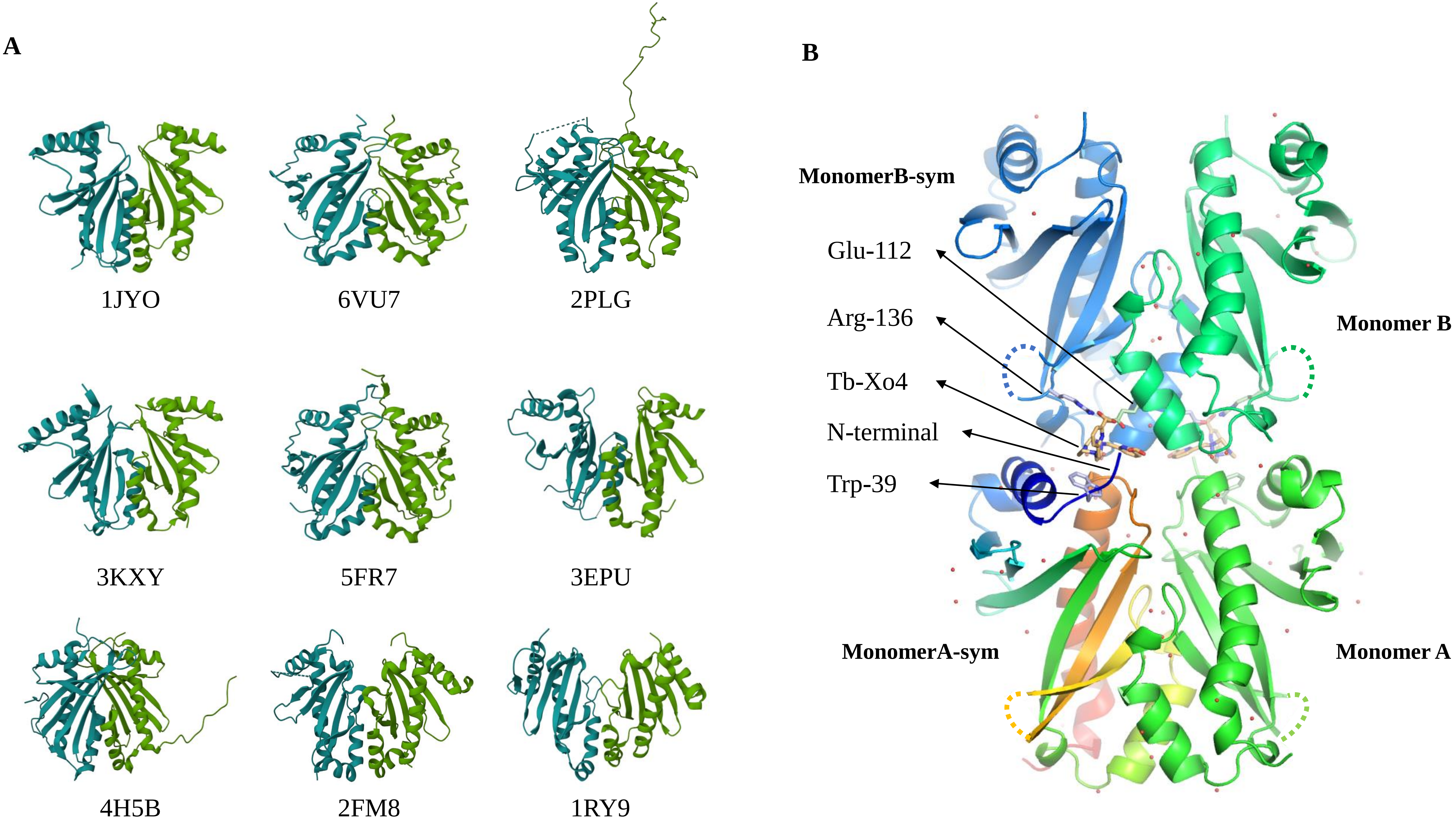
A
1JYO
6VU7
2PLG
3KXY
5FR7
3EPU
4H5B
2FM8
1RY9
B
MonomerB-sym
Glu-112
Arg-136
Tb-Xo4
N-terminal
Trp-39
Monomer B
MonomerA-sym
Monomer A

Supplementary Figure 4. (A) Comparison of the BacA 3D-structure obtained by crystallography with numerous secretion type-III chaperones or genetic regulator proteins. The PDB numbers and structures presented were obtained from the PDB website (https://www.rcsb.org/). The protein ShcA (4gt6) is not represented because it is not available in dimer without its interacting ligand. **(B)** Structure of the two monomers of BacA present in the crystallographic asymmetric unit represented in two different green. The two dimers are reconstituted by application of the first order symmetry axis of the space group $P6_522$ (symmetry-generated monomer B called monomerB-sym (blue) and symmetry-generated monomer A called monomerA-sym (rainbow). Molecules of Tb-Xo4 are represented in light-pink sticks. The amino-acids making direct contacts with Tb-Xo4 are represented in sticks, in the same color as the monomer they belong to (N-terminus and Trp-39 from the monomerA-sym in blue, Glu-112 from molecule B in green, and Arg-136 from the monomer-sym in blue).
